# Treacle-dependent TOPBP1 condensation regulates the nucleolar DNA damage response

**DOI:** 10.1093/nar/gkag350

**Published:** 2026-05-19

**Authors:** Nadezhda V Petrova, Dmitry A Deriglazov, Anastasia P Kovina, Artem V Luzhin, Victoria O Shender, Georgij P Arapidi, Alexey S Gavrikov, Alexander S Mishin, Sergey V Razin, Artem K Velichko

**Affiliations:** Department of Cellular Genomics, Institute of Gene Biology RAS, 119334 Moscow, Russia; Department of Cellular Genomics, Institute of Gene Biology RAS, 119334 Moscow, Russia; Department of Cellular Genomics, Institute of Gene Biology RAS, 119334 Moscow, Russia; Department of Cellular Genomics, Institute of Gene Biology RAS, 119334 Moscow, Russia; Shemyakin-Ovchinnikov Institute of Bioorganic Chemistry of the Russian Academy of Sciences, 117997 Moscow, Russia; Lopukhin Federal Research and Clinical Center of Physical-Chemical Medicine of Federal Medical Biological Agency, 119435 Moscow, Russia; Shemyakin-Ovchinnikov Institute of Bioorganic Chemistry of the Russian Academy of Sciences, 117997 Moscow, Russia; Lopukhin Federal Research and Clinical Center of Physical-Chemical Medicine of Federal Medical Biological Agency, 119435 Moscow, Russia; Shemyakin-Ovchinnikov Institute of Bioorganic Chemistry of the Russian Academy of Sciences, 117997 Moscow, Russia; Shemyakin-Ovchinnikov Institute of Bioorganic Chemistry of the Russian Academy of Sciences, 117997 Moscow, Russia; Department of Cellular Genomics, Institute of Gene Biology RAS, 119334 Moscow, Russia; Biological Faculty, Lomonosov Moscow State University, 119234 Moscow, Russia; Department of Cellular Genomics, Institute of Gene Biology RAS, 119334 Moscow, Russia; Institute for Translational Medicine and Biotechnology, Sechenov First Moscow State Medical University, 119991 Moscow, Russia

## Abstract

Ribosomal DNA (rDNA) arrays are among the most highly transcribed repetitive regions of the genome and therefore require specialized mechanisms to maintain their stability. The nucleolar DNA damage response (n-DDR) safeguards rDNA integrity and is coordinated by the repair scaffold TOPBP1 and the fibrillar center (FC) protein Treacle. Here, we show that Treacle promotes phase separation of TOPBP1 within the FC to establish a spatially confined signaling platform that amplifies nucleolar DDR signaling and coordinates rDNA repair pathway engagement. Using an inducible TOPBP1 oligomerization system together with physiological models of rDNA damage, we demonstrate that phosphorylation of Treacle by CK2 and ATR/ATM enables its interaction with TOPBP1 and nucleates TOPBP1 condensation in the nucleolus. Functionally, Treacle-dependent condensation promotes robust ATR/ATM activation, γH2AX signaling, and recruitment of DNA repair factors. Disruption of this process does not impair the initial removal of rDNA double-strand breaks but shifts repair toward rapid DNA-PK-dependent nonhomologous end joining while attenuating ATR/ATM signaling and reducing engagement of homologous recombination-associated pathways. Treacle-knockout cells exhibit accelerated early rDNA repair but incomplete damage resolution at later stages. Together, our findings identify Treacle-dependent TOPBP1 condensation as a nucleolar signaling platform that promotes accurate maintenance of highly repetitive rDNA arrays.

## Graphical Abstract

**Graphical Abstract:**
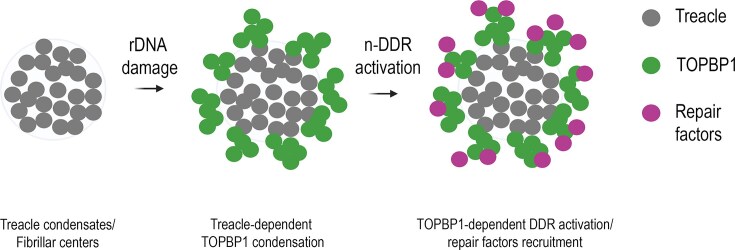

## Introduction

To safeguard genomic integrity, cells have evolved a complex signaling network known as the DNA damage response (DDR), which detects and repairs aberrant DNA structures [1, 2]. Over the past few decades, extensive research has both significantly advanced our understanding of the DDR and uncovered new layers of complexity that warrant further investigation. Specifically, it has become increasingly clear that the DDR is not uniform across the genome and instead is modulated by chromatin context and subnuclear localization [3, 4].

In this context, the nucleolus represents a unique and particularly intriguing compartment due to its distinct physicochemical organization and chromatin composition. The nucleolus is a specialized nuclear domain responsible for ribosomal RNA (rRNA) synthesis and ribosome assembly. It is formed via the complex coacervation of its components, which organize into three immiscible, condensed liquid phases: fibrillar centers (FCs), the dense fibrillar component (DFC), and the granular component [5–7]. While this phase-separated architecture optimizes rRNA transcription and processing, it also renders ribosomal DNA (rDNA) particularly vulnerable to genomic insults. This vulnerability arises from multiple factors. First, the high transcriptional activity of rDNA increases the likelihood of collisions between the transcriptional and replication machineries, leading to the formation of R-loops and G-quadruplexes that promote DNA double-strand breaks (DSBs) and genomic instability [8–12]. Second, the repetitive nature of rDNA sequences enhances the potential for recombination, facilitating rDNA copy number variation and chromosomal rearrangements [13–15]. Third, the coacervate nature of the nucleolus may be less accessible to canonical repair proteins such as those involved in homologous recombination (HR) and nonhomologous end joining (NHEJ), necessitating specialized mechanisms for rDNA repair [11, 16].

Indeed, recent studies have uncovered nucleolus-specific DDR pathways, collectively referred to as the nucleolar DDR (n-DDR) [11, 17–19]. These pathways involve nucleolar transcriptional silencing and either promote rapid rDNA repair or, in cases of persistent damage, drive nucleolar reorganization to enable HR [17, 20–26]. A key difference between canonical and n-DDR lies in the central roles of two proteins: the nucleolar scaffold protein Treacle and the canonical DDR effector TOPBP1. Treacle is an intrinsically disordered, nucleolar phosphoprotein that serves as a structural scaffold for FCs. Through its phase-separating properties, Treacle concentrates transcriptional machinery at rDNA loci to facilitate efficient rRNA synthesis and spatially separates FCs and DFCs to support pre-rRNA processing [3, 7]. Following specific types of rDNA damage, such as DSBs, replication stress, and R-loop stabilization, Treacle recruits the repair protein TOPBP1, thereby initiating n-DDR signaling, recruiting repair factors to rDNA, and facilitating DNA repair [7, 10, 23, 25]. In contrast, TOPBP1 is best known for its role in canonical replication stress-induced DDR signaling [27, 28]. Specifically, its ability to undergo biomolecular condensation and phase separation enables the formation of repair condensates that serve as scaffolds for ATR/Chk1 activation and transduction of DNA damage signals throughout the nucleus [29, 30]. However, the function of TOPBP1 within the context of n-DDR, as well as the molecular mechanisms underlying its interaction with Treacle, remain poorly understood.

In this study, we provide new insights into the molecular mechanisms underlying Treacle–TOPBP1 interactions during the nucleolar DNA damage response (n-DDR). Specifically, we demonstrate that cooperative phosphorylation of Treacle at Ser1191 and Ser1199 by CK2 and ATR/ATM kinases, respectively, is a critical prerequisite for this interaction. These modifications enable Treacle to engage in bivalent interactions with BRCA1 C-terminal (BRCT) domains 2 and 5 of TOPBP1, thereby nucleating the formation of heterotypic condensates between Treacle and TOPBP1. Using both a reductionist inducible TOPBP1 oligomerization system and physiological models of rDNA damage, we show that TOPBP1 phase separation is initiated at Treacle scaffolds and maintained through a combination of specific domain interactions and weak multivalent contacts, consistent with the principles of liquid–liquid phase separation (LLPS).

Importantly, our results indicate that Treacle-dependent TOPBP1 condensation functions as a nucleolar signaling platform that amplifies ATR/ATM-dependent DDR signaling rather than serving as the primary sensor of rDNA DSBs. Disruption of this condensate does not abolish early rDNA break removal but alters the balance of repair pathway engagement, favoring rapid DNA-PK-dependent nonhomologous end joining while reducing recruitment of HR-associated repair mechanisms. Depending on the nature of genotoxic stress, Treacle–TOPBP1 condensates either promote γH2AX signaling, transcriptional repression, and nucleolar cap formation to facilitate ATR/ATM-driven repair, or scaffold repair factors at nucleolar TOPBP1 assemblies without fully suppressing rRNA transcription.

Thus, our findings reveal Treacle–TOPBP1 condensates as a dynamic regulatory hub that amplifies DNA damage signaling and coordinates the engagement of high-fidelity repair pathways at rDNA loci. More broadly, this work highlights phase separation as a key organizing principle underlying the spatial and functional specialization of the n-DDR.

## Materials and methods

### Cell culture and drug treatment

Human HeLa (ATCC^®^ CCL-2™), HCT116 (kindly provided by Prof. Boris Zhivotovsky, Karolinska Institut, Stockholm, Sweden), MCF7 (ATCC^®^HTB-22), HEK293 (ATCC^®^ CRL-1573™), HAP1 (kindly provided by Dr N. Batulin, Institute of Cytology and Genetics, Novosibirsk, Russia), and human skin fibroblasts (female 46XX) were cultured in Dulbecco's Modified Eagle Medium (DMEM; PanEco) supplemented with 10% fetal bovine serum (FBS; HyClone/GE Healthcare) and penicillin/streptomycin. The cells were cultured at 37°C in a conventional humidified CO_2_ incubator.

DNA damage was induced by the treatment of cells with 90 µM etoposide (VP16; Sigma–Aldrich, #E1383), 300 µM hydrogen peroxide (H_2_O_2_; Sigma–Aldrich, #H1009), 50 µM mitomycin c (MMC; Sigma–Aldrich, #M4287), or 50 µM cisplatin (CIS; Sigma–Aldrich, #C2210000) for 30 min or 3 h. Replication stress was induced by the treatment of cells with 1 µM aphidicolin (APH; Sigma–Aldrich, #A0781) for 6–16 h. Hypoosmotic stress was applied by incubation the cells in 50% DMEM/50% H_2_O for 10 min to 3 h. I-PPOI nuclear translocation was initiated by incubating the cells with 1 µM 4-hydroxytamoxifen (4-OHT; Sigma–Aldrich, #T176) for 30 min or 3 h. For PIKK kinase inhibition experiments, cells were treated with 20 µM KU55933 (ATMi; Tocris Bioscience, #3544) for 3 h, 15 µM VE821 (ATRi; Sigma–Aldrich, #SML1415) for 3–6 h or 20 µM NU7441 (DNA-PKi; Sigma–Aldrich, #503468-95-9) for 3 h. For CK2 inhibition, the cells were treated with 30 µM CX-4945 (CK2i; Selleckchem, #S2248) for 3 h. For induction of TOPBP1 oligomerization, transfected cells were incubated with 100 nM AP20187 (Sigma–Aldrich, #SML2838) for 24 h. For TOPBP1 oligomerization inhibition cells were incubated with 5 µM calcein AM (Sigma–Aldrich, #56496) for 5 h.

To obtain 1,6-hexanediol (1,6-HEX)-treated cells, HeLa cells were incubated with 5% 1,6-HEX (Sigma–Aldrich, #240117) in serum-free medium at 37°C in a humidified atmosphere for 10 min. To obtain ammonium acetate-treated cells, HeLa cells were incubated with 100–200 mM ammonium acetate (AmAc) in a complete culture medium at room temperature (RT) for 5 min. To obtain sorbitol- or sodium chloride (NaCl)-treated cells, HeLa cells were incubated with 300 mM sorbitol or 300 mM NaCl, respectively, in a complete culture medium at 37°C in a humidified atmosphere for 30 min.

### Plasmid constructs and transfection

To generate pTreacle-GFP or pTreacle-2S constructs, the full-length Treacle was amplified by polymerase chain reaction (PCR) from cDNA with primer set #1 (Supplementary Table 1) using KAPA High-Fidelity DNA Polymerase (KAPA Biosystems, KE2502). The forward and reverse primers contained BglII and BamHI sites, respectively. The amplified fragment was inserted into the pTurboGFP-C (Evrogen, FP511) or pKatushka2S-C (Evrogen, FP761) vectors using BglII/BamHI restriction/ligation.

The pTreacle-2S S1190A/S1190A was constructed based on the pTreacle-2S plasmid using iProof High-Fidelity DNA polymerase with primer set #2 (Supplementary Table 1). The resulting DNA template after PCR was reprecipitated and treated with the DpnI restriction enzyme. In the next step, the desired DNA template was purified on an agarose gel, phosphorylated with T4 Polynucleotide Kinase (T4 PNK), and ligated.

The pTreacle-2S S1199A was constructed based on the pTreacle-2S plasmid using iProof High-Fidelity DNA polymerase with primer set #3 (Supplementary Table 1). The resulting DNA template after PCR was reprecipitated and treated with the DpnI restriction enzyme. In the next step, the desired DNA template was purified on an agarose gel, phosphorylated with T4 Polynucleotide Kinase (T4 PNK), and ligated.

To make pTOPBP1-2S or pTOPBP1-GFP constructs, the full-length TOPBP1 was generated by PCR amplification with primer set #4 (Supplementary Table 1) from the pcDNA3-LacR-TOPBP1 plasmid (Addgene, #31 317) and was inserted into the pTurboGFP-C (Evrogen, FP511) or pKatushka2S-C (Evrogen, FP761) vectors linearized with SacII.

The pTOPBP1-FLAG-FKBP was constructed based on the pTOPBP1-GFP plasmid. In the first step, GFP was excised from the pTOPBP1-GFP plasmid, and a 1 × FLAG tag was introduced at the N-terminus using iProof High-Fidelity DNA polymerase and primer set #5 (Supplementary Table 1). In the second step, the stop codon was removed from the resulting pTOPBP1 construct using primer set #6 (Supplementary Table 1). In the third step, a DNA fragment encoding FKBP12-F36V, a new stop codon, and restriction sites for BamHI and SacII was amplified by PCR from the pENTR1A FKBP12F36V-OGT (Addgene, #154 287) using primer set #7 (Supplementary Table 1). In the final step, this fragment was inserted into the modified pTOPBP1 plasmid at the BamHI and SacII restriction sites.

The pTOPBP1-FLAG-FKBP ΔBRCT2 was constructed based on the pTOPBP1-FLAG-FKBP plasmid using iProof High-Fidelity DNA polymerase with primer set #8 (Supplementary Table 1). The resulting DNA template after PCR was reprecipitated and treated with the DpnI restriction enzyme. In the next step, the desired DNA template was purified on an agarose gel, phosphorylated with T4 Polynucleotide Kinase (T4 PNK), and ligated.

The pTOPBP1-FLAG-FKBP ΔBRCT5 was constructed based on the pTOPBP1-FLAG-FKBP plasmid using iProof High-Fidelity DNA polymerase with primer set #9 (Supplementary Table 1). The resulting DNA template after PCR was reprecipitated and treated with the DpnI restriction enzyme. In the next step, the desired DNA template was purified on an agarose gel, phosphorylated with T4 Polynucleotide Kinase (T4 PNK), and ligated.

The pTOPBP1-FLAG-FKBP ΔBRCT7/8 was constructed based on the pTOPBP1-FLAG-FKBP plasmid using iProof High-Fidelity DNA polymerase with primer set #10 (Supplementary Table 1). The resulting DNA template after PCR was reprecipitated and treated with the DpnI restriction enzyme. In the next step, the desired DNA template was purified on an agarose gel, phosphorylated with T4 Polynucleotide Kinase (T4 PNK), and ligated.

The pTOPBP1-FLAG-FKBP ΔAAD was constructed based on the pTOPBP1-FLAG-FKBP plasmid using iProof High-Fidelity DNA polymerase with primer set #11 (Supplementary Table 1). The resulting DNA template after PCR was reprecipitated and treated with the DpnI restriction enzyme. In the next step, the desired DNA template was purified on an agarose gel, phosphorylated with T4 Polynucleotide Kinase (T4 PNK), and ligated.

Plasmids encoding TOPBP1-FLAG constructs lacking individual BRCT domains (ΔBRCT1 through ΔBRCT8) were generously provided by Prof. Jiadong Wang (Peking University Health Science Center, Beijing, China).

For IPpo-I rDNA damage induction, plasmid pBABe-HA-ER-IppoI (Addgene, #32 565) was used.

All transection experiments were performed using Lipofectamine LTX transfection reagent (Thermo fisher scientific, #15 338 100) following the manufacturer’s instructions.

### Gene knockout

For CRISPR/Cas9-mediated knockout, two single guide RNAs (sgRNA) flanking a region of the target gene (*H2AX* or *TOPBP1*) or to first exon of the *TCOF1* were designed using the guide RNA design tool (www.atum.bio/eCommerce/cas9/input). The sgRNA targeting sequences were separately cloned into the sgRNA/Cas9 expression vector pSpCas9n(BB)-2A-Puro (PX462) V2.0 (Addgene #62 987). A list of all oligonucleotides is provided in Supplementary Table 2. The plasmids were co-transfected into HeLa cells with Lipofectamine LTX transfection reagent (Thermo Fisher Scientific, #15338100). The transfectants were selected with 10 µg/ml puromycin for 24 h. After 24 h of puromycin selection, cells were switched to their normal culture medium. Clones of HeLa cells were obtained by limiting dilution or cell sorting using an SH800 Cell Sorter (Sony) into 96-well plates. Western blotting and indirect immunofluorescence was used to identify clones with H2AX, Treacle, or TOPBP1 depletion.

### Whole-cell extracts preparation and immunoblotting

Cells were lysed by incubation in Radioimmunoprecipitation assay buffer (RIPA) [150 mM NaCl, 1% Triton X-100, 0.5% sodium deoxycholate, 0.1% sodium dodecyl sulphate (SDS), 50 mM Tris–HCl (pH 8.0) supplemented with Protease Inhibitor Cocktail (Bimake), and Phosphatase Inhibitor Cocktail (Bimake)] for 30 min on ice. Next, the cell extracts were sonicated with a VirSonic 100 ultrasonic cell disrupter and stored at −70°C. The protein concentration was measured by the Bradford assay. Aliquots of each sample were separated by sodium dodecyl sulfate-polyacrylamide gel electrophoresis and blotted onto polyvinylidene difluoride (PVDF) membranes (Amersham/GE Healthcare). The membranes were blocked for 1 h in 2% ECL Advance blocking reagent (GE Healthcare) or 2% bovine serum albumin (BSA; Sigma–Aldrich) in PBS containing 0.1% Tween 20 (PBS-T) followed by incubation overnight at 4°C with a primary antibody diluted in PBS-T containing 2% blocking reagent or 2% BSA. After three washes with PBS-T, the membranes were incubated for 1 h with the secondary antibodies (horseradish peroxidase-conjugated anti-rabbit or anti-mouse IgG) in PBS-T containing 2% blocking agent or 2% BSA. The immunoblots were visualized using a Pierce ECL plus western blotting substrate. Antibodies used in the study are listed in Supplementary Table 3.

### Fluorescence microscopy

For immunostaining, cells were grown on microscope slides. All samples were fixed in CSK buffer (10 mM PIPES, pH 7.0, 100 mM NaCl, 1.5 mM MgCl_2_, 300 mM sucrose) supplemented with 1% paraformaldehyde (PFA) and 2.5% Triton X-100 for 15 min at RT. Cells were washed in PBS and then incubated with antibodies in PBS supplemented with 1% BSA and 0.05% Tween 20 for 1 h at RT or overnight at 4°C. Then the cells were washed with PBS three times (5 min each time). The primary antibodies bound to antigens were visualized using Alexa Fluor 488-conjugated, Alexa Fluor 594-conjugated or Alexa Fluor 647-conjugated secondary antibodies. The DNA was counterstained with the fluorescent dye 4,6-diamino-2-phenylindole (DAPI) for 10 min at RT. The samples were mounted using Dako fluorescent mounting medium (Life Technologies). The immunostained samples were analyzed using a Zeiss AxioScope A.1 fluorescence microscope (objectives: Zeiss N-Achroplan 40×/0.65 and EC Plan-Neofluar 100×/1.3 oil; camera: Zeiss AxioCam MRm; acquisition software: Zeiss AxioVision Rel. 4.8.2; Jena, Germany) or STELLARIS 5 Leica confocal microscope (objectives: HC PL APO 63×/1.40 oil CS2). The images were processed using ImageJ software (version 1.44) and analyzed using CellProfiler software (version 3.1.5). 3D reconstruction of *xyz* confocal datasets (*z*-stacks) was performed using Leica LAS-X software. Antibodies used in the study are listed in Supplementary Table 3.

For STORM microscopy, cells were grown on 35 mm imaging dishes (Ibidi). All samples were fixed in CSK buffer (10 mM PIPES, pH 7.0, 100 mM NaCl, 1.5 mM MgCl2, 300 mM sucrose) supplemented with 1% PFA and 2.5% Triton X-100 for 15 min at RT. After washing once with 1× PBS, the cells were incubated with TOPBP1 and Treacle antibodies diluted in blocking buffer (1% BSA in 1 × PBS with 0.05% Tween 20), at 4°C overnight. The cells were washed three times with 1 × PBS, for 5 min per wash, and the Alexa Fluor 647- and Atto 488-conjugated secondary antibody in the blocking buffer was added to the sample for 1 h, protected from light. The cells were washed three times with 1× PBS and stored in 1× PBS before imaging. Immediately before imaging, the buffer was replaced with STORM imaging buffer, containing 10% (w/v) glucose (Sigma–Aldrich), 50 mM Tris–HCl, pH 8.0, 10 mM NaCl, 0.56 mg/ml glucose oxidase (Sigma–Aldrich), 0.17 mg/ml catalase (Sigma–Aldrich), and 0.14 M β-mercaptoethanol (Sigma–Aldrich). All imaging experiments were performed using a commercial ONI Nanoimager microscope system. The emitted light was collected by an oil immersion 100×, 1.49 NA objective and imaged onto a scientific CMOS (sCMOS) camera. For STORM imaging, 50 000 frames were acquired at an exposure time of 10 ms. The reconstruction of the super-resolution image was performed using NimOS software from ONI.

### Live-cell imaging

Cells were seeded in 35 mm imaging dishes (Ibidi) 12 h before transfection with the plasmid. Twenty-four hours after transfection, Hoechst 33 342 (Cell Signaling Technology) was added to the medium at a final concentration of 1 μg/ml, and the cells were incubated for 20 min at 37°C. Hoechst-containing medium was replaced with a phenol red-free DMEM medium supplemented with 10% FBS. The cells underwent live-cell imaging using a STELLARIS 5 Leica confocal microscope equipped with an incubation chamber to provide a humidified atmosphere at 37°C with 5% CO_2_. For observation of Treacle and TOPBP1 condensates behavior, z-stack time-lapses were taken. Maximum intensity projections are shown, but all findings were confirmed on a single plane.

### Fluorescence recovery after photobleaching analysis

Fluorescence recovery after photobleaching (FRAP) experiments were performed on a STELLARIS 5 Leica confocal microscope equipped with an HC PL APO 63×/1.40 oil CS2 objective and an incubation chamber that maintained a humidified atmosphere at 37°C with 5% CO₂. Live-cell experiments were performed using transfected cells cultured in phenol red-free DMEM medium supplemented with 10% FBS. Imaging was typically performed at a resolution of 512 × 512 pixels, with a scan speed of 200 ms per frame.

Typically, images were acquired at 512 × 512 pixels at a scan speed corresponding to 200 ms per image. Before photobleaching, 3 to 5 images were recorded. Bleaching parameters, i.e. laser intensity and scanning time, were chosen to reach approximate 50% of bleaching in the shortest possible time. The bleaching area was selected according to the type of experiment (full-or half-FRAP). In the case of half-FRAP, which relies on the analysis of the bleached and the nonbleached half, it is important to optimize the bleaching step (intensity and area of the bleaching spot) to minimize bleaching in the other (nonbleached) half. Half-FRAP experiments were conducted until the signals in both halves had converged to each other or until the signal in the nonbleached half had reached its initial pre-bleach value. FRAP data analysis was performed as described in [31].

### Cell sorting

For chromatin immunoprecipitation (ChIP) analysis twenty-four hours after transfection cells were trypsinized, fixed 1% PFA in 1 × PBS for 15 min, then washed three times with 1 × PBS and passed through a 40 mm filter (BD Falcon 352340) to remove cell aggregates. Cell sorting was performed using an SH800 Cell Sorter (Sony) with a laser tuned to 561 nm for 2S fluorescence. Gates were set with reference to negative controls. A minimum of 2 × 10^6^ events was collected for each analysis. The sorted cell fractions were resuspended in a 1 × PBS and proceeded to ChIP analysis.

### Neutral comet assay

Cell suspension at a concentration of 1 × 10^5^ cells/ml was mixed in a 1:1 ratio with Trevigen LMAgarose (#4250-050-02) at 37°C. The mixture was pipetted onto comet slides (Trevigen) pre-coated with a 1% normal melting point agarose (Sigma–Aldrich) base layer. The drop containing the cells was covered with a glass cover slip and incubated at 4°C for 5 min. After incubation, the coverslips were removed, and the slides were immersed in lysis solution [30 mM ethylenediaminetetraacetic acid (EDTA), 0.5% SDS, and 10 mM Tris–HCl, pH 8.0, 500 µg/ml proteinase K] and incubated at 37°C for 1 h. After lysis, the slides were washed three times for 5 min in PBS and incubated in 1 × TBE (Tris–Borate–EDTA buffer) for 20 min at 4°C. Electrophoresis was performed in Trevigen electrophoresis system (#4250-050-ES) for 10 min at 4°C and 1 V/cm in 1 × TBE. The comets were counterstained with SYBR Green for 1 h (1:3000; Thermo Scientific, #S7563) and then visualized using an inverted Nikon Eclipse Ti-E fluorescence microscope with a Nikon Intensilight C-HGFI light source (objective: Nikon Plan Fluor 4/0.13; camera: DS-Qi2). The images of the comets were analyzed using CellProfiler software (version 2.1.1 rev 6c2d896).

### ChIP and ChIP-seq analysis

Living cells were fixed for 15 min with 1% formaldehyde at RT, and crosslinking was quenched by adding 125 mM glycine for 5 min. Cell sorting was performed if needed. Cells were harvested in PBS, and nuclei were prepared by incubation in FL buffer (5 mM PIPES, pH 8.0, 85 mM KCl, 0.5% NP40) supplemented with Protease Inhibitor Cocktail (Bimake) and Phosphatase Inhibitor Cocktail (Bimake) for 30 min on ice. Next, chromatin was sonicated in RIPA buffer (10 mM Tris–HCl, pH 8.0, 140 mM NaCl, 1% Triton X-100, 0.1% sodium deoxycholate, 0.1% SDS) with a VirSonic 100 to an average length of 200–500 bp. Per ChIP reaction, 10–20 µg chromatin was incubated with 2–4 µg antibodies overnight at 4°C. The next day, Protein A/G Magnetic Beads (Thermo Scientific) were added to each sample and incubated for 4 h at 4°C. Immobilized complexes were washed two times for 10 min at 4°C in low salt buffer (20 mM Tris–HCl, pH 8.0, 150 mM NaCl, 2 mM EDTA, 0.1% SDS, 1% Triton X-100) and high salt buffer (20 mM Tris–HCl, pH 8.0, 500 mM NaCl, 2 mM EDTA, 0.1% SDS, 1% Triton X-100). Samples were incubated with RNase A (Thermo Scientific) for 30 min at RT. The DNA was eluted from the beads and de-crosslinked by proteinase K digestion for 4 h at 55°C and subsequent incubation at 65°C for 12 h. Next, DNA was purified using phenol/chloroform extraction and analyzed by quantitative polymerase chain reaction (qPCR). The qPCR primers used for ChIP analysis are listed in Supplementary Table 4. The sequencing libraries were then prepared with NEBNext Ultra II kit according manufacturer’s protocol. Final libraries were PCR amplificated and adapter dimers were cleaned with 1:1 MagPure magnetic beads (Magen Biotechnology). Resulted DNA was resuspended in 30 µl 10 mM Tris–HCl buffer (pH 8.0) and were sequenced on Illumina machine. Chip-seq reads were mapped to the reference human genome hg38 assembly using Bowtie v2.2.3 with the ‘–very-sensitive’ mode. Nonuniquely mapped reads, possible PCR and optical duplicates were filtered out using SAMtools v1.5. The bigWig files with the ratio of RPKM normalized ChIP-seq signal to the input were generated using deepTools v3.4.2 bamCompare function.

### Transcription labeling

For ethynyl uridine (EU) incorporation, the cells were incubated with 100 μM EU (Sigma–Aldrich) for 1 h at 37°C. Then, the cells were washed three times with PBS and fixed in CSK buffer (10 mM PIPES, pH 7.0, 100 mM NaCl, 1.5 mM MgCl_2_, 300 mM sucrose) supplemented with 1% PFA and 2.5% Triton X-100 for 15 min at RT. The samples were then processed using a Click-iT EU Imaging Kit (Life Technologies) according to the manufacturer’s recommendations.

### rDNA fluorescence *in situ* hybridization (FISH)

Cells were fixed with 4% formaldehyde in 1 × PBS for 15 min, then washed three times with 1 × PBS and permeabilized with 0.5% Triton X-100 for 30 min. After permeabilization, cells were washed three times with 1 × PBS. Next, cells were treated with 10 μg/ml RNAse A in 2 × Saline-Sodium Citrate buffer (SSC) at 37°C for at least 45 min, with the following denaturation with 0.1N HCl at RT for 15 min. Then cells were washed 2 times with 2 × SSC and incubated in 50% formamide in 2 × SSC for at least 30 min at RT. Probes for human rDNA (BAC clone RP11-450E20) were received according to the nick-translation protocol using the Alexa 594 NT Labeling Kit and the Atto 488 NT Labeling Kit (Jena Bioscience, Germany). The ethanol-precipitated sample was dissolved in 99.5% formamide (F9037, Sigma–Aldrich) and an equal volume of 4 × SSC/20% dextran sulfate. The sample was denatured at 84°C in a thermomixer, and the cells were denatured in a water bath in a pre-heat 70% formamide/2 × SSC solution at 70°С. For hybridization slides were incubated at 37°C in dark humid chambers for O/N in 37°C which were previously sealed with rubber glue. After hybridization the cells were washed in 50% formamide in 2 × SSC for 5 min 3 times at 45°С, 1 × SSC at 45°С for 5 min 2 times and 1 time at RT.

### Terminal deoxynucleotidyl transferase-mediated labeling and precipitation of DNA lesions (TdT-IP)

Cells were washed with 1 × PBS and pre-extracted twice for 3 min at RT with CSK buffer (10 mM PIPES, pH 7.0, 100 mM NaCl, 300 mM sucrose, 3 mM MgCl₂) supplemented with 0.7% Triton X-100, with RNase A (0.3 mg/ml). After pre-extraction, cells were fixed with 4% formaldehyde in PBS for 15 min at RT. Cells were then washed three times with 1 × PBS and permeabilized with 1% Triton X-100 in 1 × PBS for 10 min, followed by two washes in 1 × PBS. Cells were equilibrated in 1 × Blunting Buffer (100 mM Tris–HCl, pH 7.5, 50 mM NaCl, 10 mM MgCl₂, 0.025% Triton X-100, 5 mM Dithiothreitol (DTT) for 10 min and subsequently incubated in 100 μl of Blunting Buffer supplemented with 4 μl of T4 DNA polymerase (NEB M0203S, 3000 U/μl), 4 μl of T4 polynucleotide kinase (NEB M0201S, 10 000 U/μl), and 10 μl of 10 mM dNTPs. The reaction was carried out for 45 min at RT with gentle agitation. Cells were then washed twice with 1 × TBS (50 mM Tris–HCl, pH 7.5, 150 mM NaCl).

For end-labeling, cells were incubated in TdT labeling buffer containing 50 μl of 5 × TdT buffer (500 mM potassium cacodylate, pH 7.2, 1 mM DTT, 10 mM CoCl₂), 200 μl of 1 × TBS, 5 μl of 10 mM dNTPs, 150 μM biotin-14-dATP (Jena Bioscience), and 400 U of terminal deoxynucleotidyl transferase (TdT; Sigma, #3333566001) in a final volume of 255 μl. Labeling was performed at 37°C for 6 h. Cells were then washed twice with 1 × TBS.

Cells were lysed in 200 μl of lysis buffer (50 mM Tris–HCl, pH 8.0, 10 mM EDTA, 1% SDS) for 15 min at 37°C. Chromatin was fragmented to an average size of 100–500 bp using a VirSonic 100 sonicator. Biotinylated DNA fragments were captured with 50 μl of Dynabeads MyOne Streptavidin C1 magnetic beads (Invitrogen) for 4 h at RT. Beads were washed three times with 450 μl of RIPA ChIP buffer and twice with 200 μl of Tris-EDTA buffer (TE).

DNA was eluted from the beads and decrosslinked by proteinase K digestion for 4 h at 55°C, followed by incubation at 65°C for 12 h. DNA was purified by phenol–chloroform extraction and analyzed by qPCR. Primer sequences used for TdT-IP analysis are listed in Supplementary Tables 5 and 6.

### Proximity ligation assay

Cells were fixed for 15 min with 1% formaldehyde at RT and permeabilized with 1% Triton X-100. Next, cells were subjected to the proximity ligation assay (PLA) using the Duolink^®^  *In Situ* Red Starter Kit Mouse/Rabbit (Sigma–Aldrich, #DUO92101) according to the manufacturer’s instructions. Briefly, cells were blocked, incubated with primary anti-Treacle and anti-TOPBP1 antibodies overnight at 4°C, and then incubated with PLA probes, which are secondary antibodies (anti-mouse-IgG and anti-rabbit-IgG) conjugated to unique oligonucleotides. Samples were then treated with a ligation solution followed by an amplification solution containing polymerase and fluorescently labeled oligonucleotides, allowing rolling-circle amplification and detection of discrete fluorescent dots. Some samples after the PLA protocol were additionally counterstained with Alexa-Fluor-488- or Alexa-Fluor-594-conjugated (Molecular Probes) secondary antibody. The DNA was counterstained with DAPI for 10 min at RT. The samples were mounted using Dako fluorescent mounting medium (Life Technologies) and analyzed using STELLARIS 5 Leica confocal microscope (objectives: HC PL APO 63×/1.40 oil CS2). The images were analyzed using ImageJ software (version 1.44).

### Phosphopeptide enrichment for liquid chromatography–tandem mass spectrometry (LC-MS/MS) analysis

To enrich samples for phosphorylated peptides, we applied the High-Select TiO2 Phosphopeptide Enrichment Kit (Thermo Fisher) according to the manufacturer’s protocol. Briefly, HeLa cells were treated with etoposide or a combination of etoposide and the ATR inhibitor VE-821. 10^7^ cells were washed three times with PBS, lysed in buffer containing 4% SDS, 100 mM Tris–HCl (pH 8.0) with phosphatase inhibitor cocktails 1 and 2 (Sigma–Aldrich), and incubated at 60°C for 30 min. The cell lysates were subjected to sonication on ice (three cycles: 10 s on/off pulses with a 30% amplitude). Disulfide bonds were reduced with DTT (5 mM) for 30 min at RT. Afterward, iodoacetamide was added to a final concentration of 10 mM. The samples were incubated at RT for 20 min in the dark, and the reaction was stopped by adding 5 mM DTT. After the precipitation of proteins using methanol/chloroform, the semi-dry protein pellet was dissolved in 50 µl of 8 M urea, 2 M thiourea, and 10 mM Tris–HCl (pH 8.0). Then, protein amounts were adjusted to 0.5 mg using the Quick Start Bradford protein assay (Bio-Rad), and samples were diluted with ammonium bicarbonate solution to reduce urea concentration to 2 M. Trypsin (Promega) was added at the ratio of 1:100 w/w, and the samples were incubated for 14 h at 37°C. The reaction was stopped by adding formic acid to the final concentration of 5%. The tryptic peptides were desalted using SDB-RPS membrane (Sigma–Aldrich) and vacuum-dried. Then, the phosphoenrichment procedure was performed according to the manufacturer’s protocol. Before LC-MS/MS analysis, samples were redissolved in 5% acetonitrile with 0.1% trifluoroacetate solution and sonicated.

### LC-MS/MS analysis

Proteomic analysis was performed using a Q Exactive HF mass spectrometer. Samples were loaded onto 50-cm columns packed in-house with C18 3 μM Acclaim PepMap 100 (Thermo Fisher), with an Ultimate 3000 Nano LC System (Thermo Fisher) coupled to the MS (Q Exactive HF, Thermo Fisher). Peptides were loaded onto the column thermostatically controlled at 40°C in buffer A (0.2% Formic acid) and eluted with a linear (120 min) gradient of 4%–55% buffer B (0.1% formic acid, 80% acetonitrile) in buffer A at a flow rate of 350 nl/min. Mass spectrometric data were stored during automatic switching between MS1 scans and up to 15 MS/MS scans (topN method). The target value for MS1 scanning was set to 3 × 10^6^ in the range 300–1200 m/z with a maximum ion injection time of 60 ms and a resolution of 60000. The precursor ions were isolated at a window width of 1.4 m/z and a fixed first mass of 100.0 m/z. Precursor ions were fragmented by high-energy dissociation in a C-trap with a normalized collision energy of 28 eV. MS/MS scans were saved with a resolution of 15 000 at 400 m/z and at a value of 1 × 10^5^ for target ions in the range of 200–2000 m/z with a maximum ion injection time of 30 ms.

### Protein identification and spectral counting

Raw LC-MS/MS data from Q Exactive HF mass spectrometer were converted to .mgf peak lists with MSConvert (ProteoWizard Software Foundation). For this procedure, we used the following parameters: “–mgf –filter peakPicking true”. For protein identification, the generated peak lists were searched with MASCOT (version 2.5.1, Matrix Science Ltd., UK) and X! Tandem (ALANINE, 2017.02.01, 2017.02.01, The Global Proteome Machine Organization) search engines against UniProt human protein knowledgebase with the concatenated reverse decoy dataset. The precursor and fragment mass tolerance were set at 20 ppm and 0.04 Da, respectively. Database-searching parameters included the following: tryptic digestion with one possible missed cleavage, static modification for carbamidomethyl (C), and dynamic/flexible modifications for oxidation (M) and phosphorylation (S, T, Y). For X! Tandem, we also selected parameters that allowed a quick check for protein N-terminal residue acetylation, peptide N-terminal glutamine ammonia loss, or peptide N-terminal glutamic acid water loss. Result files were submitted to Scaffold 5 software (version 5.1.0) for validation and meta-analysis. We used the local false discovery rate scoring algorithm with standard experiment-wide protein grouping. For the evaluation of peptide and protein hits, a false discovery rate of 5% was selected for both. False positive identifications were based on reverse database analysis. We also set protein annotation preferences in Scaffold to highlight Swiss-Prot accessions among others in protein groups.

## Results

### The association between TOPBP1 and Treacle in the nucleolus represents a universal response to diverse forms of genotoxic rDNA damage

**Figure 1.**
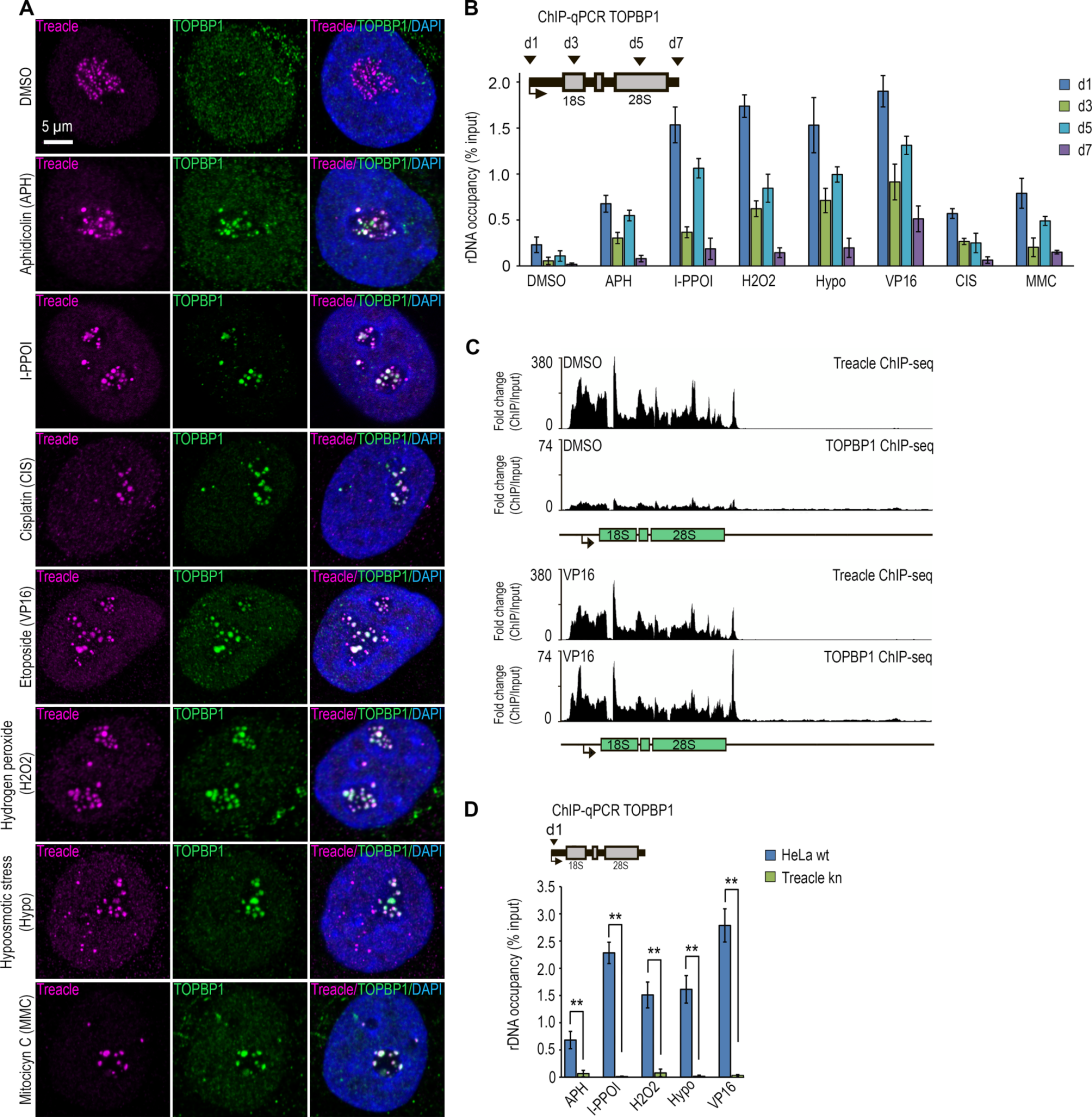
Treacle is required for TOPBP1 recruitment to rDNA in response to genotoxic and nucleolar stress. (**A**) HeLa cells were treated with either 90 μM etoposide (VP16) for 30 min, 1 μM APH for 6 h, 300 μM hydrogen peroxide (H_2_O_2_) for 30 min, 50 μM MMC for 30 min, 50 μM CIS for 30 min. Hypoosmotic stress (hypo) was applied by incubation the cells in 50% DMEM/50% H_2_O (hypoosmotic medium) for 10 min. To induce DNA DSBs using the I-PpoI endonuclease, cells were transiently transfected with a plasmid encoding I-PpoI. After 24 h, nuclear translocation of I-PpoI was triggered by treating the cells with 1 µM 4-OHT for 30 min. Cells were co-immunostained for Treacle (magenta) and TOPBP1 (green) and analyzed by laser scanning confocal microscopy. The DNA was stained with DAPI (blue). (**B**) HeLa cells were processed as described in panel (A). ChIP experiments were performed with antibodies against TOPBP1. ChIP was followed with qPCR using the rDNA amplicons positioned as indicated on the scheme. Data are represented relative to the input. Values are means ± standard deviation (SD) from at least three independent replicates. (**C**) ChIP-seq distributions of Treacle and TOPBP1 on a ribosomal unit of chromosome 21 from control cells (Dimethyl Sulfoxide (DMSO) -treated) and cells treated with VP16 (90 μM, 30 min). (**D**) Intact HeLa (HeLa wt) cells or HeLa cells with CRISPR-Cas9-mediated deletion of the *TCOF1* gene (Treacle kn) were treated with either 90 μM VP16 for 30 min, 1 μM APH for 16 h, 300 μM hydrogen peroxide (H_2_O_2_) for 30 min. Hypoosmotic stress (hypo) was applied by incubation the cells in 50% DMEM/50% H_2_O (hypoosmotic medium) for 10 min. To induce DNA DSBs using the I-PpoI endonuclease, cells were transiently transfected with a plasmid encoding I-PpoI. After 24 h, nuclear translocation of I-PpoI was triggered by treating the cells with 1 µM 4-OHT for 3 h. ChIP experiments were performed with antibodies against TOPBP1. ChIP was followed by qPCR using the d1 primers to the promoter of the rRNA gene (positioned as indicated on the scheme). Data are represented relative to the input. Values are means ± SD from at least three independent replicates. **P <.01, n.s., not significant by unpaired *t*-test.

We previously demonstrated that, under various genotoxic conditions, TOPBP1 relocates to the nucleolus and colocalizes with Treacle in FCs. This phenomenon is specifically observed following inhibition of DNA topoisomerase II by etoposide [7], during APH-induced replication stress [25], and upon stabilization of rDNA R-loops triggered by hypotonic stress [10] (Fig. 1A and Supplementary Fig. S1A). Herein, we show that nucleolar TOPBP1 relocalization also occurs in response to other types of rDNA damage, including DSBs induced by the homing endonuclease I-PPOI, as well as treatment with hydrogen peroxide, CIS, and MMC (Fig. 1A and Supplementary Fig. S1A). Under all of these conditions, TOPBP1 robustly accumulated in FCs and colocalized with Treacle during the first 30–60 min of treatment (Fig. 1A and Supplementary Fig. S1A). Upon prolonged stress exposure (3 h), TOPBP1 and Treacle translocated to the periphery of the nucleolus where they formed characteristic “caps” indicative of transcriptional repression (Supplementary Fig. S1B). A notable exception occurred following APH treatment, in that TOPBP1 accumulated in FCs only after 6 h of incubation, after which it formed large intranucleolar clusters without initiating cap formation (Fig. 1A). Nevertheless, ChIP-qPCR analysis revealed that, under all conditions examined, TOPBP1 was significantly enriched in the coding region of the ribosomal gene (Fig. 1B). Furthermore, ChIP-seq profiling using antibodies against TOPBP1 and Treacle in etoposide-treated cells showed nearly identical rDNA-binding profiles (Fig. 1C).

To exclude the possibility that this response is HeLa cell-specific, we examined TOPBP1 recruitment to the ribosomal gene promoter in multiple cell types, including human primary skin fibroblasts, under various DNA-damaging conditions. TOPBP1 recruitment to rDNA was largely conserved across cell types, indicating a general feature of the n-DDR (Supplementary Fig. S1C), although its magnitude varied in a cell type-dependent manner. Notably, human fibroblasts, HEK293, and HCT116 cells showed little or no response to etoposide or MMC, whereas hypotonic stress and hydrogen peroxide consistently induced robust TOPBP1 accumulation at the rDNA promoter in all cell types examined (Supplementary Fig. S1C).

To validate the role of Treacle in TOPBP1 recruitment, we generated a Treacle-knockout HeLa cell line (Treacle-kn) by targeting the *TCOF1* gene (Supplementary Fig. S1D). In these cells, TOPBP1 failed to re-localize to the nucleolus or occupy rDNA in response to any genotoxic stressor, unlike in wild-type (WT) cells (Fig. 1D and Supplementary Fig. S1E).

Collectively, these findings indicate that (i) TOPBP1 nucleolar relocalization and its association with rDNA and FCs constitute an early and broadly conserved response to diverse forms of rDNA damage, (ii) the efficiency of this response depends on the nature of the nucleolar stress and cellular context, and (iii) Treacle is an essential and nonredundant determinant of TOPBP1 recruitment to rDNA across all examined conditions.

### TOPBP1 uses treacle as a condensation platform

**Figure 2.**
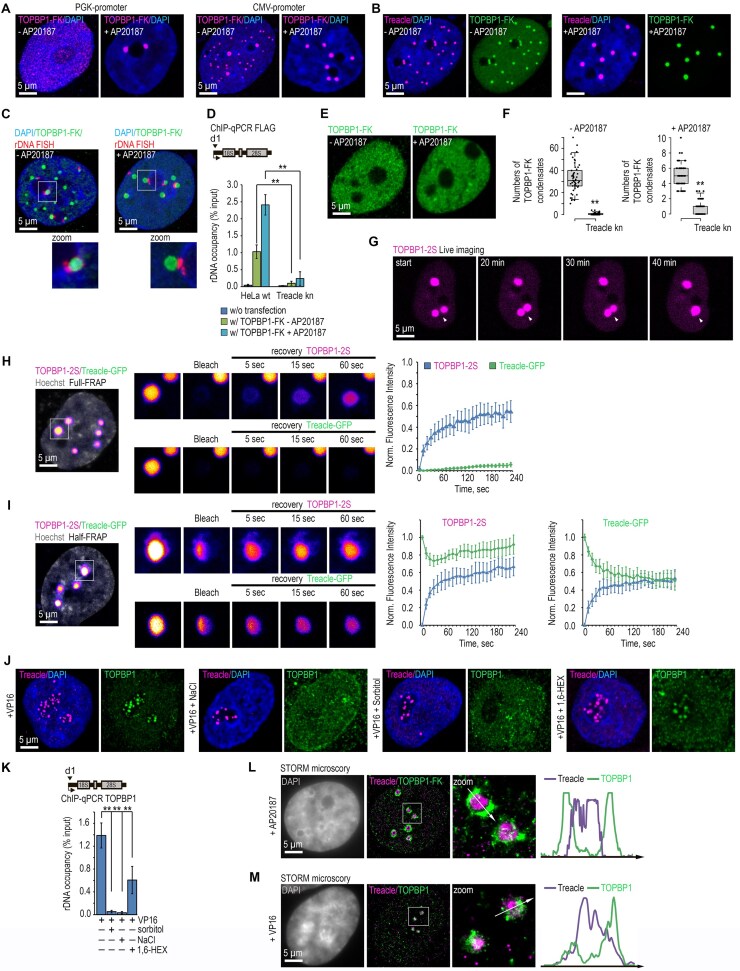
TOPBP1 undergoes phase separation at the surface of Treacle condensates. (**A**) HeLa cells were transfected with TOPBP1-FLAG–FKBP12F36V (TOPBP1-FK; magenta), expressed either under the control of the weak PGK promoter or the strong CMV promoter, at a dose of 500 ng plasmid per 2 × 10^5^ cells. To induce TOPBP1 oligomerization, cells were treated with 100 nM AP20187 for 24 h. Cells were fixed 24 h after transfection, immunostained with anti-FLAG antibodies (magenta), and analyzed by laser scanning confocal microscopy. DNA was counterstained with DAPI (blue). (**B**) HeLa cells were transfected with TOPBP1-FLAG–FKBP12F36V (TOPBP1-FK), expressed under the control of the strong CMV promoter. To induce TOPBP1 oligomerization, cells were treated with 100 nM AP20187 for 24 h. The cells were fixed 24 h after transfection, co-immunostained for FLAG (green) and Treacle (magenta) antibodies and analyzed by laser scanning confocal microscopy. DNA was stained with DAPI (blue). (С) HeLa cells were processed as described in panel (B). Cells were fixed 24 after transfection and stained for rDNA (rDNA FISH). DNA was stained with DAPI (blue). Cells were analyzed by laser scanning confocal microscopy. (**D**) HeLa wt or HeLa Treacle kn were processed as described in panel (B). Untransfected cells were used as controls. ChIP experiments were performed with antibodies against FLAG. ChIP was followed by qPCR using the d1 primers to the promoter of the rRNA gene. Data are represented relative to the input. Values are means ± SD from at least three independent replicates. **P <.01, n.s., not significant by unpaired *t*-test. (**E**) HeLa cells with CRISPR-Cas9-mediated deletion of the *TCOF1* gene (Treacle kn) were transiently transfected with plasmid constructs encoding TOPBP1-FLAF-FKBP12F36V (TOPBP1-FK; green). For induction of TOPBP1 oligomerization, transfected cells were incubated with 100 nM AP20187 for 24 h. The cells were fixed 24 h after transfection, immunostained for FLAG antibodies (green) and analyzed by laser scanning confocal microscopy. (**F**) HeLa wt or HeLa Treacle kn were processed as described in panel (B). The number of TOPBP1-FK condensates per cell was measured (*n* > 50). **P <.01, n.s., not significant by unpaired *t*-test. (**G**) Twenty-four hours after transfection with TOPBP1-2S, HeLa cells were live time-lapse imaged. Representative images of cells with TOPBP1-2S condensates fusion events are shown. (**H**) HeLa cells were transiently co-transfected with Treacle-GFP and TOPBP1-2S. Twenty-four hours after transfection full-FRAP analysis of Treacle/TOPBP1 (TT) condensates was performed. One whole condensate was bleached, and fluorescence recovery for Treacle-GFP and TOPBP1-2S was monitored. Representative time-lapse images of the photobleached condensates are shown (magnified images). DNA was stained with Hoechst 33 342 (gray). Graphs illustrate the quantification of Treacle-GFP and TOPBP1-2S the full-FRAP analysis. Each trace represents an average of measurements for at least twenty TT condensates; error bars represent SD. (**I**) HeLa cells were transiently co-transfected with Treacle-GFP and TOPBP1-2S. Twenty-four hours after transfection half-FRAP analysis of Treacle/TOPBP1 (TT) condensates was performed. Half of the one condensate was bleached, and fluorescence recovery for Treacle-GFP and TOPBP1-2S was monitored. Representative time-lapse images of the photobleached condensates are shown (magnified images). DNA was stained with Hoechst 33 342 (gray). Graphs illustrate the quantification of Treacle-GFP and TOPBP1-2S the half-FRAP analysis. Half-FRAP curves represent the normalized intensity in the bleached half (blue) and the nonbleached half (green). Each trace represents an average of measurements for at least 20 TT condensates; error bars represent SD. (**J**) Cells were treated with 90 μM VP16 for 30 min, followed by incubation with either 300 mM sodium chloride for 30 min, 300 mM sorbitol for 30 min, or 5% 1,6-hexanediol for 10 min. The cells were fixed, co-immunostained for TOPBP1 (green) and Treacle (magenta) antibodies and analyzed laser scanning confocal microscopy. DNA was stained with DAPI (blue). (**K**) HeLa cells were processed as described in panel (J). ChIP experiments were performed with antibodies against TOPBP1. ChIP was followed by qPCR using the d1 primers to the promoter of the rRNA gene. Data are represented relative to the input. Values are means ± SD from at least three independent replicates. **P <.01, n.s., not significant by unpaired *t*-test. (**L**) HeLa cells were transfected with TOPBP1-FLAF-FKBP12F36V (TOPBP1-FK; green) at a quantity of 500 ng plasmids per 2 × 10^5^ cells. For induction of TOPBP1 oligomerization, transfected cells were incubated with 100 nM AP20187 for 24 h. The cells were fixed 24 h after transfection, immunostained for FLAG (green) and Treacle (magenta) antibodies and analyzed by STORM microscopy. DNA was stained with DAPI (gray). Co-localization analysis was performed on the merged images. Graphs illustrate quantification in arbitrary units of fluorescence distribution along the lines shown in the figures. (**M**) Cells were treated with 90 μM VP16 for 30 min, fixed, immunostained for TOPBP1 (green) and Treacle (magenta) antibodies and analyzed by STORM microscopy. DNA was stained with DAPI (gray). Co-localization analysis was performed on the merged images. Graphs illustrate quantification in arbitrary units of fluorescence distribution along the lines shown in the figures.

Recent work has demonstrated that TOPBP1 is capable of forming biomolecular condensates via LLPS [29, 30]. Importantly, we have previously shown that Treacle itself also undergoes LLPS both in cells and *in vitro*, forming dynamic liquid-like condensates that are essential for FC assembly, efficient rRNA transcription and processing, and rDNA repair, thereby providing a potential nucleolar scaffold for client proteins [7, 32]. These observations raised the possibility that Treacle could serve as a platform to nucleate or facilitate TOPBP1 condensation within the nucleolus. We therefore next sought to determine whether TOPBP1 condensation operates in the context of the nucleolar stress response. To address this question, we first established a convenient cellular model to experimentally manipulate and monitor TOPBP1 condensation. To this end, we generated several TOPBP1 constructs fused either to the red fluorescent protein Katushka2S (TOPBP1-2S) or to FLAG and FKBPF36V (TOPBP1-FK), enabling inducible oligomerization upon treatment with the rapamycin analog AP20187 [31]. All constructs were expressed either under the control of the weak PGK promoter or the strong сytomegalovirus (CMV) promoter. When TOPBP1-FK was expressed from the weak PGK promoter, TOPBP1 was diffusely distributed throughout the nucleus and no condensates were detected (Fig. 2A). However, AP20187-induced oligomerization triggered the formation of discrete, single nuclear TOPBP1 condensates (Fig. 2A). In contrast, expression of TOPBP1-FK from the strong CMV promoter resulted in spontaneous formation of numerous small nuclear condensates even in the absence of induced oligomerization (Fig. 2A). Upon AP20187 treatment, these condensates markedly increased in size, consistent with enhanced multivalent interactions (Fig. 2A). Notably, TOPBP1-2S formed condensates under both weak and strong expression conditions (Supplementary Fig. S2A); however, condensates were more numerous, larger, and brighter when TOPBP1-2S was expressed from the CMV promoter (Supplementary Fig. S2A). This behavior suggests that the Katushka2S tag itself facilitates TOPBP1 oligomerization, consistent with the well-established propensity of Katushka-derived fluorescent proteins to multimerize.

Together, these results demonstrate that TOPBP1 is intrinsically capable of forming condensates in cells, and that this capacity is strongly dependent on protein abundance and multimerization potential. However, it remains unclear whether TOPBP1 condensation occurs autonomously or instead requires co-condensation with binding partners. Treacle, like TOPBP1, is an intrinsically disordered nucleolar protein (Supplementary Fig. S2B), and we previously showed that it can undergo condensation both *in vitro* and *in vivo* [7, 32]. Moreover, we demonstrated that the condensation capacity of Treacle is essential for TOPBP1 recruitment to rDNA upon rDNA damage [7]. We therefore hypothesized that TOPBP1 may form condensates through co-condensation with Treacle. Therefore next, we examined the role of endogenous Treacle in the formation of artificial TOPBP1 condensates.

Immunofluorescence analysis revealed that all types of TOPBP1 condensates colocalized with endogenous Treacle (Fig. 2B, and Supplementary Fig. S2C and D). Moreover, quantification by immuno-FISH and ChIP-qPCR showed that all cells containing TOPBP1 condensates (∼100%) displayed association of at least a subset of these structures with rDNA (Fig. 2D and E). Unexpectedly, in Treacle-kn cells, the formation of both TOPBP1-2S and TOPBP1-FK condensates was nearly abolished, and no associations with rDNA were observed (Fig. 2D–F, and Supplementary Fig. S2E and F). These findings suggest that TOPBP1 does not condense in cells independently, but instead co-condenses with Treacle to form heterotypic condensates. We refer to these structures as Treacle-dependent condensates or TT condensates (Treacle-TOPBP1 condensates).

To characterize the biophysical properties of TT condensates, we investigated their dynamics. Specifically, phase-separated condensates typically exhibit fusion behavior and dynamic molecular exchange [33]. Indeed, live-cell imaging showed that TOPBP1-2S condensates readily fused, indicating liquid-like properties (Fig. 2G). To assess molecular mobility, we performed both full- and half-FRAP experiments [34]. Full-FRAP measures exchange with the surrounding environment, while half-FRAP reflects internal redistribution. We co-expressed TOPBP1-2S and Treacle-GFP in HeLa cells, where they colocalized in TT condensates. Full-FRAP revealed rapid TOPBP1 fluorescence recovery but little recovery of Treacle (Fig. 2H). In half-FRAP experiments, Treacle exhibited internal mobility without external exchange, while TOPBP1 rapidly recovered without loss in the unbleached half, indicating molecular exchange with the surrounding nucleoplasm (Fig. 2I). Similar dynamics were observed for TOPBP1-2S expressed alone, ruling out artifacts caused by Treacle overexpression (Supplementary Fig. S2G). Together, these data suggest that Treacle is internally dynamic but forms a relatively stable phase, whereas TOPBP1 remains highly mobile and exchanges with the surrounding environment—behavior more consistent with interaction at internally clustered binding sites [35] than classical LLPS.

This observed molecular dynamics asymmetry led us to hypothesize that Treacle and TOPBP1 form distinct subphases within TT condensates with differing physicochemical properties. To test this hypothesis, we treated cells with sorbitol (to disrupt electrostatic interactions) or 1,6-hexanediol (to disrupt hydrophobic interactions) and assessed their sensitivity to the disruption of weak molecular interactions. In both cases, TOPBP1 rapidly dispersed from TT condensates, whereas Treacle remained stable (Supplementary Fig. S2H and I). Similar results were observed for endogenous TOPBP1 in FCs and on rDNA in response to etoposide treatment (Fig. 2J and K). These findings indicate that although TOPBP1 co-condenses with Treacle, the two proteins form physically distinct phases with differing stability and interaction modes.

To further characterize the spatial organization of these subphases, we employed STORM super-resolution microscopy and PLA. These assays revealed that Treacle and TOPBP1 do not fully mix: Treacle forms the core of the condensate, while TOPBP1 is localized to its periphery (Fig. 2L and Supplementary Fig. S2J). A similar spatial arrangement was observed in FCs upon rDNA damage (Fig. 2M). Collectively, our findings demonstrate that TOPBP1 condenses in cells by interacting with Treacle, but occupies a distinct, spatially and physically separate phase at the surface of TT condensates or FCs.

### Cooperative phosphorylation of Treacle at Ser1190/1191 and Ser1199 by CK2 and ATR kinases initiates TOPBP1 condensation

Previous work has demonstrated that phosphorylation of serines 1227, 1228, and 1236 in the C-terminal region of Treacle is required for its interaction with TOPBP1 in response to I-PPO1-induced rDNA damage [23]. Therefore, we hypothesized that these phospho-sites act as “seeds” for TOPBP1 recruitment and its condensation initiation. To test this hypothesis, we analyzed the phosphoproteome of etoposide-treated HeLa cells. First, we determined that these cells predominantly express Treacle isoform d, in which Ser1227, Ser1228, and Ser1236 correspond to Ser1190, Ser1191, and Ser1199, respectively. Second, mass spectrometry analysis revealed that Ser1191 and Ser1199 undergo marked phosphorylation in response to etoposide treatment (Supplementary Fig. S3A).

**Figure 3.**
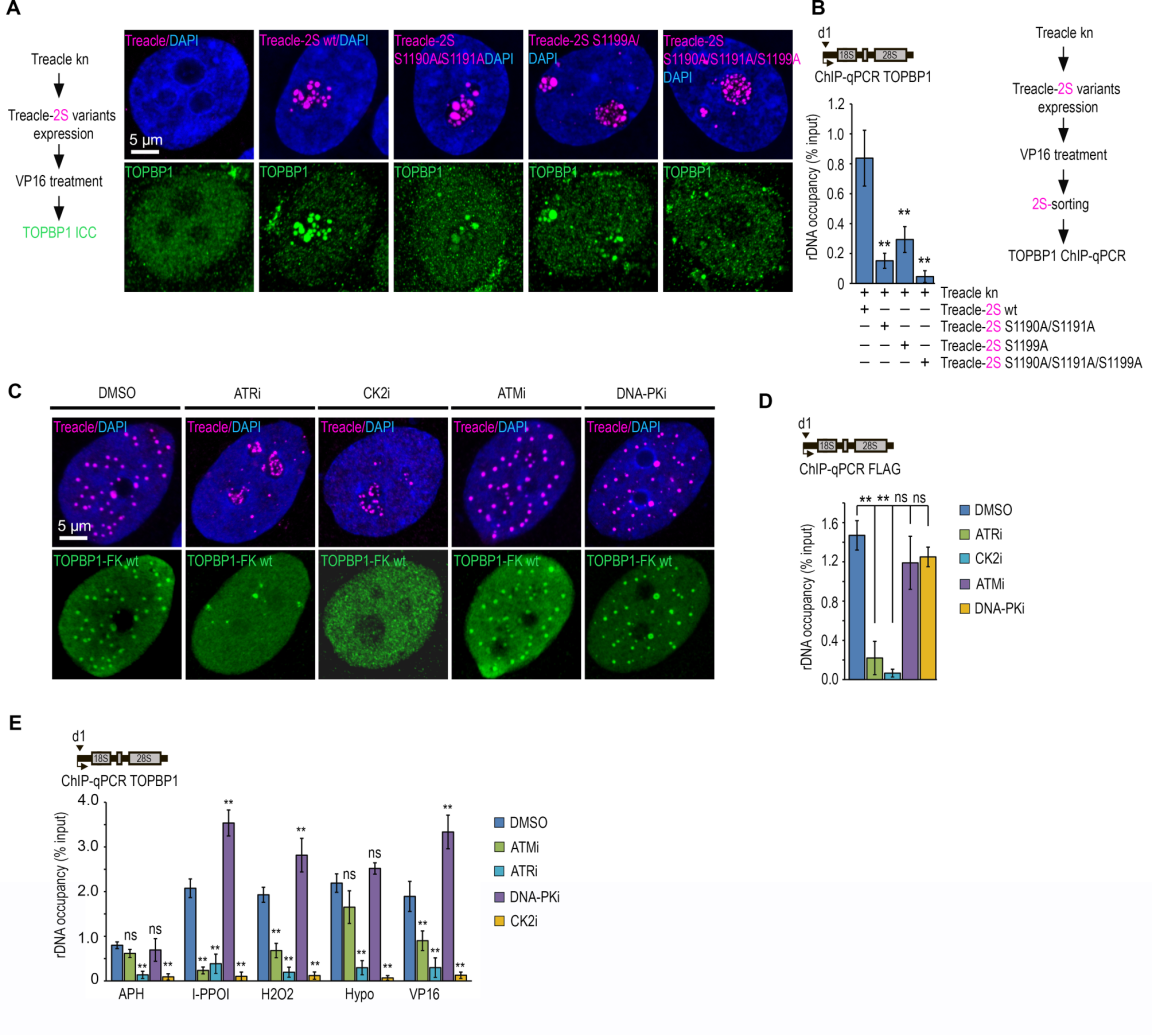
Condensation of TOPBP1 requires ATR/CK2-dependent phosphorylation of Treacle. (**A**) HeLa cells with CRISPR-Cas9-mediated deletion of the TCOF1 gene (Treacle kn) were transiently transfected with plasmid constructs encoding either Treacle-2S wt, Treacle-2S S1190A/S1191A, Treacle-2S S1199A, or Treacle-2S S1190A/S1191A/S1199A. Twenty-four hours after transfection, cells were treated with VP16 (90 μM for 30 min), fixed and stained for TOPBP1 (green) and analyzed by laser scanning confocal microscopy. The DNA was stained with DAPI (blue). (**B**) HeLa cells with CRISPR-Cas9-mediated deletion of the TCOF1 gene (Treacle kn) were transiently transfected with plasmid constructs encoding either Treacle-2S wt, Treacle-2S S1190A/S1191A, Treacle-2S S1199A or Treacle-2S S1190A/S1191A/S1199A. Twenty-four hours after transfection, cells were treated with VP16 (90 μM for 30 min), fixed and subjected to cell sorting in the fluorescent analysis mode to obtain 2S-positive populations. At least 2 × 10^6^ sorted cells were used for ChIP with TOPBP1 antibodies. ChIP was followed by qPCR using the d1 primers to the promoter of the rRNA gene (positioned as indicated on the scheme). Data are represented relative to the input. Values are means ± SD from at least three independent replicates. **P <.01, n.s., not significant by unpaired *t*-test. (**C**) HeLa cells were transfected with TOPBP1-FLAF-FKBP12F36V (TOPBP1-FK; green). Twenty-four hours after transfection, cells were treated with either 15 µM VE-821 (ATRi) for 3 h, 30 µM CX-4945 (CK2i) for 3 h, 20 µM NU7441 (DNAPKi) for 3 h or 20 µM KU55933 (ATMi) for 3 h. Next cells were immunostained for FLAG (green) and Treacle (magenta) antibodies and analyzed by laser scanning confocal microscopy. DNA was stained with DAPI (blue). (**D**) HeLa cells were processed as described in panel (C). Cells were used for ChIP with FLAG antibodies. ChIP was followed by qPCR using the d1 primers to the promoter of the rRNA gene (positioned as indicated on the scheme). Data are represented relative to the input. Values are means ± SD from at least three independent replicates. **P <.01, n.s., not significant by unpaired *t*-test. (**E**) HeLa cells were preincubated with either 15 µM VE-821 (ATRi) for 3 h, 30 µM CX-4945 (CK2i) for 3 h, 20 µM NU7441 (DNAPKi) for 3 h or 20 µM KU55933 (ATMi) for 3 h and then treated with either 90 μM VP16 for 30 min, 300 μM hydrogen peroxide (H_2_O_2_) for 30 min or incubated with hypoosmotic medium for 10 min. To investigate the role of kinases in replication stress, cells were treated with APH for 16 h, followed by incubation with the respective kinase inhibitors as previously described. To assess the involvement of kinases in I-PpoI-induced rDNA damage, cells were transiently transfected with a plasmid encoding I-PpoI. After 24 h, kinase inhibitors were applied as above, followed by induction of I-PpoI nuclear translocation via treatment with 1 µM 4-OHT for 30 min. ChIP experiments were performed with antibodies against TOPBP1. ChIP was followed by qPCR using the d1 primers to the promoter of the rRNA gene (positioned as indicated on the scheme). Data are represented relative to the input. Values are means ± SD from at least three independent replicates. **P <.01, n.s., not significant by unpaired *t*-test.

We next assessed the functional relevance of these phospho-sites in initiating Treacle-dependent condensation of TOPBP1. To this end, we expressed mutant variants of Treacle-2S in Treacle-kn cells, in which the relevant serines were substituted with alanines: Treacle-2S S1190A/S1191A, Treacle-2S S1199A, and the triple mutant Treacle-2S S1190A/S1191A/S1199A. Importantly, all Treacle phospho-mutants formed FCs and occupied rDNA with efficiencies comparable to WT Treacle when expressed in Treacle-kn cells, demonstrating that these phosphorylation sites are dispensable for Treacle condensation and nucleolar targeting per se (Fig. 3A and Supplementary Fig. S3B). Using immunofluorescence staining and ChIP-qPCR, we evaluated the capacity of these mutants to induce TOPBP1 condensation in the FC and its binding to rDNA following etoposide treatment. In contrast to WT Treacle-2S, all three mutants elicited pronounced defects: the single and double mutants only partially restored TOPBP1 condensation and rDNA occupancy, whereas the triple mutant completely failed to do so (Fig. 3A and B). These data indicate that both Ser1191 and Ser1199 contribute to interactions with TOPBP1 and likely act cooperatively.

Ser1199 is located within an SQ motif, a canonical recognition site for PIKK-family kinases (e.g. ATM, ATR, DNA-PK), whereas Ser1191 lies within an SSEDVV sequence, which resembles a potential CK2 consensus motif (Supplementary Fig. S3A). Therefore, we hypothesized that the activity of one or more of these kinases is required to initiate Treacle-mediated TOPBP1 condensation. To test this hypothesis, we examined TOPBP1 condensation in the presence of ATM, ATR, DNA-PK, or CK2 chemical inhibitors (ATMi [KU-55933), ATRi (VE-821), DNA-PKi (NU-7441), and CK2i (CX-4945), respectively]. We found that both ATRi and CK2i broadly disrupted the formation of TT condensates as well as Treacle-dependent TOPBP1 condensation in the FC under genotoxic stress (Fig. 3C–E and Supplementary Fig. S3C). These effects occurred in the absence of enforced TOPBP1 oligomerization and were consistent across all types of genotoxic stimuli examined. In contrast, inhibition of ATM or DNA-PK did not affect model condensate formation or TOPBP1 localization to rDNA (Fig. 3C–E and Supplementary Fig. S3C). However, under conditions that preferentially generate DNA DSBs, such as I-PPO1 induction, hydrogen peroxide treatment, and exposure to VP16, ATMi significantly impaired TOPBP1 recruitment to rDNA (Fig. 3E). Conversely, DNA-PKi did not inhibit, and in some cases even enhanced, TOPBP1 localization to rDNA, potentially due to NHEJ inhibition and accumulation of unrepaired DSBs (Fig. 3E and Supplementary Fig. S3C). Thus, our data indicate that Ser1199 phosphorylation, which is required for TOPBP1 recruitment, is predominantly mediated by ATR during the formation of model condensates. Conversely, under genotoxic stress, both ATR and ATM can contribute. In contrast, phosphorylation of Ser1191 is strictly dependent on CK2 activity under all conditions examined.

To validate these findings, we performed phosphoproteomic analysis of HeLa cells treated with etoposide in the presence of ATMi or ATRi. Under both conditions, we observed a substantial reduction in Ser1199 phosphorylation, but not that of Ser1191 (Supplementary Fig. S3A). These results confirm that Ser1199 is regulated by ATM and/or ATR, whereas Ser1191 is predominantly targeted by CK2.

Collectively, our findings demonstrate that initiation of Treacle-dependent TOPBP1 condensation requires Treacle Ser1191 and Ser1199 phosphorylation, events regulated by distinct kinases. This kinase-specific regulation provides both spatial and signal-specific control over TOPBP1 phase separation in response to rDNA damage. Therefore, we next pursued the molecular mechanisms by which TOPBP1 recognizes these phospho-sites.

### TOPBP1 engages with treacle phospho-Ser1190/1191 and Ser1199 via its BRCT2 and BRCT5 domains, respectively

We [25] and others [23] have previously demonstrated that TOPBP1 interacts with Treacle through two independent regions involving the BRCT2 and BRCT5 domains of TOPBP1 (Supplementary Fig. S4A). Given that BRCT domains function as canonical phospho-recognition modules [36–39], we hypothesized that TOPBP1 binds Treacle via simultaneous, cooperative engagement of BRCT2 and BRCT5 with distinct Treacle phospho-serine motifs. To test this hypothesis, we used our previously established system based on inducible FKBP-mediated oligomerization of TOPBP1, reasoning that enforced oligomerization may compensate for the loss of one of the two phospho-dependent binding interfaces with Treacle.

**Figure 4.**
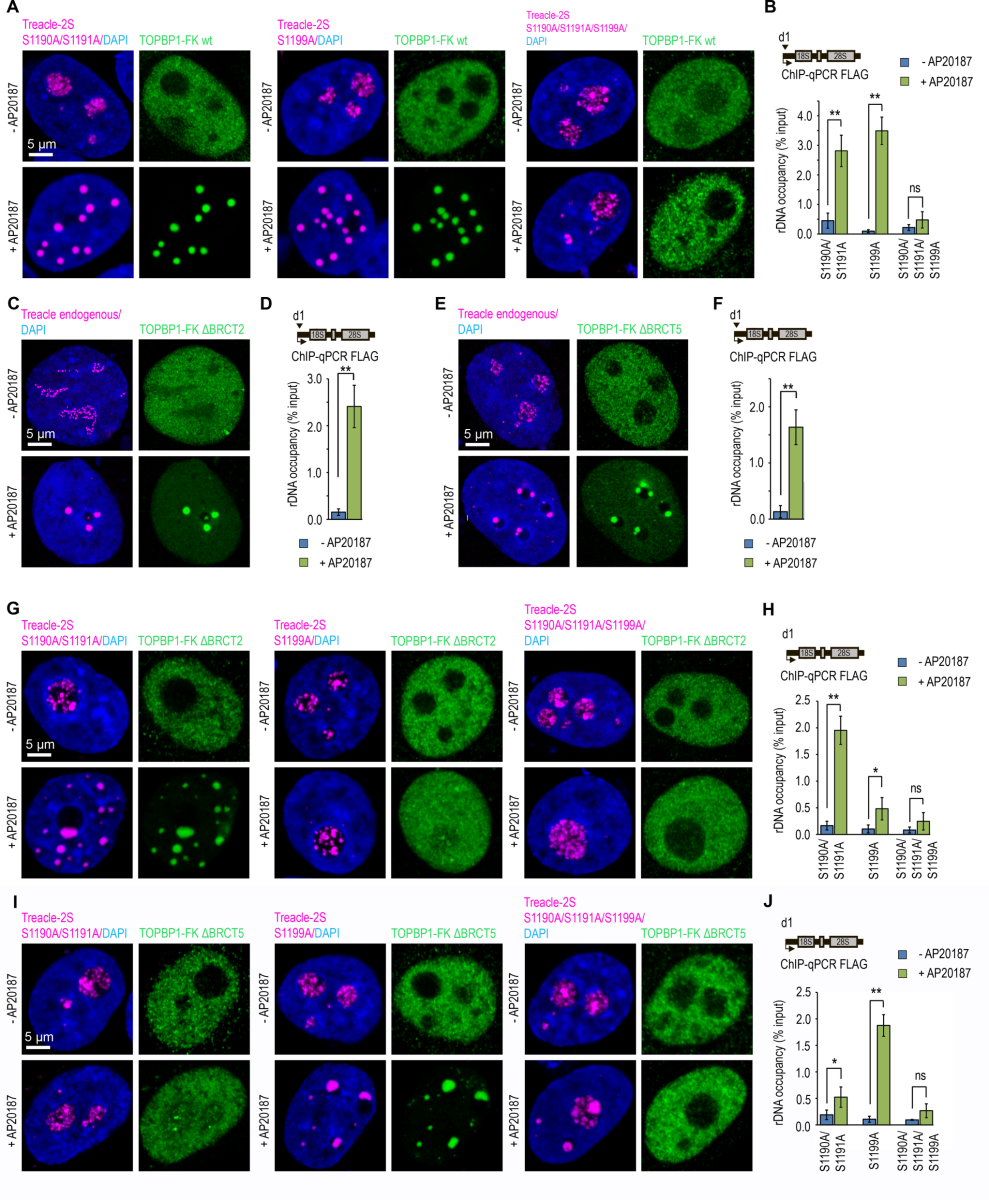
BRCT2 and BRCT5 domains of TOPBP1 engage Treacle phospho-Ser1190/1191 and Ser1199 to drive TOPBP1 condensation. (**A**) Treacle kn cells were transiently co-transfected with combination plasmid constructs encoding TOPBP1-FK wt and either Treacle-2S S1190A/S1191A (magenta), Treacle-2S S1199A (magenta), or Treacle-2S S1190A/S1191A/S1199A (magenta). For induction of TOPBP1 oligomerization, transfected cells were incubated with 100 nM AP20187 for 24 h. After 24 h cells were fixed and immunostained for FLAG (green) antibodies and analyzed by laser scanning confocal microscopy. DNA was stained with DAPI (blue). (**B**) HeLa cells were processed as described in panel (A). ChIP experiments were performed with antibodies against FLAG. ChIP was followed by qPCR using the d1 primers to the promoter of the rRNA gene. Data are represented relative to the input. Values are means ± SD from at least three independent replicates. **P <.01, n.s., not significant by unpaired *t*-test. (**C**) HeLa TOPBP1 kn cells were transfected with TOPBP1-FK ΔBRCT0-2. For induction of TOPBP1 oligomerization, transfected cells were incubated with 100 nM AP20187 for 24 h. After 24 h cells fixed and co-immunostained for FLAG (green) and Treacle (magenta) antibodies and analyzed by laser scanning confocal microscopy. DNA was stained with DAPI (blue). (**D**) HeLa cells were processed as described in panel (C). ChIP experiments were performed with antibodies against FLAG. ChIP was followed by qPCR using the d1 primers to the promoter of the rRNA gene. Data are represented relative to the input. Values are means ± SD from at least three independent replicates. **P <.01, n.s., not significant by unpaired *t*-test. (**E**) HeLa TOPBP1 kn cells were transfected with TOPBP1-FK ΔBRCT5. For induction of TOPBP1 oligomerization, transfected cells were incubated with 100 nM AP20187 for 24 h. After 24 h cells were fixed and co-immunostained for FLAG (green) and Treacle (magenta) antibodies and analyzed by laser scanning confocal microscopy. DNA was stained with DAPI (blue). (**F**) HeLa cells were processed as described in panel (E). ChIP experiments were performed with antibodies against FLAG. ChIP was followed by qPCR using the d1 primers to the promoter of the rRNA gene. Data are represented relative to the input. Values are means ± SD from at least three independent replicates. **P <.01, n.s., not significant by unpaired *t*-test. (**G**) Treacle kn cells were transiently co-transfected with combination plasmid constructs encoding TOPBP1-FK ΔBRCT0-2 and either Treacle-2S S1190A/S1191A (magenta), Treacle-2S S1199A (magenta), or Treacle-2S S1190A/S1191A/S1199A (magenta). For induction of TOPBP1 oligomerization, transfected cells were incubated with 100 nM AP20187 for 24 h. After 24 h cells were fixed and immunostained for FLAG (green) antibodies and analyzed by laser scanning confocal microscopy. DNA was stained with DAPI (blue). (**H**) HeLa cells were processed as described in panel (G). ChIP experiments were performed with antibodies against FLAG. ChIP was followed by qPCR using the d1 primers to the promoter of the rRNA gene. Data are represented relative to the input. Values are means ± SD from at least three independent replicates. **P <.01, n.s., not significant by unpaired *t*-test. (**I**) Treacle kn cells were transiently co-transfected with combination plasmid constructs encoding TOPBP1-FK ΔBRCT5 and either Treacle-2S S1190A/S1191A (magenta), Treacle-2S S1199A (magenta), or Treacle-2S S1190A/S1191A/S1199A (magenta). For induction of TOPBP1 oligomerization, transfected cells were incubated with 100 nM AP20187 for 24 h. After 24 h cells were fixed and immunostained for FLAG (green) antibodies and analyzed by laser scanning confocal microscopy. DNA was stained with DAPI (blue). (**J**) HeLa cells were processed as described in panel (I). ChIP experiments were performed with antibodies against FLAG. ChIP was followed by qPCR using the d1 primers to the promoter of the rRNA gene. Data are represented relative to the input. Values are means ± SD from at least three independent replicates. **P <.01, n.s., not significant by unpaired *t*-test.

We first assessed whether enforced TOPBP1 oligomerization could rescue its binding to phospho-site-deficient Treacle mutants. To this end, we co-expressed TOPBP1-FK in Treacle-kn cells together with each of the following Treacle-2S variants: the Treacle-2S S1190A/S1191A double mutant, the Treacle-2S S1199A single mutant, and the Treacle-2S S1190A/S1191A/S1199A triple mutant. TT condensate formation and TOPBP1 binding to rDNA were evaluated before and after FKBP-mediated oligomerization. As expected, no condensates formed under any condition prior to oligomerization (Fig. 4A and B). However, following induction, condensate formation and rDNA occupancy were restored in cells expressing Treacle-2S S1190A/S1191A or Treacle-2S S1199A, but not in those expressing the triple mutant (Fig. 4A and B). These findings indicate that forced oligomerization can rescue Treacle-dependent condensation of TOPBP1 when only one phosphorylated serine residue remains accessible. We propose that enforced oligomerization of TOPBP1 does not introduce an alternative binding interface but instead increases the effective local concentration and avidity of the remaining phospho-dependent interaction. This would allow TOPBP1 to maintain productive engagement with Treacle when only a single phospho-site is available. In contrast, simultaneous loss of both phospho-dependent interactions cannot be compensated by oligomerization, underscoring the requirement for at least one intact BRCT–phosphoserine interface to nucleate TOPBP1 condensation.

We next evaluated whether oligomerization could also compensate for the absence of one of the TOPBP1 BRCT domains. To do so, we expressed TOPBP1-FK deletion mutants lacking either BRCT2 or BRCT5 (TOPBP1-FK ΔBRCT2 and TOPBP1-FK ΔBRCT5, respectively) in *TOPBP1*-knockdown HeLa cells (Supplementary Fig. S4B). Prior to oligomerization, neither mutant was able to form TT condensates with endogenous Treacle (Fig. 4C–F). However, induced oligomerization rescued the ability of both variants to form condensates and bind rDNA (Fig. 4C–F), supporting the notion that TOPBP1 oligomerization can functionally compensate for the loss of one phospho-binding interface.

We then used this system to map specific interactions between individual Treacle phospho-sites and TOPBP1 BRCT domains by co-expressing TOPBP1-FK ΔBRCT2 or ΔBRCT5 in Treacle-kn cells with various Treacle-2S phospho-mutants: S1190A/S1191A, S1199A, or S1190A/S1191A/S1199A. Upon induction of oligomerization, condensates formed exclusively under two conditions: Treacle-2S S1190A/S1191A with TOPBP1-FK ΔBRCT2, and Treacle-2S S1199A with TOPBP1-FK ΔBRCT5 (Fig. 4G–J and Supplementary Fig. S4G). These results indicate that Ser1199 is recognized by BRCT5, whereas BRCT2 binds Ser1190/1191.

To further validate the specificity of these interactions, we analyzed the sensitivity of the resulting condensates to CK2i and ATRi. To this end, we induced oligomerization of TOPBP1-FK ΔBRCT2 or TOPBP1-FK ΔBRCT5 in *TOPBP1*-knockdown HeLa cells treated with ATRi or CK2i. BRCT5-dependent condensate formation (i.e. in cells lacking BRCT2) was sensitive to ATRi but not CK2i (Supplementary Fig. S4C and D). Conversely, BRCT2-dependent condensates were sensitive to CK2i but resistant to ATRi (Supplementary Fig. S4E and F). Collectively, these results confirm that the BRCT5 domain of TOPBP1 binds to Treacle in an ATR-dependent manner via phosphorylated Ser1199, while BRCT2 engages Ser1190/1191 phosphorylated by CK2. These site-specific interactions enable bivalent phospho-site recognition, establishing a nucleation platform for subsequent TOPBP1 condensation.

### Treacle-dependent condensation of TOPBP1 in the FC is regulated by ATR kinase activity via the AAD and BRCT7/8 domain of TOPBP1

Previous studies have shown that the C-terminal fragment of TOPBP1, which contains ATR activation (AAD) and BRCT7/8 domains, can self-associate and undergo phase separation *in vitro* [29, 30]. A positive feedback loop between the phase separation of this fragment and ATR activation has also been identified. Therefore, we hypothesized that a similar mechanism operates during Treacle-dependent TOPBP1 condensation.

**Figure 5.**
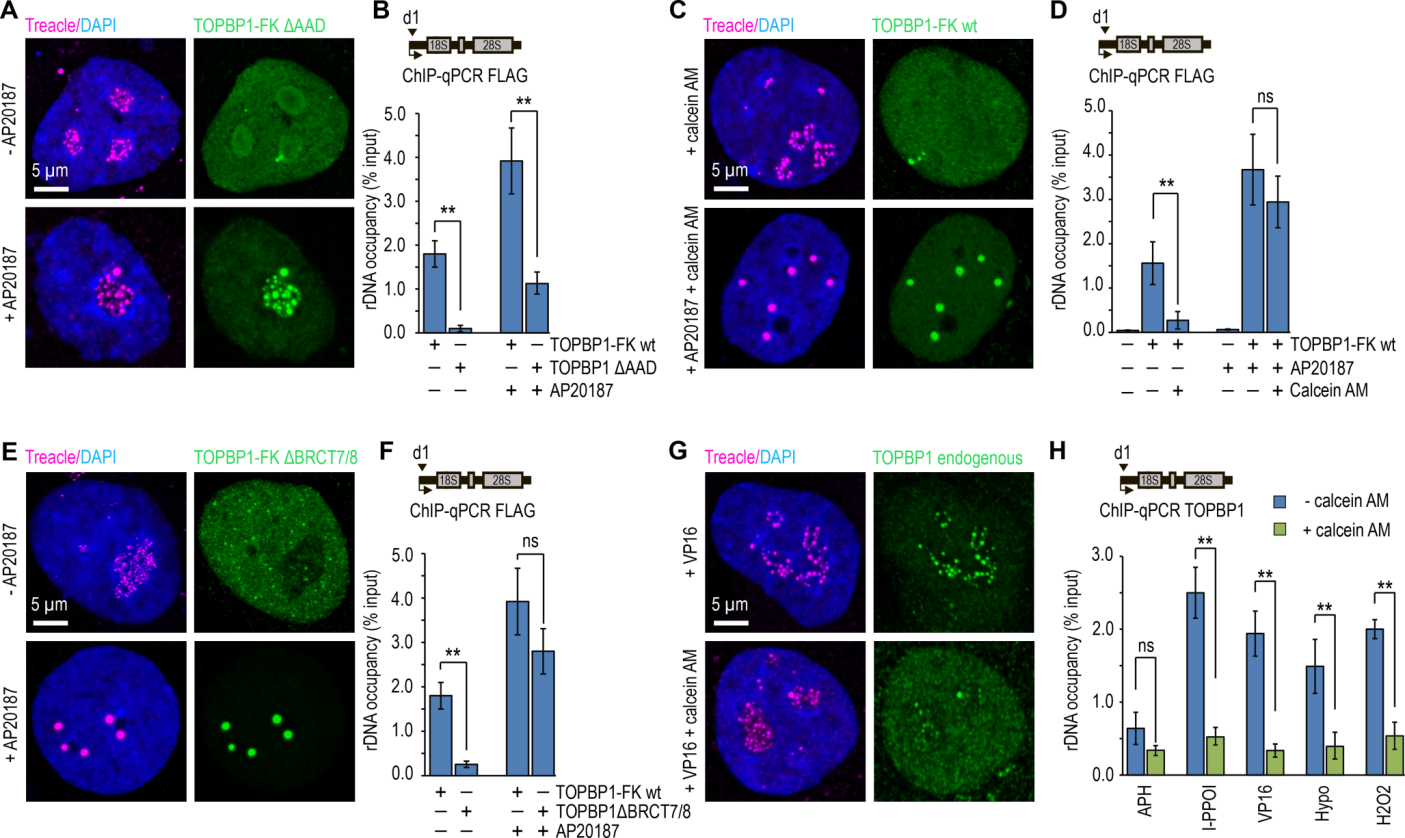
The AAD and BRCT7/8 domains of TOPBP1 are essential for Treacle-dependent condensation under endogenous nucleolar stress conditions. (**A**) HeLa TOPBP1 kn cells were transfected with TOPBP1-FK ΔAAD. For induction of TOPBP1 oligomerization, transfected cells were incubated with 100 nM AP20187 for 24 h. After 24 h cells were fixed and co-immunostained for FLAG (green) and Treacle (magenta) antibodies and analyzed by laser scanning confocal microscopy. DNA was stained with DAPI (blue). (**B**) HeLa TOPBP1 kn cells were transfected with TOPBP1-FK wt or TOPBP1-FK ΔAAD. For induction of TOPBP1 oligomerization, transfected cells were incubated with 100 nM AP20187 for 24 h. ChIP experiments were performed with antibodies against FLAG. ChIP was followed by qPCR using the d1 primers to the promoter of the rRNA gene. Data are represented relative to the input. Values are means ± SD from at least three independent replicates. **P <.01, n.s., not significant by unpaired *t*-test. (**C**) HeLa cells were transfected with TOPBP1-FLAF-FKBP12F36V (TOPBP1-FK) at a quantity of 500 ng plasmids per 2 × 10^5^ cells. For induction of TOPBP1 oligomerization, transfected cells were incubated with 100 nM AP20187 for 24 h. After 24 h cells were treated with 5 µM calcein AM for 5 h, fixed and co-immunostained for FLAG (green) and Treacle (magenta) antibodies and analyzed by laser scanning confocal microscopy. DNA was stained with DAPI (blue). (**D**) HeLa cells were processed as described in panel (C). Untransfected cells were used as controls. ChIP experiments were performed with antibodies against FLAG. ChIP was followed by qPCR using the d1 primers to the promoter of the rRNA gene. Data are represented relative to the input. Values are means ± SD from at least three independent replicates. **P <.01, n.s., not significant by unpaired *t*-test. (**E**) HeLa TOPBP1 kn cells were transfected with TOPBP1-FK ΔBRCT7/8. For induction of TOPBP1 oligomerization, transfected cells were incubated with 100 nM AP20187 for 24 h. After 24 h cells were fixed and co-immunostained for FLAG (green) and Treacle (magenta) antibodies and analyzed by laser scanning confocal microscopy. DNA was stained with DAPI (blue). (**F**) HeLa cells were processed as described in panel (E). ChIP experiments were performed with antibodies against FLAG. ChIP was followed by qPCR using the d1 primers to the promoter of the rRNA gene. Data are represented relative to the input. Values are means ± SD from at least three independent replicates. **P <.01, n.s., not significant by unpaired *t*-test. (**G**) Cells were pretreated with 5 μM calcein AM for 5 h and subsequently exposed to 90 μM etoposide (VP16) for 30 min. Next, cells were fixed, co-immunostained for FLAG (green) and Treacle (magenta) antibodies and analyzed by laser scanning confocal microscopy. DNA was stained with DAPI (blue). (**H**) To induce replicative stress, cells were treated with 1 μM APH for 16 h, followed by a 5-h treatment with 5 μM calcein AM. For the induction of rDNA DSBs, cells were transiently transfected with an I-PPOI-expressing plasmid and, 24 h post-transfection, treated with 5 μM calcein AM for 5 h, followed by 1 μM 4-OHT for 30 min. In parallel, cells were pretreated with 5 μM calcein AM for 5 h and subsequently exposed to 90 μM etoposide (VP16), 300 μM hydrogen peroxide (H₂O₂), or subjected to hypotonic stress for 30 min. In all cases, cells treated with the same DNA-damaging agents but without calcein AM served as negative controls. Cells were then fixed and subjected to ChIP using anti-TOPBP1 antibodies, followed by qPCR with primers targeting the rRNA gene promoter (d1 primers). Data are presented as % input. Values represent mean ± SD from at least three independent experiments. **P <.01; n.s., not significant (unpaired *t*-test).

Using a TOPBP1-FK construct lacking the AAD (TOPBP1-FK ΔAAD), we first examined the impact of AAD deletion on the ability of TOPBP1 to undergo Treacle-dependent condensation and associate with rDNA. In the absence of forced oligomerization, this variant failed to form TT condensates and did not associate with rDNA, unlike WT TOPBP1-FK (TOPBP1-FK wt; Fig. 5A and B). However, FKBP-induced oligomerization partially restored the ability of TOPBP1-FK ΔAAD to condense with Treacle in the FC and bind rDNA, albeit with markedly reduced efficiency (Fig. 5A and B). Notably, condensate formation in these rescue assays was fully dependent on CK2 activity but did not require ATR (Supplementary Fig. S5A and B). These results suggest that, in the absence of the AAD, condensation initiation relies primarily on CK2-mediated Treacle Ser1191 phosphorylation.

A similar phenotype was observed in cells expressing TOPBP1-FK wt upon ATRi: ATRi completely suppressed WT TOPBP1-FK-mediated TT condensate formation and rDNA binding, but enforced oligomerization partially rescued both processes (Supplementary Fig. S5C and D). Moreover, despite a significant reduction in the number of TOPBP1 condensates following ATRi treatment, the remaining residual nucleolar condensates were competent for rDNA binding. As with TOPBP1-FK ΔAAD, these residual condensates were sensitive to CK2i (Supplementary Fig. S5C and D).

Importantly, the efficiency of condensate formation by TOPBP1-FK ΔAAD, even in the context of enforced oligomerization, was markedly lower than that of WT TOPBP1-FK upon ATRi. This observation suggests that ΔAAD only partially phenocopies ATR inhibition, as even in the absence of ATR signaling, the presence of an intact AAD enhances TOPBP1 phase separation capacity. Notably, this result may reflect the intrinsically disordered nature of the AAD, which possesses an inherent ability to undergo phase separation even in the unphosphorylated state [29, 30].

In addition to the AAD, the BRCT7/8 domain also contributes to TOPBP1 condensation *in vitro* and may mediate TOPBP1 oligomerization under certain stress conditions [29, 30, 40]. Therefore, to evaluate the role of BRCT7/8 in Treacle-dependent condensation, we leveraged Calcein AM, a cell-permeable Calcein analog that binds the BRCT7/8 domain and inhibits TOPBP1 oligomerization [41]. Prior to oligomerization, Calcein AM completely suppressed TT condensate formation and rDNA binding by TOPBP1. However, it had no effect on pre-assembled FKBP-dependent condensates, likely because FKBP-mediated oligomerization bypassed the need for the BRCT7/8 domain (Fig. 5C and D). A similar result was obtained using a TOPBP1-FK construct lacking BRCT7/8 (TOPBP1-FK ΔBRCT7/8); this variant failed to form condensates or associate with rDNA at baseline but formed fully functional TT condensates comparable to those formed by TOPBP1-FK wt following oligomerization (Fig. 5E and F). These data suggest that the BRCT7/8 domain is essential for spontaneous condensation of overexpressed TOPBP1 but dispensable in the context of artificial oligomerization.

We next asked how TOPBP1 condensation is initiated under physiological conditions of genotoxic rDNA damage. Notably, it remained unclear whether nucleolar TOPBP1 condensation during stress reflects increased TOPBP1 expression or instead results from enhanced oligomerization at constant protein levels. To address this, we examined TOPBP1 abundance by immunoblotting following various genotoxic treatments. In contrast to ectopic overexpression, which markedly increased TOPBP1 levels, none of the genotoxic stresses used in this study altered endogenous TOPBP1 abundance (Supplementary Fig. S5E and F). These findings indicate that TOPBP1 condensation in FCs is not driven by increased protein expression but is instead triggered by stress-induced activation of its oligomerization capacity. Given these observations, we assessed the role of the BRCT7/8 domain in TOPBP1 nucleolar recruitment under genotoxic stress. Calcein AM treatment markedly reduced both TOPBP1 accumulation in the FC and its binding to rDNA in response to genotoxic agents (Fig. 5G and H). However, the inhibitory effect of Calcein AM was somewhat weaker than that of ATRi. This result implies that under genotoxic stress, ATR activation is the primary trigger for TOPBP1 condensation, whereas BRCT7/8-dependent oligomerization only amplifies the condensation.

Taken together, our data support the following model. Under genotoxic stress, Treacle-dependent TOPBP1 condensation in FCs is initiated by ATR activation, which both phosphorylates Treacle at Ser1199 to promote TOPBP1 binding and enhances TOPBP1 phase separation through a positive feedback loop involving the AAD. The BRCT7/8 domain further reinforces and stabilizes condensate formation via its oligomerization capacity. In contrast, under conditions of TOPBP1 overexpression, spontaneous BRCT7/8-mediated interactions can initiate condensation independently of stress, subsequently engaging ATR signaling and its associated feedback mechanisms. Finally, enforced FKBP-mediated oligomerization can partially bypass the requirement for both ATR activity and BRCT7/8 function, underscoring the intrinsic plasticity and phase separation–driven nature of TOPBP1 regulation.

### Treacle-dependent TOPBP1 condensation promotes n-DDR activation and transcriptional repression of rDNA under genotoxic stress

**Figure 6.**
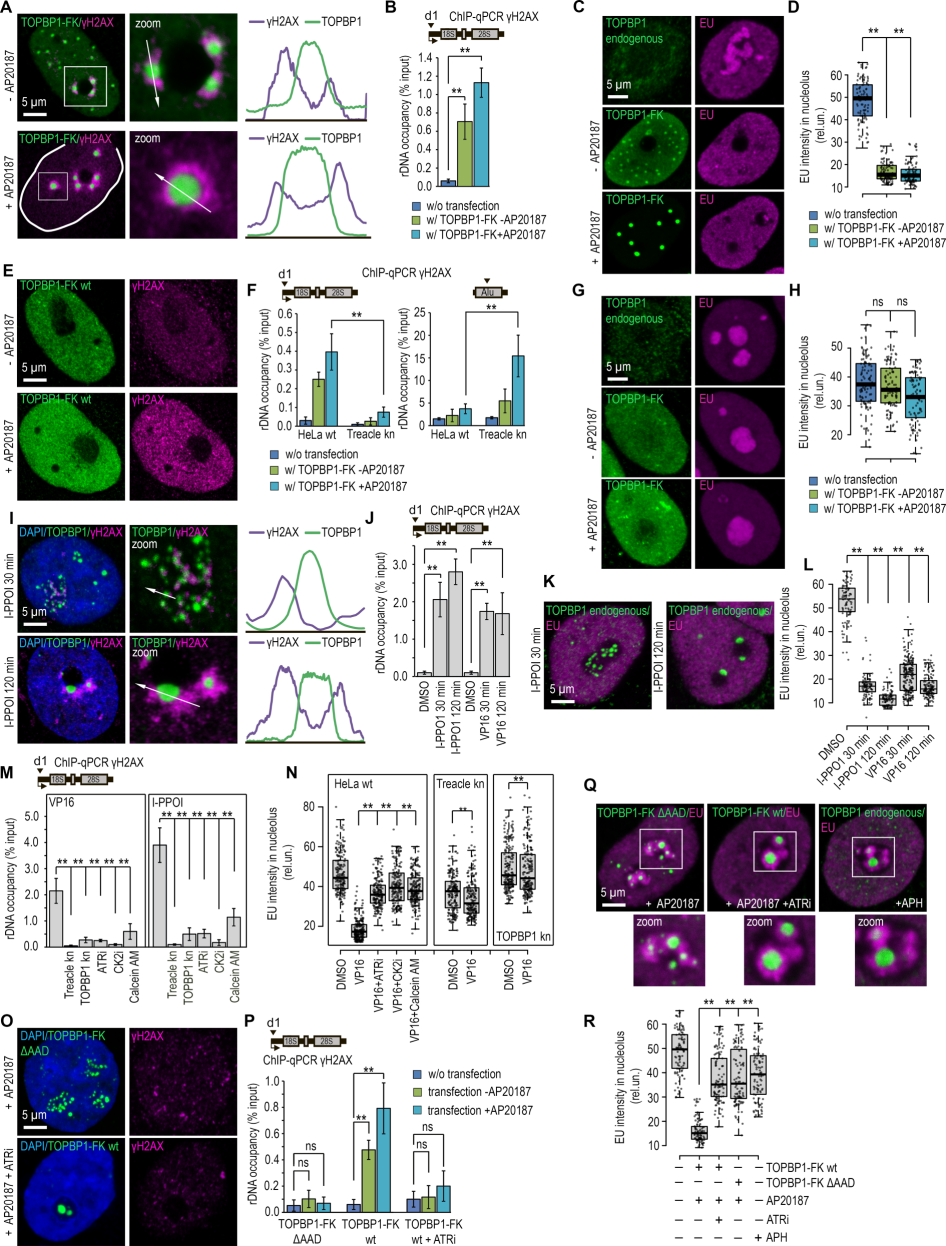
Treacle-dependent TOPBP1 condensation mediates n-DDR activation and rDNA transcriptional repression under genotoxic stress. (**A**) HeLa cells were transfected with TOPBP1-FLAF-FKBP12F36V (TOPBP1-FK). For induction of TOPBP1 oligomerization, transfected cells were incubated with 100 nM AP20187 for 24 h. The cells were fixed 24 h after transfection, co-immunostained for FLAG antibodies (green) and γH2AX (magenta) and analyzed by laser scanning confocal microscopy. Co-localization analysis was performed on the merged images. Graphs illustrate quantification in arbitrary units of fluorescence distribution along the lines shown in the figures. (**B**) HeLa cells were processed as described in panel (A). ChIP experiments were performed with antibodies against γH2AX. Untransfected cells were used as controls. ChIP was followed by qPCR using the d1 primers to the promoter of the rRNA gene. Data are represented relative to the input. Values are means ± SD from at least three independent replicates. **P <.01, n.s., not significant by unpaired *t*-test. (**C**) HeLa cells were transfected with TOPBP1-FLAF-FKBP12F36V (TOPBP1-FK). Untransfected cells were used as controls. For induction of TOPBP1 oligomerization, transfected cells were incubated with 100 nM AP20187 for 24 h. Twenty-four hours after transfection cells were treated with 100 μM EU for 1 h, fixed and immunostained for TOPBP1 antibodies (green). EU was revealed by click chemistry (magenta) and analyzed by laser scanning confocal microscopy. (**D**) HeLa cells were processed as described in panel (С). The EU intensity per nucleolus was measured (*n* > 50). **P <.01, n.s., not significant by unpaired *t*-test. (**E**) HeLa Treacle kn cells were transfected with TOPBP1-FK. For induction of TOPBP1 oligomerization, transfected cells were incubated with 100 nM AP20187 for 24 h. The cells were fixed 24 h after transfection, co-immunostained for FLAG antibodies (green) and γH2AX (magenta) and analyzed by laser scanning confocal microscopy. (**F**) HeLa cells were processed as described in panel (A). ChIP experiments were performed with antibodies against γH2AX. Untransfected cells were used as controls. ChIP was followed by qPCR using the d1 primers to the promoter of the rRNA gene or Alu repeats. Data are represented relative to the input. Values are means ± SD from at least three independent replicates. **P <.01, n.s., not significant by unpaired *t*-test. (**G**) HeLa Treacle kn cells were transfected with TOPBP1-FK. Untransfected cells were used as controls. For induction of TOPBP1 oligomerization, transfected cells were incubated with 100 nM AP20187 for 24 h. Twenty-four hours after transfection cells were pulsed with 100 μM EU for 1 h, fixed and immunostained for TOPBP1 antibodies (green). EU was revealed by click chemistry (magenta) and analyzed by laser scanning confocal microscopy. (**H**) HeLa Treacle kn cells were processed as described in panel (G). The EU intensity per nucleolus was measured (*n* > 50). **P <.01, n.s., not significant by unpaired *t*-test. (**I**) Cells were transiently transfected with a plasmid encoding I-PpoI. After 24 h, nuclear translocation of I-PpoI was triggered by treating the cells with 1 µM 4-OHT for 30 or 120 min. Cells were co-immunostained for γH2AX (magenta) and TOPBP1 (green) and analyzed by laser scanning confocal microscopy. The DNA was stained with DAPI (blue). Co-localization analysis was performed on the merged images. Graphs illustrate quantification in arbitrary units of fluorescence distribution along the lines shown in the figures. (**J**) HeLa cells were treated with 90 μM VP16 for 30 or 120 min. To induce DNA DSBs using the I-PpoI endonuclease, cells were transiently transfected with a plasmid encoding I-PpoI. After 24 h, nuclear translocation of I-PpoI was triggered by treating the cells with 1 µM 4-OHT for 30 or 120 min. DMSO-treated cells were used as controls. ChIP experiments were performed with antibodies against γH2AX. ChIP was followed by qPCR using the d1 primers to the promoter of the rRNA gene. Data are represented relative to the input. Values are means ± SD from at least three independent replicates. **P <.01, n.s., not significant by unpaired *t*-test. (**K**) HeLa cells were transiently transfected with a plasmid encoding I-PpoI. After 24 h, nuclear translocation of I-PpoI was induced by treating the cells with 1 µM 4-OHT for 30 or 120 min, in combination with 100 µM EU. Cells were fixed and co-immunostained for TOPBP1 (green). EU was revealed by click chemistry (magenta). Cells were analyzed by laser scanning confocal microscopy. (**L**) HeLa cells were processed as described in panel (J). The EU intensity per nucleolus was measured (*n* > 50). **P <.01, n.s., not significant by unpaired *t*-test. (**M**) Hela Treacle kn cells, Hela TOPBP1 kn cells or Hela wt cells pretreated with either 15 µM VE821 (ATRi) for 6 h, 30 µM CX-4945 (CK2i) for 3 h or 5 µM Calcein AM for 5 h were treated 90 μM VP16 for 30 min. To induce DNA DSBs using the I-PpoI endonuclease, Hela Treacle kn cells, Hela TOPBP1 kn cells or Hela wt cells were transiently transfected with a plasmid encoding I-PpoI. After 24 h, Hela wt cells were additionally treated with either 15 µM VE821 (ATRi) for 6 h, 30 µM CX-4945 (CK2i) for 3 h or 5 µM Calcein AM for 5 h, after which nuclear translocation of I-PpoI was triggered by treating the cells with 1 µM 4-OHT for 30 min. ChIP experiments were performed with antibodies against γH2AX. ChIP was followed by qPCR using the d1 primers to the promoter of the rRNA gene. Data are represented relative to the input. Values are means ± SD from at least three independent replicates. **P <.01, n.s., not significant by unpaired *t*-test. (**N**) Hela Treacle kn cells, Hela TOPBP1 kn cells or Hela wt cells pretreated with either 15 µM VE821 (ATRi) for 6 h, 30 µM CX-4945 (CK2i) for 3 h or 5 µM Calcein AM for 5 h were treated 90 μM VP16 for 60 min in combination with 100 μM EU. DMSO-treated cells were used as controls. EU was revealed by click chemistry. The EU intensity per nucleolus was measured (*n* > 100). **P <.01, n.s., not significant by unpaired *t*-test. (**O**) HeLa TOPBP1 kn cells were transfected with TOPBP1-FK ΔAAD or TOPBP1-FK wt. For induction of TOPBP1 oligomerization, transfected cells were incubated with 100 nM AP20187 for 24 h. After 24 h TOPBP1-FK wt transfected cells were additionally treated with 15 µM VE-821 (ATRi) for 6 h. Cells were fixed and co-immunostained for FLAG (green) and γH2AX (magenta) antibodies and analyzed by laser scanning confocal microscopy. DNA was stained with DAPI (blue). (**P**) HeLa cells were processed as described in panel (O). ChIP experiments were performed with antibodies against γH2AX. ChIP was followed by qPCR using the d1 primers to the promoter of the rRNA gene. Data are represented relative to the input. Values are means ± SD from at least three independent replicates. **P <.01, n.s., not significant by unpaired *t*-test. (**Q**) HeLa cells were transfected with TOPBP1-FK ΔAAD or TOPBP1-FK wt. For induction of TOPBP1 oligomerization, transfected cells were incubated with 100 nM AP20187 for 24 h. After 24 h TOPBP1-FK wt transfected cells were additionally treated with 15 µM VE-821 (ATRi) for 6 h. For induction replication stress Hela cells were treated with 1 μM APH for 16 h. 100 µM EU was added to the cells during the final hour of all treatments. Cells were fixed and immunostained for FLAG or TOPBP1 (green) antibodies. EU was revealed by click chemistry. (**R**) HeLa cells were processed as described in panel (Q). The EU intensity per nucleolus was measured (*n* > 50). **P <.01, n.s., not significant by unpaired *t*-test.

We and others have previously demonstrated that interactions between TOPBP1 and Treacle activate the n-DDR [7, 11, 23, 25, 42], a response that involves DNA repair kinase activation, H2AX phosphorylation (γH2AX), rRNA transcriptional repression, and recruitment of repair factors to rDNA loci. Therefore, we next sought to investigate how Treacle-dependent TOPBP1 condensation contributes to the activation of these processes. We first tested whether the formation of artificial TT condensates alone was sufficient to trigger the DDR. We observed that some TT condensates were surrounded by γH2AX foci, even prior to the induction of FKBP-mediated TOPBP1-FK oligomerization. This finding was corroborated by ChIP-qPCR analysis showing increased γH2AX levels at rDNA loci (Fig. 6A and B, and Supplementary Fig. S6A–C) and by EU incorporation assays indicating a concomitant repression of rRNA transcription (Fig. 6C and D). Upon forced oligomerization of TOPBP1-FK, γH2AX enrichment levels around all TT condensates increased (Fig. 6A and B, and Supplementary Fig. S6A–C) and further rose in rDNA, while rRNA transcription remained repressed (Fig. 6C and D). Using neutral comet assay as well as a much more specific approach—terminal deoxynucleotidyl transferase (TdT)-mediated labeling of DSBs followed by biotin-streptavidin precipitation and qPCR analysis of the precipitated DNA (TdT-IP) [25, 43, 44] we confirmed that the observed H2AX phosphorylation was not associated with the formation of single- or double-stranded DNA breaks (Supplementary Fig. S6D and E). These results indicate that TT condensates can initiate DDR signaling independently of DNA damage.

We further observed that in Treacle-kn cells, γH2AX levels still increased upon forced TOPBP1-FK oligomerization; however, the signal was delocalized and dispersed throughout the nucleus (Fig. 6E and Supplementary Fig. S6C), and nucleolar transcription was not repressed (Fig. 6G and H). This finding suggests that TOPBP1 oligomerization is sufficient to activate the DDR, whereas Treacle is essential for the spatial confinement of the response to the nucleolus. This assertion was supported by ChIP-qPCR analyses showing that γH2AX was barely detectable in rDNA but enriched at Alu repeats in Treacle-deficient cells, indicating nonspecific, genome-wide DDR activation (Fig. 6F). These findings suggest that Treacle-dependent TOPBP1 condensation can independently activate DDR signaling in the absence of DNA lesions and that Treacle is required to direct this response to rDNA loci.

We next asked whether this mechanism operates in the setting of genotoxic stress. We found that etoposide treatment and I-PPO1 endonuclease expression both repressed rRNA transcription and γH2AX accumulation in rDNA, particularly as rDNA relocates to the nucleolar periphery during nucleolar cap formation (Fig. 6I–L). Depletion of either Treacle or TOPBP1 nearly abolished genotoxic stress-induced γH2AX accumulation in rDNA and significantly attenuated repression of ribosomal gene transcription (Fig. 6M and N, and Supplementary Fig. S6F). Disruption of Treacle-dependent TOPBP1 condensation via ATRi or CK2i, as well as Calcein AM-mediated BRCT7/8 blockade, also prevented H2AX phosphorylation and rRNA transcriptional repression (Fig. 6M and N, and Supplementary Fig. S6F). Thus, Treacle-dependent TOPBP1 condensation is required for n-DDR activation and rDNA transcriptional silencing in response to genotoxic insults.

We then investigated whether Treacle-dependent condensation of TOPBP1 elicits transcriptional repression or occurs downstream of n-DDR activation. To this end, we needed to assess nucleolar transcriptional activity in a model wherein TOPBP1 condenses with Treacle without activating the n-DDR. We used the enforced TOPBP1 oligomerization system for this line of investigation because, as shown above, forced condensation of TOPBP1-FK ΔAAD (Fig. 5A and B), as well as that of TOPBP1-FK wt in the presence of ATRi (Supplementary Fig. S5C and D), preserved Treacle-dependent condensation capacity and rDNA association. Under these conditions, neither variant induced H2AX phosphorylation in the nucleolus or in rDNA (Fig. 6O and P). Moreover, EU incorporation assays revealed that nucleolar transcription remained largely unaffected in both cases, despite the presence of Treacle-dependent TOPBP1 condensates (Fig. 6Q and R). A similar pattern was observed during APH treatment, in that Treacle-dependent TOPBP1 condensation was not accompanied by global transcriptional repression (Fig. 6Q and R, and Supplementary Fig. S6G).

Together, these results suggest that transcriptional repression is triggered by n-DDR activation, not TOPBP1 condensation per se.

### Treacle-dependent TOPBP1 condensation selectively directs the recruitment of repair factors to rDNA

**Figure 7.**
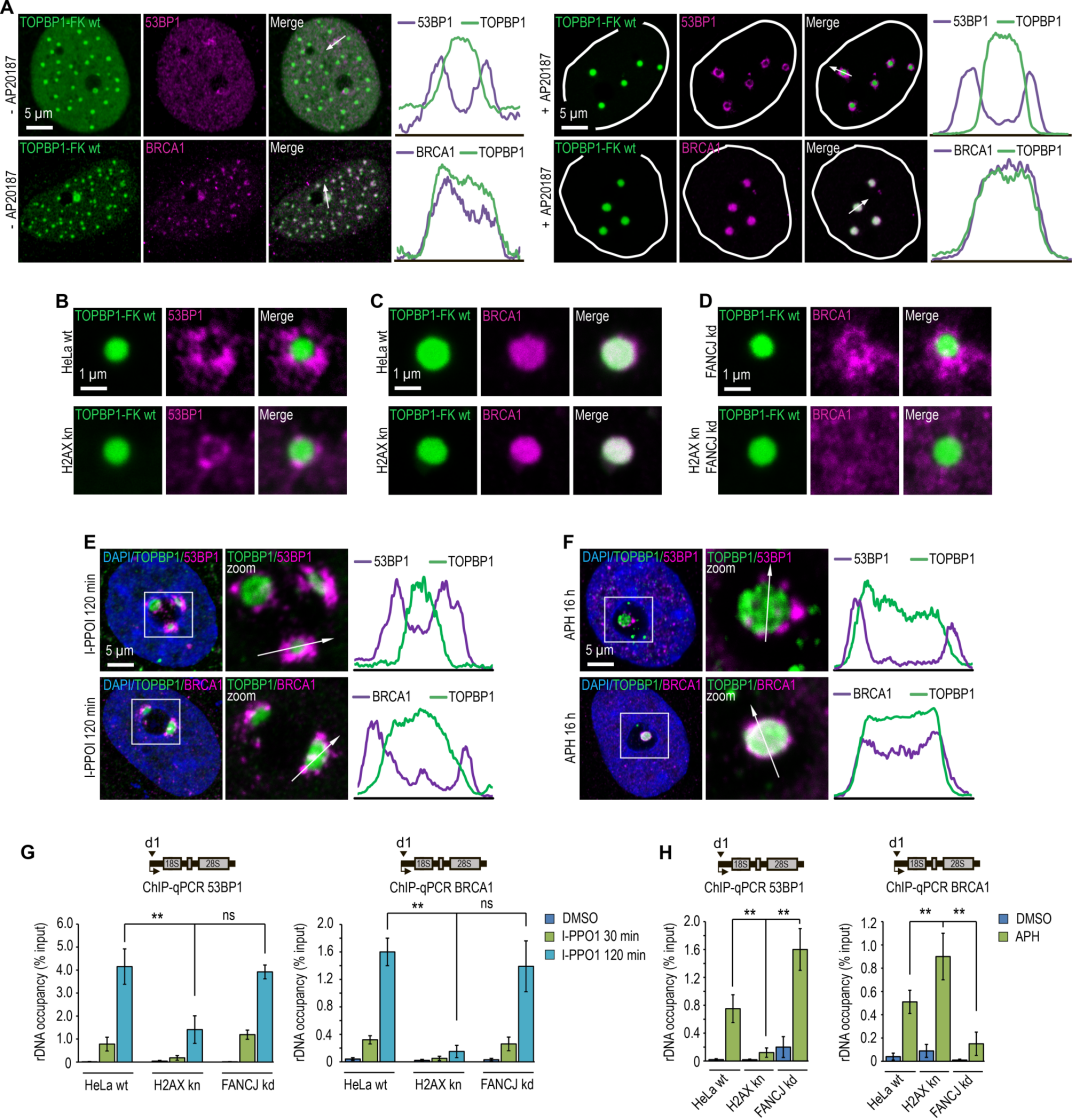
Treacle-dependent TOPBP1 condensation selectively recruits DNA repair factors 53BP1 and BRCA1 to nucleolar condensates and rDNA, depending on DNA damage context. (**A**) HeLa cells were transfected with TOPBP1-FLAF-FKBP12F36V (TOPBP1-FK). For induction of TOPBP1 oligomerization, transfected cells were incubated with 100 nM AP20187 for 24 h. The cells were fixed 24 h after transfection, co-immunostained for FLAG antibodies (green) and either 53BP1 or BRCA1 (magenta) and analyzed by laser scanning confocal microscopy. Co-localization analysis was performed on the merged images. Graphs illustrate quantification in arbitrary units of fluorescence distribution along the lines shown in the figures. (**B**) HeLa wt cells or HeLa H2AX kn cells were transfected with TOPBP1-FLAF-FKBP12F36V (TOPBP1-FK). For induction of TOPBP1 oligomerization, transfected cells were incubated with 100 nM AP20187 for 24 h. The cells were fixed 24 h after transfection, co-immunostained for FLAG antibodies (green) and 53BP1 (magenta) and analyzed by laser scanning confocal microscopy. Representative TOPBP1 condensates are show. See full image in Supplementary Fig. S7B. (**C**) HeLa wt cells or HeLa H2AX kn cells were transfected with TOPBP1-FLAF-FKBP12F36V (TOPBP1-FK). For induction of TOPBP1 oligomerization, transfected cells were incubated with 100 nM AP20187 for 24 h. The cells were fixed 24 h after transfection, co-immunostained for FLAG antibodies (green) and BRCA1 (magenta) and analyzed by laser scanning confocal microscopy. Representative TOPBP1 condensates are show. See full image in Supplementary Fig. S7C. (**D**) HeLa cells with *FANCJ* kd or HeLa H2AX kn cells with *FANCJ* kd were transfected with TOPBP1-FLAF-FKBP12F36V (TOPBP1-FK). For induction of TOPBP1 oligomerization, transfected cells were incubated with 100 nM AP20187 for 24 h. The cells were fixed 24 h after transfection, co-immunostained for FLAG antibodies (green) and BRCA1 (magenta) and analyzed by laser scanning confocal microscopy. Representative TOPBP1 condensates are show. See full image in Supplementary Fig. S7E and F. (**E**) Cells were transiently transfected with a plasmid encoding I-PpoI. After 24 h, nuclear translocation of I-PpoI was triggered by treating the cells with 1 µM 4-OHT for 120 min. Cells were fixed and co-immunostained for TOPBP1 (green) and 53BP1 or BRCA1 (magenta) and analyzed by laser scanning confocal microscopy. The DNA was stained with DAPI (blue). Co-localization analysis was performed on the merged images. Graphs illustrate quantification in arbitrary units of fluorescence distribution along the lines shown in the figures. (**F**) HeLa cells were treated with 1 μM APH for 16 h. Cells were fixed and co-immunostained for TOPBP1 (green) and 53BP1 or BRCA1 (magenta) and analyzed by laser scanning confocal microscopy. The DNA was stained with DAPI (blue). Co-localization analysis was performed on the merged images. Graphs illustrate quantification in arbitrary units of fluorescence distribution along the lines shown in the figures. (**G**) HeLa wt cells, HeLa H2AX ko cells or HeLa with *FANCJ* kd were transiently transfected with a plasmid encoding I-PpoI. After 24 h, nuclear translocation of I-PpoI was triggered by treating the cells with 1 µM 4-OHT for 30 or 120 min. ChIP experiments were performed with antibodies against 53BP1 or BRCA1. ChIP was followed by qPCR using the d1 primers to the promoter of the rRNA gene. Data are represented relative to the input. Values are means ± SD from at least three independent replicates. **P <.01, n.s., not significant by unpaired *t*-test. (**H**) HeLa wt cells, HeLa H2AX ko cells or HeLa with *FANCJ* kd were treated with 1 μM APH for 16 h. ChIP experiments were performed with antibodies against 53BP1 or BRCA1. ChIP was followed by qPCR using the d1 primers to the promoter of the rRNA gene. Data are represented relative to the input. Values are means ± SD from at least three independent replicates. **P <.01, n.s., not significant by unpaired *t*-test.

In addition to rDNA histone H2AX phosphorylation and ensuing transcriptional repression, the n-DDR is characterized by the coordinated recruitment of DNA repair factors to damage sites. Given that TOPBP1 contains multiple BRCT domains, which are well-established mediators of DNA repair protein interactions, we hypothesized that TOPBP1 spatially concentrates specific components of the DNA repair machinery within Treacle-dependent TT condensates. Indeed, previous studies have shown that the BRCT domains of TOPBP1 mediate interactions with a variety of repair proteins, including 53BP1, RAD9, BLM, FANCJ, BRCA1, and MDC1, among others [37, 39]. Moreover, the accumulation of 53BP1, BRCA1, and FANCD2 on rDNA during DSB induction or replicative stress has been attributed to interactions between TOPBP1 and Treacle [7, 23, 25]. Therefore, to assess the ability of TOPBP1 to recruit DNA repair proteins to TT condensates, we analyzed the spatial distribution of its canonical partners BRCA1 and 53BP1, as well as repair proteins not known to directly interact with TOPBP1 (KU80, RAD51, XRCC4). Prior to forced TOPBP1-FK oligomerization, weak associations of BRCA1 and 53BP1 with individual TT condensates were already evident (Fig. 7A). However, after induced TOPBP1-FK oligomerization, these proteins became strongly enriched within the condensates (Fig. 7A). Conversely, KU80, RAD51, and XRCC4 did not associate with the condensates before or after TOPBP1 oligomerization (Supplementary Fig. S7A).

Interestingly, BRCA1 localized to TT condensate cores, while 53BP1 was predominantly restricted to their peripheries. This peripheral 53BP1 localization was not dependent on its direct interaction with TOPBP1 but did require the presence of γH2AX (Fig. 7B and Supplementary Fig. S7B). In contrast, the internal localization of BRCA1 was independent of γH2AX but did require the TOPBP1 BRCT7/8 domain; BRCT7/8 domain deletion caused BRCA1 to relocate from the condensates’ cores to their peripheries (Fig. 7C and Supplementary Fig. S7C and D). Given that a direct interaction between BRCA1 and the TOPBP1 BRCT7/8 domain has not been previously described, we hypothesized that the DNA repair factor FANCJ mediates this interaction indirectly because it can bind both BRCA1 and the BRCT7/8 domain of TOPBP1 [27]. Indeed, *FANCJ* knockdown phenocopied the effect of BRCT7/8 deletion, displacing BRCA1 to the peripheries of TT condensates (Fig. 7D and Supplementary Fig. S7E). Moreover, in *FANCJ-*deficient cells, BRCA1 localization became dependent on γH2AX, similar to 53BP1 (Fig. 7D and Supplementary Fig. S7F).

We next examined whether these dependencies were preserved under different types of genotoxic stress (I-PPO1-induced DSBs and prolonged APH treatment-induced replicative stress). Following I-PPO1-induced damage, initial associations between 53BP1 and BRCA1 with rDNA were weak but increased over time as nucleolar caps formed (Fig. 7E and G). However, neither 53BP1 nor BRCA1 colocalized with TOPBP1 within the caps; instead, both localized to caps peripheries (Fig. 7E). Notably, H2AX knockout completely abolished the associations of 53BP1 and BRCA1 with rDNA, highlighting the essential role of γH2AX in mediating their recruitment to rDNA following DSBs (Fig. 7G).

Under replicative stress, 53BP1 similarly localized to the peripheries of nucleolar TOPBP1 condensates, and its association with rDNA remained γH2AX-dependent (Fig. 7F and H). In contrast, BRCA1 colocalized with TOPBP1 and maintained its association with rDNA independently of γH2AX (Fig. 7F and H). Its exclusion from rDNA was observed only upon *FANCJ* knockdown (Fig. 7H), suggesting an alternative recruitment mechanism via the TOPBP1–FANCJ complex under replicative stress.

Taken together, these findings demonstrate that Treacle-dependent TOPBP1 condensation mediates selective n-DDR spatial organization in a manner dependent on the specific type of DNA damage. Although 53BP1 recruitment is γH2AX-dependent (with γH2AX phosphorylation governed by TOPBP1 condensation) irrespective of the genotoxic stimulus in question, BRCA1 recruitment is context-dependent (γH2AX-dependent in the setting of DSBs and mediated by the FANCJ–BRCT7/8 axis under replicative stress). This divergence may reflect differences in BRCT domain accessibility or the nature of the secondary DNA structures formed during distinct stress types. Ultimately, Treacle-dependent TOPBP1 condensation functions as a versatile platform for spatiotemporal control of repair factor recruitment to rDNA, supporting either γH2AX-dependent or direct recruitment pathways based on the damage context.

### Functional role of Treacle-dependent TOPBP1 condensation in rDNA repair

**Figure 8.**
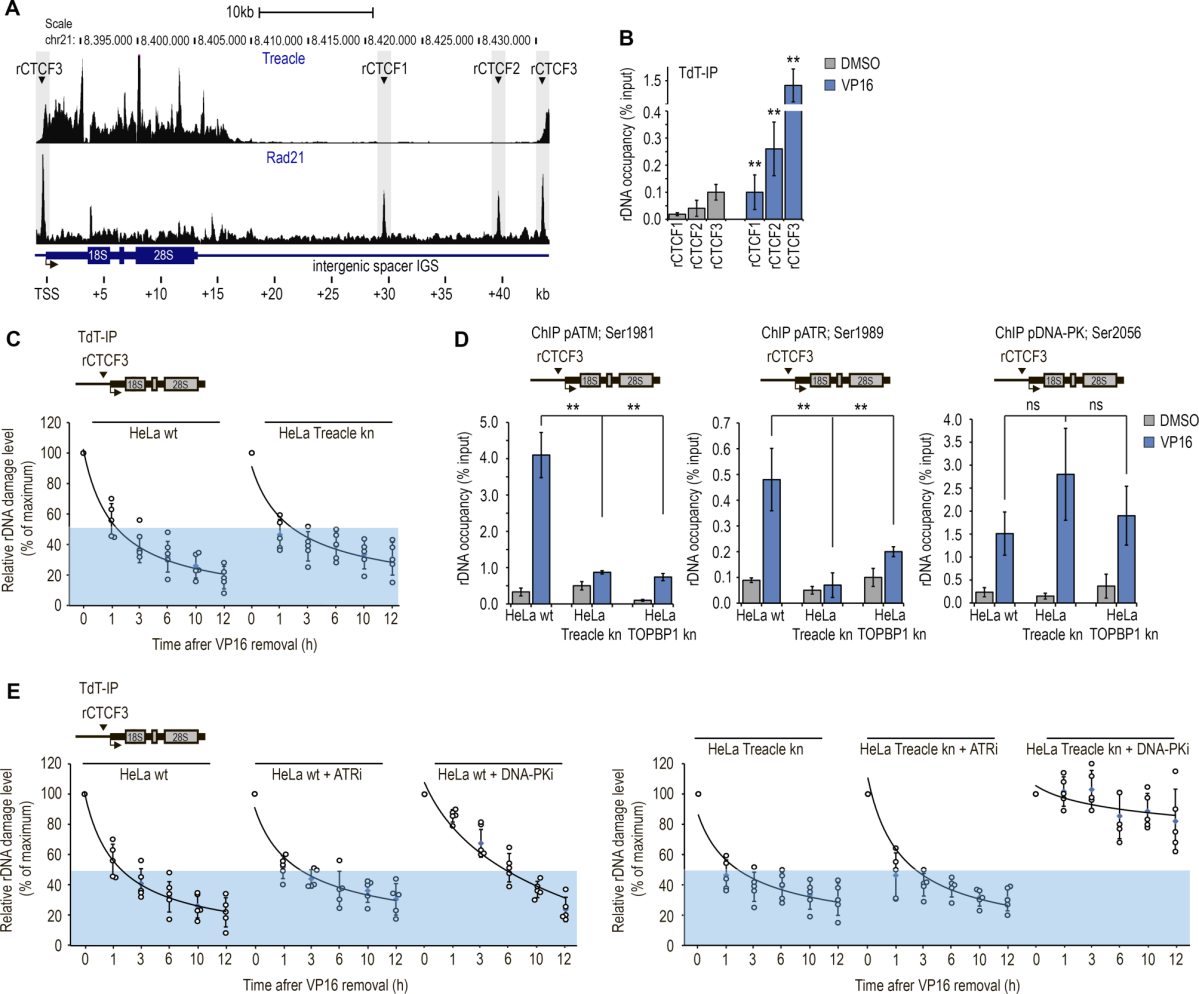
Treacle-dependent TOPBP1 signaling regulates the kinetics and pathway choice of rDNA DSB repair. (**A**) ChIP-seq distributions of Treacle and Rad21 on a ribosomal unit of chromosome 21 from control HeLa cells. (**B**) HeLa cells were treated with 90 µM etoposide for 30 min, followed by TdT-IP and quantitative PCR using rDNA amplicons corresponding to the CTCF sites positioned as indicated in the scheme shown in panel (A). DMSO-treated cells were used as a control. Data are presented relative to input. Values represent mean ± SD from at least three independent experiments. **P <.01; n.s., not significant (unpaired *t*-test). (**C**) WT HeLa cells and Treacle-knockout (Treacle-kn) HeLa cells were treated with 90 µM etoposide for 30 min, washed, and subsequently incubated in fresh culture medium for the indicated recovery times (0–12 h). At each time point, cells were fixed and subjected to TdT-IP, followed by quantitative PCR using rDNA amplicons corresponding to the promoter-associated CTCF3 site, as indicated in the scheme. DSB levels were background-subtracted and normalized to the maximal damage level detected immediately after etoposide treatment, which was set to 100%. Data represent mean ± SD from five independent biological replicates. The blue-shaded area indicates the range in which damage levels are reduced to 50% or less of the initial value. (**D**) WT HeLa cells, Treacle-knockout (Treacle-kn) HeLa cells, or TOPBP1-knockout (TOPBP1-kn) HeLa cells were treated with 90 µM etoposide for 30 min, with DMSO-treated cells used as a control. ChIP assays were performed using antibodies against phospho-Ser1981 ATM, phospho-Ser1989 ATR, or phospho-Ser2056 DNA-PK. ChIP was followed by quantitative PCR using rDNA amplicons corresponding to the promoter-associated CTCF3 site, as indicated in the scheme. Data are presented relative to input. Values represent mean ± SD from at least three independent biological replicates. **P <.01; n.s., not significant (unpaired *t*-test). (**E**) WT HeLa cells and Treacle-knockout (Treacle-kn) HeLa cells were pretreated with 15 µM VE-821 (ATRi) or 20 µM NU7441 (DNA-PKi), followed by treatment with 90 µM etoposide for 30 min. Cells were then washed to remove etoposide and incubated in fresh culture medium, either in the absence or presence of the respective inhibitors, for the indicated recovery times (0–12 h). At each time point, cells were fixed and subjected to TdT-IP, as described in panel (C).

To investigate how disruption of Treacle-dependent TOPBP1 condensation affects rDNA repair, we used etoposide as a model inducer of DSBs and analyzed the kinetics and pathway usage of rDNA repair. We first mapped the distribution of etoposide-induced DSBs across rDNA using TdT-IP. Unexpectedly, only a single robust damage site was detected within the intergenic spacer (IGS; Supplementary Fig. S8A). This nonrandom distribution suggested that etoposide-induced breaks might preferentially occur at defined regulatory sites rather than being randomly distributed across rDNA. Given that DNA topoisomerase II frequently localizes to CTCF- and cohesin-bound regions [45–47], we hypothesized that these sites might represent preferred targets of etoposide-induced damage within rDNA. To test this, we performed ChIP-seq for the cohesin subunit RAD21 and identified three discrete cohesin-binding peaks within rDNA: two within the IGS and one within the promoter region (Fig. 8A). These sites were designated CTCF1, CTCF2, and CTCF3, respectively. Notably, the strongest TdT-IP signal detected after etoposide treatment (Supplementary Fig. S8A) mapped in close proximity to the CTCF2 site. We therefore performed targeted TdT-IP using primers specific to all three CTCF-associated sites and confirmed that etoposide treatment resulted in a significant increase in DSB levels at each of these loci (Fig. 8B). Subsequent analyses focused on the promoter-proximal CTCF3 site, which exhibited the highest level of damage and overlapped with Treacle occupancy.

To assess rDNA repair dynamics, we monitored damage resolution at the CTCF3 site over a recovery time course ranging from 1 to 12 h following etoposide treatment. Surprisingly, Treacle-knockout cells did not exhibit delayed repair kinetics (Fig. 8C). Instead, during the early recovery phase (1–3 h), DSB resolution was modestly accelerated compared with WT cells. However, at later time points, repair in Treacle-knockout cells slowed, reached a plateau, and failed to achieve the level of damage clearance observed in WT cells by 12 h (Fig. 8C).

To determine which repair pathways contribute to this altered kinetic profile, we analyzed the recruitment and activation of major DDR kinases at the CTCF3 site by ChIP–qPCR. As expected, levels of activated ATM (pS1981) and ATR (pS1989) were markedly reduced in Treacle-knockout and TOPBP1-knockdown cells relative to WT cells (Fig. 8D). In contrast, the level of the auto-activated DNA-PK catalytic subunit (pS2056) was not decreased and was instead modestly elevated, indicating preserved or enhanced engagement of DNA-PK-dependent repair (Fig. 8D).

We next directly tested the contribution of ATR- and DNA-PK-dependent pathways by comparing rDNA repair kinetics in WT and Treacle-knockout cells treated with ATR or DNA-PK inhibitors. In WT cells, ATR inhibition produced a repair profile characterized by accelerated early-phase damage resolution followed by delayed late-phase repair, closely resembling the kinetics observed in Treacle-knockout cells (Fig. 8E). In contrast, DNA-PK inhibition strongly impaired but did not completely block rDNA repair. Strikingly, in Treacle-knockout cells, ATR inhibition had no detectable effect on repair dynamics, whereas DNA-PK inhibition completely abrogated rDNA repair (Fig. 8E).

Finally, we noted that inhibition of DNA-PK led to increased accumulation of TOPBP1 at rDNA under multiple genotoxic stress conditions and markedly enhanced rDNA sensitivity to genotoxic agents. Under these conditions, TOPBP1 associated with rDNA at substantially lower concentrations of genotoxic drugs, consistent with a shift in pathway usage upon suppression of DNA-PK–mediated repair (Supplementary Fig. S8B).

Together, these results indicate that Treacle-dependent TOPBP1 condensation is not required for the initial removal of rDNA DSBs but plays a critical role in amplifying ATR/ATM-dependent DDR signaling and promoting engagement of high-fidelity repair pathways. In the absence of Treacle–TOPBP1 condensation, rDNA repair proceeds predominantly via DNA-PK-dependent NHEJ, which supports rapid early damage removal but fails to sustain efficient long-term repair and complete damage resolution.

## Discussion

**Figure 9.**
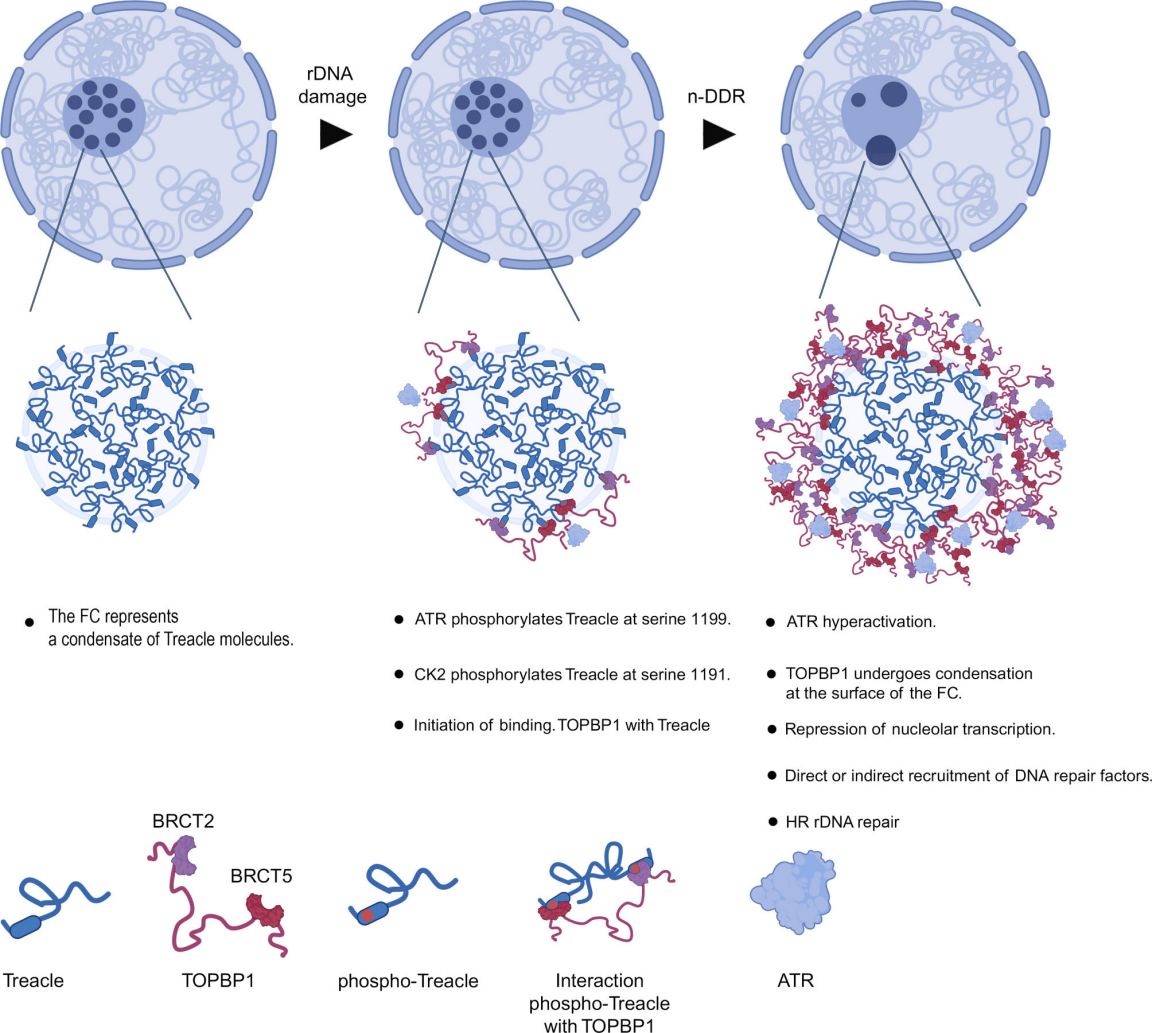
Model of Treacle-dependent TOPBP1 condensation and spatial organization of the n-DDR. The FC of the nucleolus represents a phase-separated compartment enriched in Treacle molecules (blue). Upon induction of nucleolar DNA damage, CK2 and ATR/ATM kinases cooperatively phosphorylate Treacle at serine residues 1191 and 1199, respectively. These modifications promote bivalent binding of TOPBP1 (pink) through its BRCT2 and BRCT5 domains, enabling recruitment of TOPBP1 to Treacle scaffolds. TOPBP1 subsequently undergoes phase separation at the FC through a combination of specific interactions with phosphorylated Treacle and self-association mediated by its BRCT7/8 domains. This multivalent assembly drives the formation of heterotypic Treacle–TOPBP1 condensates that amplify ATR/ATM-dependent DDR signaling, promote γH2AX formation, and facilitate repression of rRNA transcription and nucleolar cap formation. Within these caps, rDNA becomes more accessible to DNA repair factors, enabling efficient recruitment of HR-associated repair machinery. This nested, “Russian doll”-like phase architecture spatially confines nucleolar DDR signaling and coordinates the engagement of repair pathways required for accurate rDNA maintenance.

Our study reveals that Treacle acts as a nucleolar scaffold that nucleates TOPBP1 phase separation and spatially organizes DNA damage signaling at rDNA. Using reductionist systems based on inducible TOPBP1 oligomerization together with physiological models of exogenously induced rDNA damage, we compared the properties of TOPBP1 condensates formed in different contexts and defined their shared molecular principles. We show that nucleolar TOPBP1 condensation occurs at the surface of phosphorylated Treacle (Fig. 9) and requires cooperative kinase activity as well as the specific domain architecture of the interacting proteins.

Previous studies demonstrated that the Treacle–TOPBP1 interaction depends on phosphorylation of Treacle at Ser1191 and Ser1199 [23, 48]. Our findings refine the molecular architecture of this interaction: Ser1191 is phosphorylated by CK2 and recognized by the BRCT2 domain of TOPBP1, whereas Ser1199 is phosphorylated by ATR/ATM and interacts with the TOPBP1 BRCT5 domain (Fig. 9). Importantly, these interactions are not merely passive binding events but initiate a phase-separation process in which TOPBP1 assembles a partially autonomous condensate on the surface of the Treacle compartment. In this context, Treacle functions as a molecular platform that nucleates TOPBP1 condensation.

Interestingly, phosphorylation of Treacle at Ser1199 appears to depend on the type of DNA damage and the primary sensor involved. In the TOPBP1 overexpression model, in which TT condensates form without direct DNA damage, activation occurs primarily through ATR. Similarly, ATR regulates Ser1199 phosphorylation under conditions that generate extended single-stranded DNA, such as replication stress induced by APH or hypotonic stress-mediated rDNA R-loop formation. By contrast, in the presence of DSBs (e.g. induced by I-PpoI, hydrogen peroxide, or VP16), ATR and ATM appear to act cooperatively. CK2-dependent Ser1191 phosphorylation, in turn, may be constitutive or stress-enhanced, consistent with previous observations that Treacle undergoes CK2-mediated hyperphosphorylation even in the absence of genotoxic stress [3, 49].

The interaction between TOPBP1 and Treacle is also likely governed by specific stoichiometric constraints. Several observations support this notion. First, our previous work showed that partial Treacle depletion, which preserves Treacle within FCs, completely abolishes TOPBP1 recruitment during genotoxic stress [7], suggesting that an excess of Treacle relative to TOPBP1 may be required for efficient condensation. Second, disruption of Treacle phase separation impairs TOPBP1 binding, indicating that the density and structural organization of the Treacle scaffold are critical for this interaction [7]. Based on these observations and the requirement for cooperative recognition of phosphorylated Ser1191 and Ser1199 by the BRCT2 and BRCT5 domains of TOPBP1, respectively, we propose that a single TOPBP1 molecule can bridge two Treacle molecules phosphorylated at these sites (Fig. 9).

Following its initial recruitment by Treacle, TOPBP1 forms a partially autonomous phase-separated compartment. This process likely relies on weak electrostatic and hydrophobic interactions, as it is strongly sensitive to increased ionic strength (e.g. sorbitol) and moderately sensitive to 1,6-hexanediol. Intrinsically disordered regions of TOPBP1, including the AAD and linker segments between BRCT domains (Supplementary Fig. S2B and [29], [30]), likely contribute to this process through multivalent interactions typical of phase-separating proteins. This organization is consistent with the “stickers-and-spacers” model, in which BRCT domains function as phospho-binding “stickers” while flexible linkers serve as “spacers” that provide conformational mobility [50–53]. TOPBP1 phase separation may also be reinforced by intra-protein interactions. For example, the BRCT7/8 domains are capable of oligomerization [41, 54, 55], a property confirmed here to promote Treacle-dependent condensation. Additional intra- and inter-domain contacts involving BRCT0–2 and BRCT5 may further stabilize the assembly [40]. Because TOPBP1 oligomerization enhances ATR activation through a positive feedback mechanism [27, 30], these processes likely create a multilayered amplification system in which specific phospho-dependent interactions and weak multivalent contacts cooperate to stabilize and propagate TOPBP1 condensates.

Treacle-dependent TOPBP1 condensation functions as a nucleolar signaling hub that amplifies ATR/ATM-dependent DDR signaling and coordinates recruitment of repair factors to restore rDNA integrity, rather than acting as a primary sensor of rDNA DSBs. The downstream outcomes of these condensates vary with the type of stress.

Under classical DSB conditions, DDR activation first represses rRNA transcription. This repression depends on DDR signaling initiated within TOPBP1 condensates rather than on the condensates themselves: deletion of the TOPBP1 ATR-activation domain (AAD) abolishes transcriptional repression while preserving phase separation, indicating that kinase signaling is the primary effector. Potential ATM/ATR targets include RNA polymerase I subunits (RPA34, RPA1), SL1 complex components (TAF1C), and rDNA architectural regulators such as UBF and TTF1 [58, 59, 60]. Transcriptional repression is accompanied by rDNA relocation to the nucleolar periphery and formation of extranucleolar caps, enhancing accessibility to repair factors. As shown here and in parallel work by Stucki *et al*. [56], 53BP1 and BRCA1 are recruited to rDNA in a γH2AX-dependent manner. Caps likely amplify recruitment by spreading γH2AX to adjacent chromatin, which is particularly important for efficient HR initiation [16, 42].

By contrast, under replication stress, nucleolar caps and full transcriptional repression are absent, although TOPBP1 condensation still occurs. Here, BRCA1 is recruited via direct BRCT-mediated interactions in a FANCJ-dependent and γH2AX-independent manner, sufficient to restart stalled rDNA replication forks [25]. These observations highlight the functional plasticity of TOPBP1 condensates: the same phase-separated platform engages distinct downstream mechanisms depending on the nature of nucleolar stress to maintain rDNA integrity.

Notably, across all types of TOPBP1/Treacle condensates—whether induced by exogenous genotoxic stress, replication stress, hypotonic stress, or artificial oligomerization—we observed a consistent spatial segregation of ATR and ATM. As we previously showed for replication and hypotonic stress [10, 25], ATR localizes within the condensates, whereas ATM resides at the periphery together with γH2AX and 53BP1. This organization reflects ATM’s role in γH2AX phosphorylation and γH2AX-dependent recruitment of 53BP1, providing an additional layer of spatial compartmentalization within nucleolar signaling condensates.

Importantly, loss of Treacle or TOPBP1 does not abolish rDNA DSB repair per se but alters repair kinetics and pathway choice. In Treacle-deficient cells, early repair is accelerated yet relies predominantly on DNA-PK-dependent nonhomologous end joining (NHEJ), while ATR/ATM signaling and late-phase repair are impaired. Consistent with prior work showing NHEJ as the predominant rapid repair pathway for rDNA [20], these results suggest that Treacle-dependent TOPBP1 condensation facilitates timely engagement of additional repair mechanisms, including HR.

Treacle-dependent condensation is preferentially engaged under high levels of rDNA damage. Under moderate genotoxic stress, repair proceeds efficiently without detectable TOPBP1 condensates—likely via NHEJ—indicating that phase separation is not required for basal repair. Instead, condensation functions as a threshold-dependent response, triggered when damage is extensive or persistent. In this context, Treacle-dependent TOPBP1 condensation promotes robust ATR/ATM signaling, rRNA transcriptional repression, nucleolar cap formation, and recruitment of HR-associated repair factors, thereby ensuring high-fidelity repair and maintenance of rDNA integrity.

From an evolutionary perspective, the absence of Treacle in anamniotes [57] further supports the idea that NHEJ represents a conserved default repair mode for rDNA, whereas Treacle-dependent TOPBP1 condensation may have evolved to enable regulated engagement of high-fidelity HR, improving repair accuracy under conditions of elevated damage.

Together, these observations support a model in which Treacle-dependent TOPBP1 condensation does more than amplify the n-DDR: it modulates repair pathway choice, promoting ATR/ATM-dependent signaling and facilitating recruitment of HR-associated factors when rDNA damage surpasses a critical threshold. Phase separation thus emerges as a key regulatory mechanism linking damage magnitude to repair pathway engagement, ensuring long-term stability of highly repetitive and transcriptionally active rDNA arrays.

In summary, our study delineates the molecular architecture of the Treacle–TOPBP1 interaction and establishes Treacle-dependent TOPBP1 condensation as a phase-organized nucleolar DDR signaling hub that integrates damage sensing, repair pathway coordination, and repair execution to preserve rDNA integrity.

## Supplementary Material

gkag350_Supplemental_Files

## Data Availability

The sequencing raw read files generated in this study have been deposited in the Gene Expression Omnibus (GEO) repository under accession number GSE316591. Proteomics data have been deposited in the PRIDE database under project accession PXD067042 (DOI: 10.6019/PXD067042).
