# Supplementary material for: Treacle-dependent TOPBP1 condensation regulates the nucleolar DNA damage response
Source: Nucleic Acids Res. 2026 May 19;54(9):gkag350. doi: 10.1093/nar/gkag350 (PMC13183679; doi:10.1093/nar/gkag350)
Supplement: gkag350_Supplemental_Files [file gkag350_supplemental_files.zip › 140426214923_supplementary_materials.pdf]

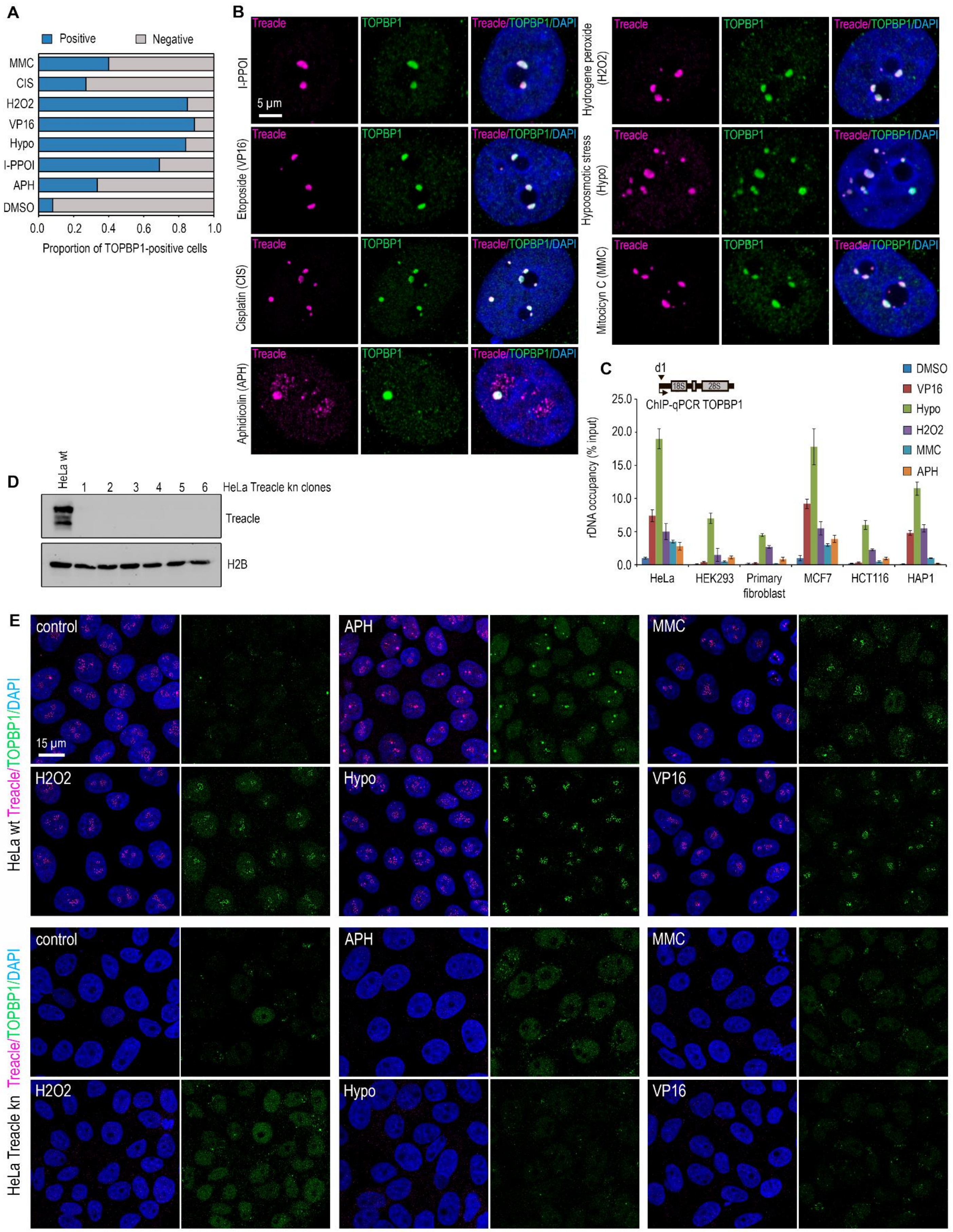

A
Positive
Negative
MMC
CIS
H2O2
VP16
Hypo
I-PPOI
APH
DMSO
0.0 0.2 0.4 0.6 0.8 1.0
Proportion of TOPBP1-positive cells
B
Treacle
TOPBP1
Treacle/TOPBP1/DAPI
I-PPOI
5 µm
Etoposide (VP16)
Cisplatin (CIS)
Aphidicolin (APH)
Hydrogene peroxide (H2O2)
Hypoosmotic stress (Hypo)
Mitocicyn C (MMC)
C
d1
18S
28S
ChIP-qPCR TOPBP1
rDNA occupancy (% input)
25.0
20.0
15.0
10.0
5.0
0.0
DMSO
VP16
Hypo
H2O2
MMC
APH
HeLa
HEK293
Primary fibroblast
MCF7
HCT116
HAP1
D
HeLa wt
1 2 3 4 5 6
HeLa Treacle kn clones
Treacle
H2B
E
control
APH
MMC
H2O2
Hypo
VP16
15 µm
HeLa wt Treacle/TOPBP1/DAPI
HeLa Treacle kn Treacle/TOPBP1/DAPI

Figure S1: Treacle is required for TOPBP1 recruitment to ribosomal DNA in response to genotoxic and nucleolar stress. (A) HeLa cells were treated with either 90 μM VP16 for 30 min, 1 μM APH for 6 h, 300 μM hydrogen peroxide (H2O2) for 30 min, 50 μM mitomycin c (MMC) for 30 min, 50 μM cisplatin (CIS) for 30 min. Hypoosmotic stress (hypo) was applied by incubation the cells in 50% DMEM/50% H2O (Hypoosmotic medium) for 30 min. To induce DNA double-strand breaks using the I-PpoI endonuclease, cells were transiently transfected with a plasmid encoding I-PpoI. After 24 hours, nuclear translocation of I-PpoI was triggered by treating the cells with 1 μM 4-hydroxytamoxifen (4-OHT) for 30 minutes. Cells were co-immunostained for Treacle (magenta) and TOPBP1 (green) and analyzed by laser scanning confocal microscopy. Percentage of cells containing TOPBP1 (TOPBP1-positive) foci within nucleoli is shown. **(B)** HeLa cells were treated with either 90 μM VP16 for 3 h, 300 μM hydrogen peroxide (H2O2) for 3 h, 50 μM mitomycin c (MMC) for 3 h, 50 μM cisplatin (CIS) for 3 h and 1 μM APH for 16 h. Hypoosmotic stress (hypo) was applied by incubation the cells in 50% DMEM/50% H2O (Hypoosmotic medium) for 3 h. To induce DNA double-strand breaks using the I-PpoI endonuclease, cells were transiently transfected with a plasmid encoding I-PpoI. After 24 hours, nuclear translocation of I-PpoI was triggered by treating the cells with 1 μM 4-hydroxytamoxifen (4-OHT) for 3 h. Next cells were fixed and co-immunostained for Treacle (magenta) and TOPBP1 (green) and analyzed by laser scanning confocal microscopy. The DNA was stained with DAPI (blue). **(C)** Hela, HEK293, Primary human skin fibroblasts, MCF7, HCT116 or HAP1 were treated with either 90 μM VP16 for 30 min, 1 μM APH for 6 h, 300 μM hydrogen peroxide (H2O2) for 30 min, 50 μM mitomycin c (MMC) for 30 min. Hypoosmotic stress (hypo) was applied by incubation the cells in 50% DMEM/50% H2O (Hypoosmotic medium) for 30 min. ChIP experiments were performed with antibodies against TOPBP1. ChIP was followed by qPCR using the d1 primers to the promoter of the rRNA gene (positioned as indicated on the scheme). Data are represented relative to the input. Values are means ±SD from at least three independent replicates. **, $p < 0.01$, n.s., not significant by unpaired t test. **(D)** HeLa cells were depleted for *TCOF1* gene (Treacle kn) using CRISPR/Cas9 technology. Knockout (ko) efficiencies were analyzed by WB. Histone H2B is used as a loading control in WB. **(E)** Intact HeLa (HeLa wt) cells or HeLa cells with CRISPR-Cas9-mediated deletion of the *TCOF1* gene (Treacle kn) were treated with either 90 μM VP16 for 30 min, 1 μM APH for 16 h, 300 μM hydrogen peroxide (H2O2) for 30 min or 50 μM mitomycin c (MMC) for 30 min Hypoosmotic stress (hypo) was applied by incubation the cells in 50% DMEM/50% H2O (Hypoosmotic medium) for 30 min. Cells were co-immunostained for Treacle (magenta) and TOPBP1 (green) and analyzed by laser scanning confocal microscopy. The DNA was stained with DAPI (blue).

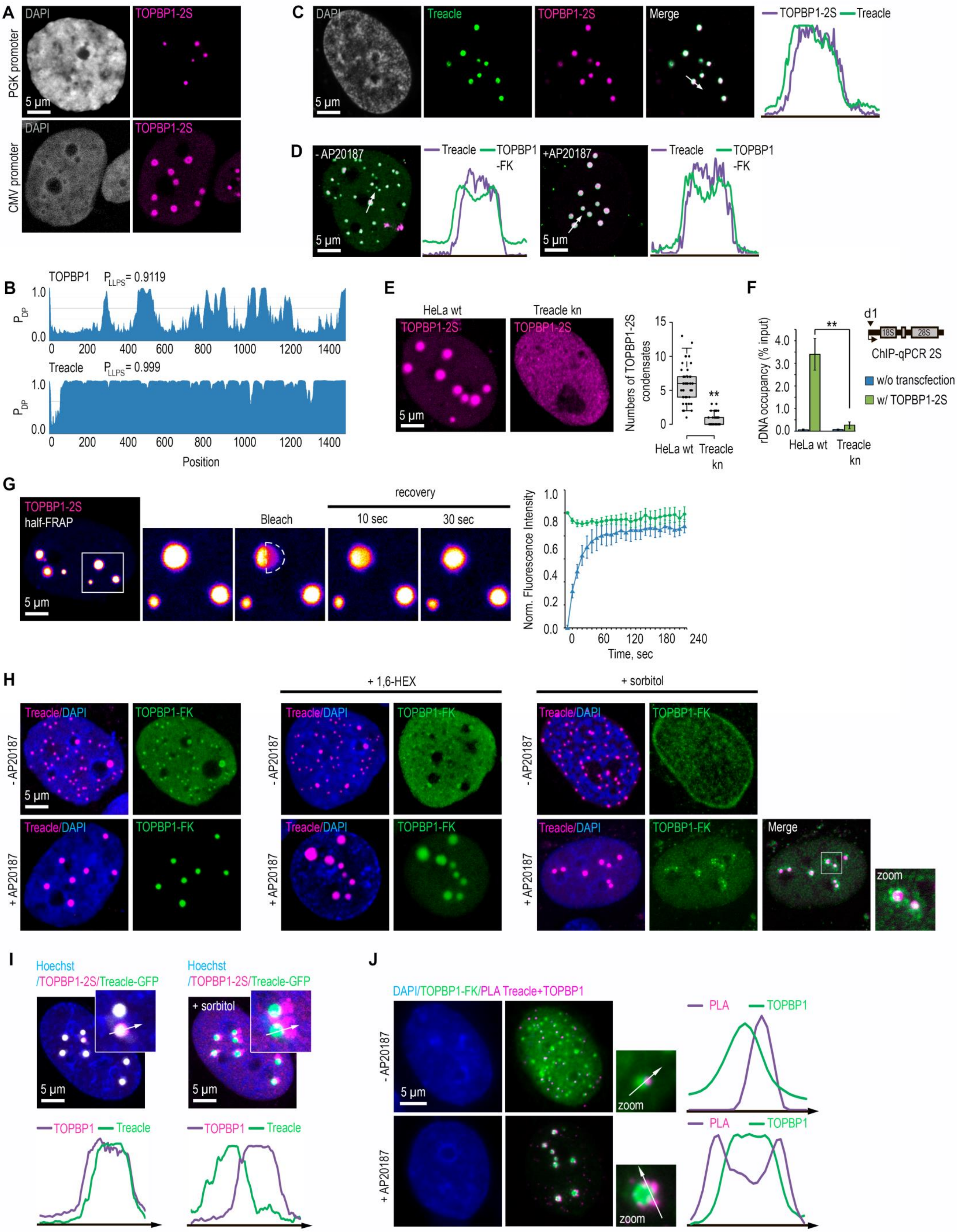

Figure S2: TOPBP1 undergoes phase separation at the surface of Treacle condensates. (A)

HeLa cells were transfected with TOPBP1-Katushka2S (TOPBP1-2S; magenta) expressed either under the control of the weak PGK promoter or the strong CMV promoter, at a dose of 500 ng plasmid per 2×10^5 cells at a quantity of 500 ng plasmids per $2x10^5$ cells. Cells were fixed 24 hours after transfection. DNA was stained with DAPI (gray). Cells were analyzed by laser scanning confocal microscopy. **(B)** Full-length Treacle and TOPBP1 disorder characteristics predicted by FuzDrop, **(C)** HeLa cells were transfected with TOPBP1-Katushka2S (TOPBP1-2S; magenta) at a quantity of 500 ng plasmids per $2x10^5$ cells. Cells were fixed 24 hours after transfection and immunostaining with antibodies against Treacle (green). DNA was stained with DAPI (gray). Co-localization analysis was performed on the merged images. Graphs illustrate quantification in arbitrary units of fluorescence distribution along the lines shown in the figures. **(D)** HeLa cells were transfected with TOPBP1-FLAG–FKBP12F36V (TOPBP1-FK), expressed under the control of the strong CMV promoter. To induce TOPBP1 oligomerization, cells were treated with 100 nM AP20187 for 24 h. The cells were fixed 24 hours after transfection, co-immunostained for FLAG (green) and Treacle (magenta) antibodies and analyzed by laser scanning confocal microscopy. Co-localization analysis was performed on the merged images. Graphs illustrate quantification in arbitrary units of fluorescence distribution along the lines shown in the figures. **(E)** HeLa wt and HeLa cells with CRISPR-Cas9-mediated deletion of the *TCOF1* gene (Treacle kn) were transiently transfected with plasmid constructs encoding TOPBP1-2S (magenta). The number of TOPBP1-2S condensates per cell was measured (n>50). **, $p < 0.01$, n.s., not significant by unpaired t test. **(F)** HeLa cells were processed as described in (E). Untransfected cells were used as controls. ChIP experiments were performed with antibodies against 2S. ChIP was followed by qPCR using the d1 primers to the promoter of the rRNA gene. Data are represented relative to the input. Values are means ±SD from at least three independent replicates. **, $p < 0.01$, n.s., not significant by unpaired t test. **(G)** HeLa cells were transiently transfected with TOPBP1-2S. 24 h after transfection half-FRAP analysis of TOPBP1-2S condensates was performed. Half of the one condensate was bleached, and fluorescence recovery for TOPBP1-2S was monitored. Representative time-lapse images of the photobleached condensates are shown (magnified images). DNA was stained with Hoechst 33342 (gray). Graphs illustrate the quantification of TOPBP1-2S the half-FRAP analysis. Half-FRAP curves represent the normalized intensity in the bleached half (blue) and the non-bleached half (green). Each trace represents an average of measurements for at least twenty condensates; error bars represent SD. **(H)** HeLa cells were transfected with TOPBP1-FLAF-FKBP12F36V (TOPBP1-FK; green) at a quantity of 500 ng plasmids per $2x10^5$ cells. For induction of TOPBP1 oligomerization, transfected cells were incubated with 100 nM AP20187 for 24 h. 24 hours after transfection cells were treated with 5% 1,6-hexanediol (1,6-HEX) for 10 min or 300 mM sorbitol for 30 min. The cells were fixed and immunostained for Treacle (magenta) antibodies. DNA was stained with DAPI (blue). Cells were analyzed by laser scanning confocal microscopy. **(I)** HeLa cells were transiently co-transfected with Treacle-GFP and TOPBP1-2S. 24 h after transfection with TOPBP1-2S, HeLa cells were live time-lapse imaged. DNA was stained with Hoechst 33342 (blue). Cells were imaged using a z-stack scan mode, followed by treatment with 300 μM sorbitol for 30 minutes and subsequent re-imaging. Co-localization analysis was performed on the merged images. Graphs illustrate quantification in arbitrary units of fluorescence distribution along the lines shown in the figures. **(J)** HeLa cells were transfected with TOPBP1-FLAF-FKBP12F36V (TOPBP1-FK; green) at a quantity of 500 ng plasmids per $2x10^5$ cells. For induction of TOPBP1 oligomerization, transfected cells were incubated with 100 nM AP20187 for 24 h. 24 hours after transfection cells were subjected to proximity ligation assay (PLA) with antibodies against TOPBP1 and Treacle

(magenta). Additionally, TOPBP1 condensates were visualized by immunostaining with secondary antibodies conjugated to Alexa Fluor 488 (green). DNA was stained with DAPI (blue). Co-localization analysis was performed on the merged images. Graphs illustrate quantification in arbitrary units of fluorescence distribution along the lines shown in the figures.

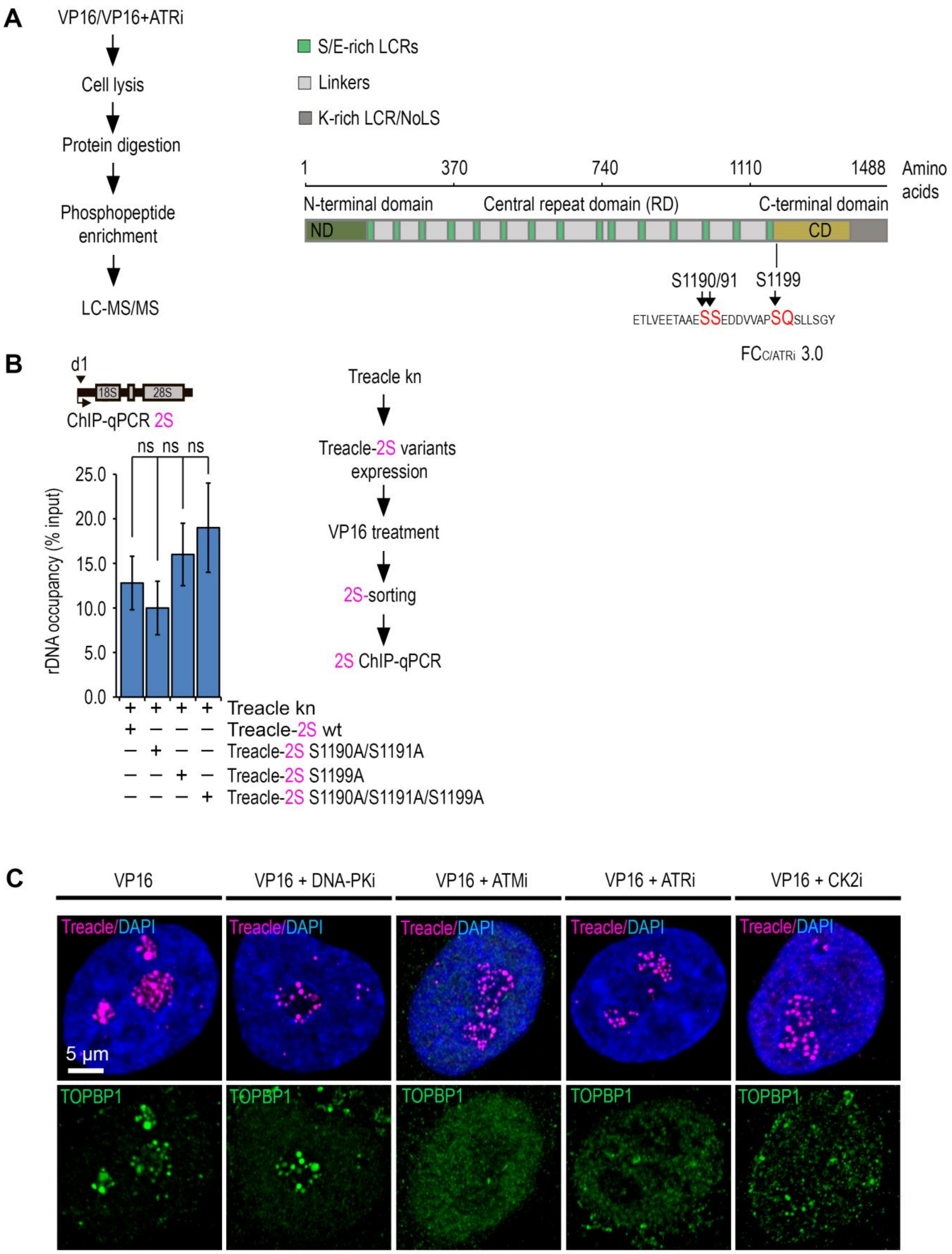

Figure S3: Condensation of TOPBP1 requires ATR/CK2-dependent phosphorylation of Treacle. (A) HeLa cells were treated with either 90 µM VP16 for 30 min, or preincubated with 15 µM VE-821 (ATRi) for 3 h and then treated with 90 µM VP16 for 30 min. Next cells were lysed and subjected to phospho-proteomic analysis. The structure of the most common isoform of Treacle (Treacle isoform d, 1488 amino acids, NP_001128715.1). The scheme shows the positions

of phospho-serines in the C-terminal domain of the Treacle found in the phospho-proteomic analysis. FC denotes the fold change in serine 1199 phosphorylation levels in response to ATRi. **(B)** HeLa cells with CRISPR-Cas9-mediated deletion of the TCOF1 gene (Treacle kn) were transiently transfected with plasmid constructs encoding either Treacle-2S wt, Treacle-2S S1190A/S1191A, Treacle-2S S1199A or Treacle-2S S1190A/S1191A/S1199A. 24 h after transfection, cells were treated with VP16 (90 μM for 30 min), fixed and subjected to cell sorting in the fluorescent analysis mode to obtain 2S-positive populations. At least $2x10^6$ sorted cells were used for ChIP with 2S antibodies. ChIP was followed by qPCR using the d1 primers to the promoter of the rRNA gene (positioned as indicated on the scheme). Data are represented relative to the input. Values are means ±SD from at least three independent replicates. **, $p < 0.01$, n.s., not significant by unpaired t test. **(C)** HeLa cells were preincubated with either 15 μM VE-821 (ATRi) for 3 h, 30 μM CX-4945 (CK2i) for 3 h, 20 μM NU7441 (DNAPKi) for 3 h or 20 μM KU55933 (ATMi) for 3 h and then treated with VP16 (90 μM) for 30 min. Next cells were fixed and co-immunostained for Treacle (magenta) and TOPBP1 (green) and analyzed by laser scanning confocal microscopy. The DNA was stained with DAPI (blue).

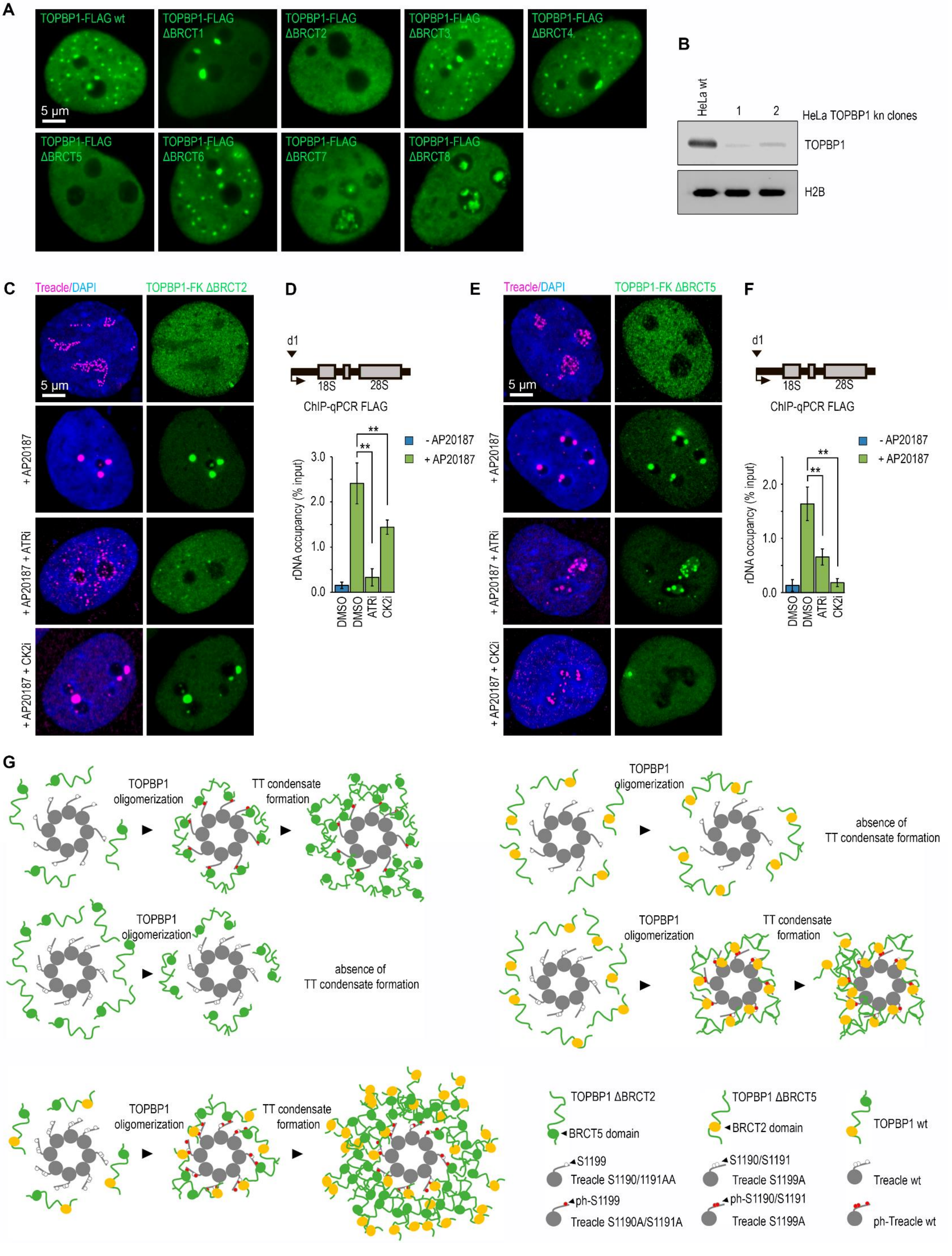

A
TOPBP1-FLAG wt
TOPBP1-FLAG ΔBRCT1
TOPBP1-FLAG ΔBRCT2
TOPBP1-FLAG ΔBRCT3
TOPBP1-FLAG ΔBRCT4
TOPBP1-FLAG ΔBRCT5
TOPBP1-FLAG ΔBRCT6
TOPBP1-FLAG ΔBRCT7
TOPBP1-FLAG ΔBRCT8
5 μm
B
HeLa wt
1
2
HeLa TOPBP1 kn clones
TOPBP1
H2B
C
Treacle/DAPI
TOPBP1-FK ΔBRCT2
+ AP20187
+ AP20187 + ATRi
+ AP20187 + CK2i
D
d1
18S
28S
ChIP-qPCR FLAG
- AP20187
+ AP20187
rDNA occupancy (% input)
DMSO
ATRi
CK2i
E
TOPBP1-FK ΔBRCT5
F
G
TOPBP1 oligomerization
TT condensate formation
absence of TT condensate formation
TOPBP1 ΔBRCT2
BRCT5 domain
TOPBP1 ΔBRCT5
BRCT2 domain
TOPBP1 wt
S1199
Treacle S1190/1191AA
S1190/S1191
Treacle S1199A
Treacle wt
ph-S1199
Treacle S1190A/S1191A
ph-S1190/S1191
ph-Treacle wt

Figure S4: BRCT2 and BRCT5 domains of TOPBP1 engage Treacle phospho-Ser1190/1191 and Ser1199 to drive TOPBP1 condensation. **(A)** HeLa TOPBP1 kn cells were transfected with either TOPBP1-FLAG ΔBRCT1, TOPBP1-FLAG ΔBRCT2, TOPBP1-FLAG ΔBRCT3, TOPBP1-FLAG ΔBRCT4, TOPBP1-FLAG ΔBRCT5, TOPBP1-FLAG ΔBRCT6, TOPBP1-FLAG ΔBRCT7, TOPBP1-FLAG ΔBRCT8. After 24 h cells were fixed and immunostained for FLAG (green) antibodies and analyzed by laser scanning confocal microscopy. **(B)** HeLa cells were depleted for *TOPBP1* gene (TOPBP1 kn) using CRISPR/Cas9 technology. Knockout (ko) efficiencies were analyzed by WB. Histone H2B is used as a loading control in WB. **(C)** HeLa TOPBP1 kn cells were transfected with TOPBP1-FK ΔBRCT0-2. For induction of TOPBP1 oligomerization, transfected cells were incubated with 100 nM AP20187 for 24 h. After 24 h cells were treated with either 15 μM VE-821 (ATRi) for 6 h or 30 μM CX-4945 (CK2i) for 3 h, fixed and co-immunostained for FLAG (green) and Treacle (magenta) antibodies and analyzed by laser scanning confocal microscopy. DNA was stained with DAPI (blue). **(D)** HeLa cells were processed as described in (C). ChIP experiments were performed with antibodies against FLAG. ChIP was followed by qPCR using the d1 primers to the promoter of the rRNA gene. Data are represented relative to the input. Values are means ±SD from at least three independent replicates. **, $p < 0.01$, n.s., not significant by unpaired t test. **(E)** HeLa TOPBP1 kn cells were transfected with TOPBP1-FK ΔBRCT5. For induction of TOPBP1 oligomerization, transfected cells were incubated with 100 nM AP20187 for 24 h. After 24 h cells were treated with either 15 μM VE-821 (ATRi) for 6 h or 30 μM CX-4945 (CK2i) for 3 h, fixed and co-immunostained for FLAG (green) and Treacle (magenta) antibodies and analyzed by laser scanning confocal microscopy. DNA was stained with DAPI (blue). **(F)** HeLa cells were processed as described in (E). ChIP experiments were performed with antibodies against FLAG. ChIP was followed by qPCR using the d1 primers to the promoter of the rRNA gene. Data are represented relative to the input. Values are means ±SD from at least three independent replicates. **, $p < 0.01$, n.s., not significant by unpaired t test. **(G)** Cartoons illustrate the proposed mechanism whereby oligomerized TOPBP1-FK is recruited to Treacle through phospho-dependent BRCT interactions. BRCT2 recognizes Treacle pSer1190/1191, whereas BRCT5 binds pSer1199. TT condensates form only when at least one cognate phospho-site/BRCT interaction is preserved (Treacle-2S S1190A/S1191A with TOPBP1-FK ΔBRCT2, or Treacle-2S S1199A with TOPBP1-FK ΔBRCT5). Disruption of both interfaces prevents TOPBP1 recruitment and abolishes TT condensate formation.

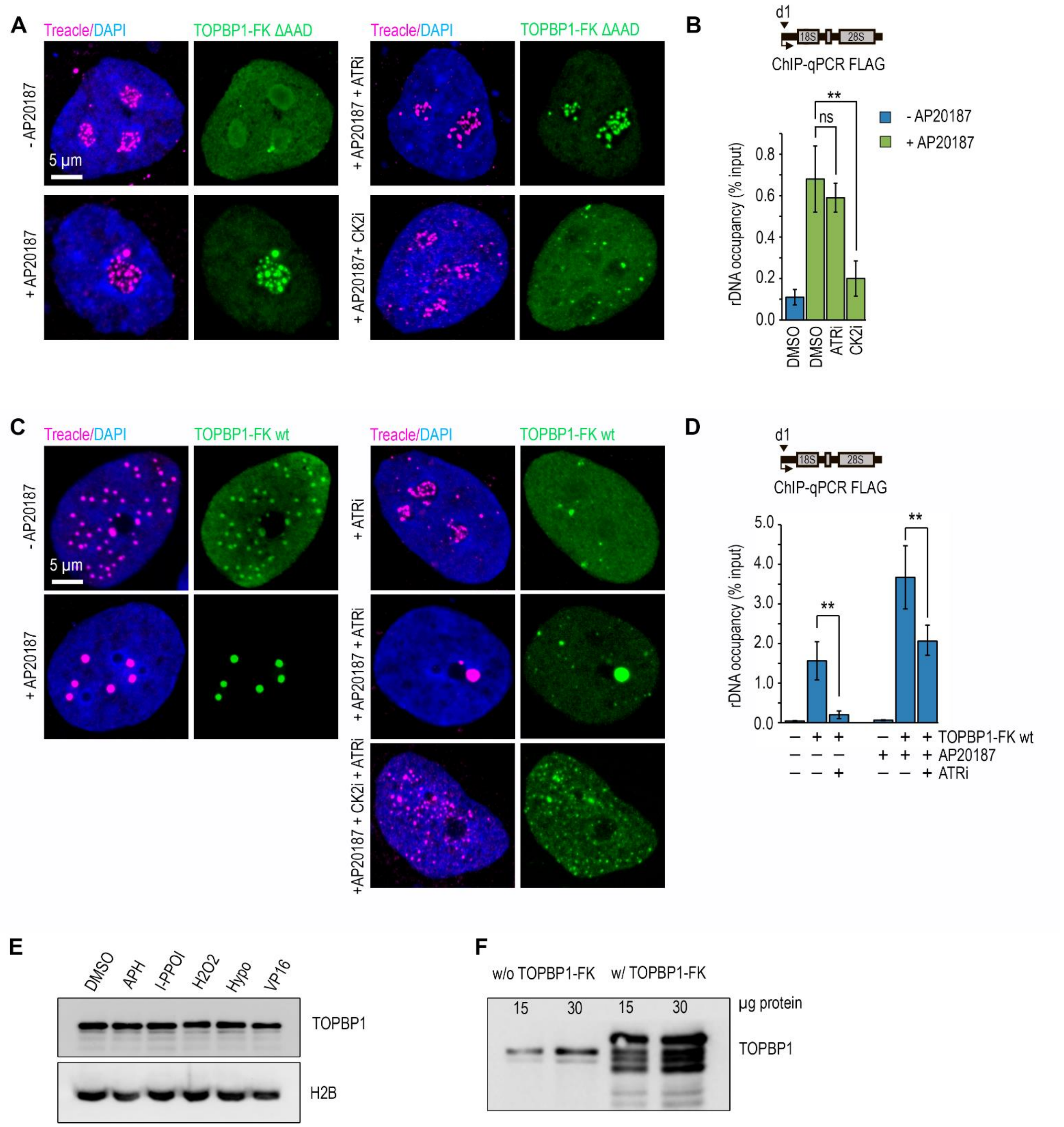

Figure S5: The AAD and BRCT7/8 domains of TOPBP1 are essential for Treacle-dependent condensation under endogenous nucleolar stress conditions. (A) HeLa TOPBP1 kn cells were transfected with TOPBP1-FK ΔAAD. For induction of TOPBP1 oligomerization, transfected cells were incubated with 100 nM AP20187 for 24 h. After 24 h cells were treated with either 15 µM VE-821 (ATRi) for 6 h or 30 µM CX-4945 (CK2i) for 3 h, fixed and co-immunostained for FLAG (green) and Treacle (magenta) antibodies and analyzed by laser scanning confocal microscopy. DNA was stained with DAPI (blue). **(B)** HeLa cells were processed as described in (A). ChIP experiments were performed with antibodies against FLAG. ChIP was followed by qPCR using the d1 primers to the promoter of the rRNA gene. Data are represented relative to the input. Values are means ±SD from at least three independent replicates. **, $p < 0.01$, n.s., not significant by unpaired t test. **(C)** HeLa TOPBP1 kn cells were transfected with TOPBP1-FK wt. For induction

of TOPBP1 oligomerization, transfected cells were incubated with 100 nM AP20187 for 24 h. After 24 h cells were treated with either 15 μM VE-821 (ATRi) for 6 h or combination 30 μM CX-4945 (CK2i) and 15 μM VE-821 (ATRi) for 6 h. Cells were fixed and co-immunostained for FLAG (green) and Treacle (magenta) antibodies and analyzed by laser scanning confocal microscopy. DNA was stained with DAPI (blue). **(D)** HeLa cells were processed as described in (C). ChIP experiments were performed with antibodies against FLAG. ChIP was followed by qPCR using the d1 primers to the promoter of the rRNA gene. Data are represented relative to the input. Values are means ±SD from at least three independent replicates. **, $p < 0.01$, n.s., not significant by unpaired t test. **(E)** HeLa cells were treated with either 90 μM VP16 for 30 min, 1 μM APH for 16 h, 300 μM hydrogen peroxide (H2O2) for 30 min. Hypoosmotic stress (hypo) was applied by incubation the cells in 50% DMEM/50% H2O (Hypoosmotic medium) for 30 min. To induce DNA double-strand breaks using the I-PpoI endonuclease, cells were transiently transfected with a plasmid encoding I-PpoI. After 24 hours, nuclear translocation of I-PpoI was triggered by treating the cells with 1 μM 4-hydroxytamoxifen (4-OHT) for 30 minutes. TOPBP1 protein levels were analyzed by Western blotting (WB). Histone H2B is used as a loading control in WB. **(F)** HeLa cells were transfected with TOPBP1-FLAG–FKBP12F36V (TOPBP1-FK; w/ TOPBP1-FK). Non-transfected cells (w/o TOPBP1-FK) were used as controls. Cells were lysed 24 h post-transfection, and TOPBP1 protein levels were analyzed by Western blotting (WB).

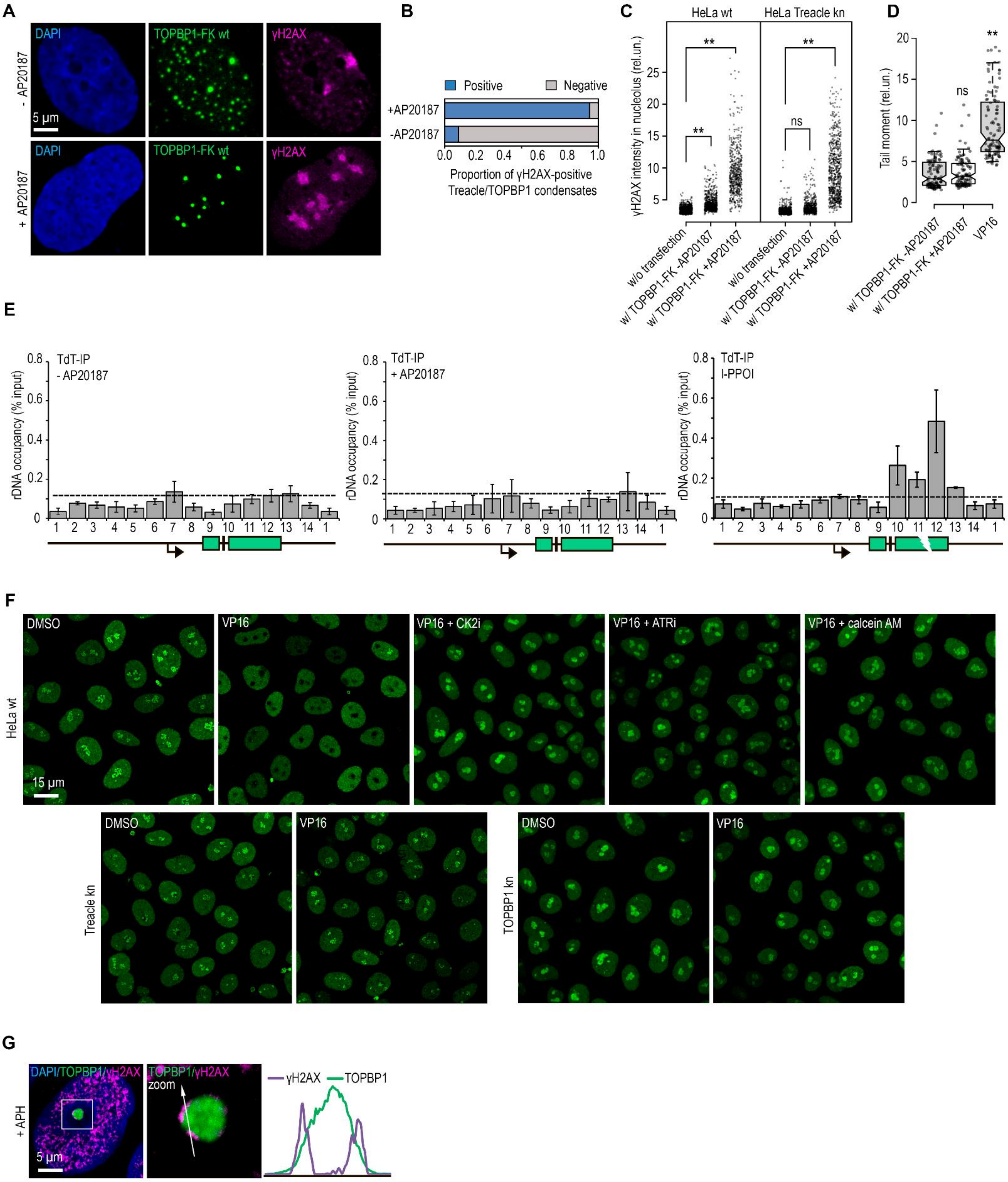

Figure S6: Treacle-dependent TOPBP1 condensation mediates nucleolar DNA damage response (n-DDR) activation and rDNA transcriptional repression under genotoxic stress **(A)** HeLa cells were transfected with TOPBP1-FLAF-FKBP12F36V (TOPBP1-FK). For induction of TOPBP1 oligomerization, transfected cells were incubated with 100 nM AP20187 for 24 h. The cells were fixed 24 hours after transfection, co-immunostained for FLAG antibodies (green) and γH2AX (magenta) and analyzed by laser scanning confocal microscopy. **(B)** HeLa

cells were processed as described in (A). Percentage of γH2AX-positive TOPBP1/Treacle-condensates is shown. **(C)** Hela Treacle kn cells or HeLa wt cells were transfected with TOPBP1-FLAF-FKBP12F36V (TOPBP1-FK). For induction of TOPBP1 oligomerization, transfected cells were incubated with 100 nM AP20187 for 24 h. The cells were fixed 24 hours after transfection, co-immunostained for FLAG antibodies (green) and γH2AX (magenta) and analyzed by laser scanning confocal microscopy. The γH2AX intensity per nucleus was measured (n>500). **, $p < 0.01$, n.s., not significant by unpaired t test. **(D)** HeLa cells were transfected with TOPBP1-FK. For induction of TOPBP1 oligomerization, transfected cells were incubated with 100 nM AP20187 for 24 h. For positive controls cells were treated 30 μM VP16 for 30 min. The neutral comet assay was performed; box plots show the tail moment. **, $p < 0.01$, n.s., not significant by unpaired t test. **(E)** HeLa cells were transfected with TOPBP1-FK. For induction of TOPBP1 oligomerization, transfected cells were incubated with 100 nM AP20187 for 24 h. For rDNA damage induction, HeLa cells were transfected with I-PpoI and treated the cells with 1 μM 4-hydroxytamoxifen (4-OHT) for 6 h. TdT-IP was followed by qPCR using the rDNA amplicons positioned as indicated on the scheme below the graphs. Data are represented relative to the input. Values are mean ± SD from at least three independent experiments. **(F)** Hela Treacle kn cells, Hela TOPBP1 kn cells or Hela wt cells pretreated with either 15 μM VE821 (ATRi) for 6 h, 30 μM CX-4945 (CK2i) for 3 h or 5 μM Calcein AM for 5 h were treated 90 μM VP16 for 60 min in combination with 100 μM ethynyl uridine (EU). DMSO-treated cells were used as controls. EU was revealed by click chemistry. **(G)** Hela cells were treated with 1 μM APH for 16 h. Cells were fixed and co-immunostained for TOPBP1 (green) and γH2AX (magenta) antibodies and analyzed by laser scanning confocal microscopy. DNA was stained with DAPI (blue). Co-localization analysis was performed on the merged images. Graphs illustrate quantification in arbitrary units of fluorescence distribution along the lines shown in the figures.

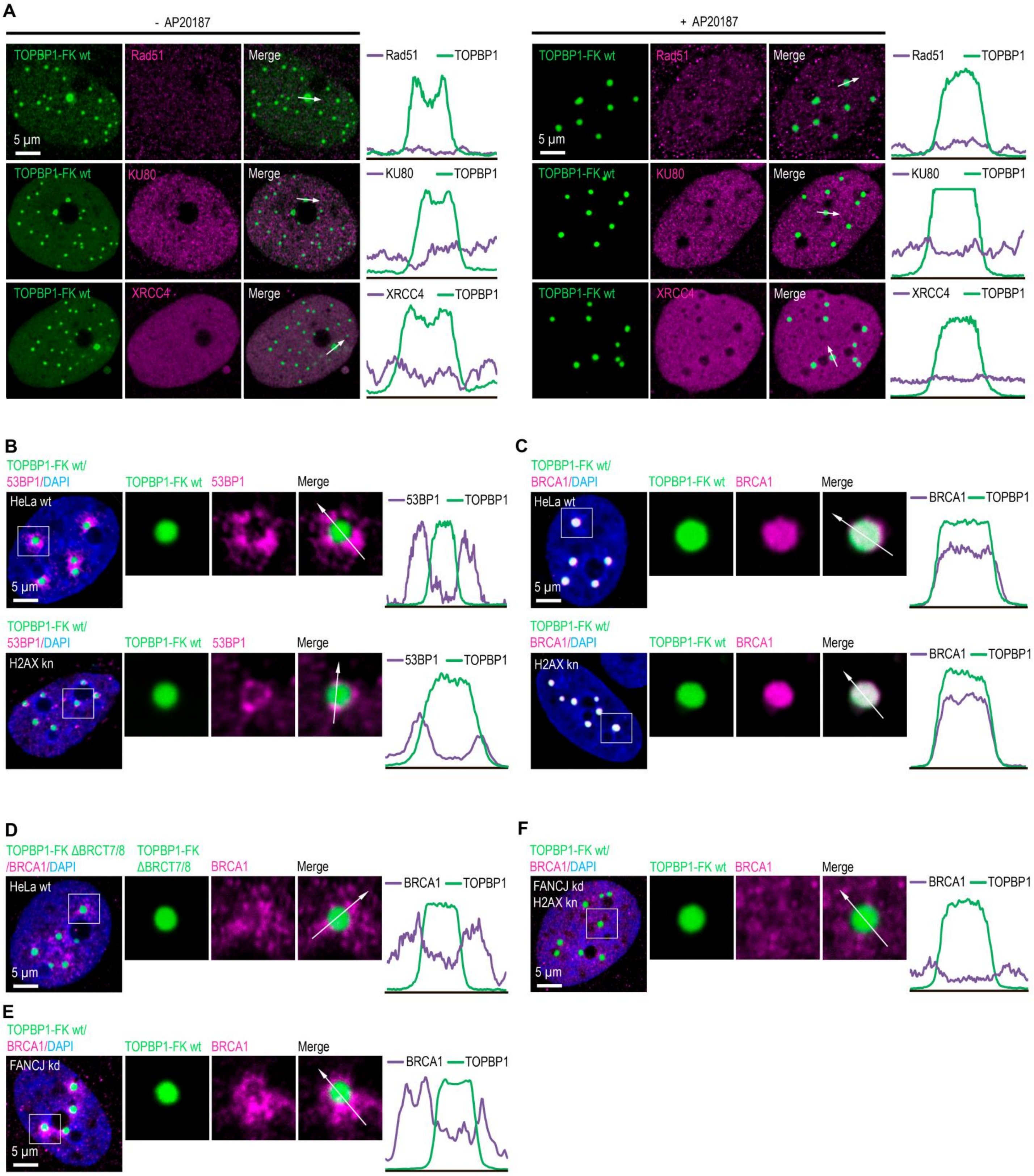

Figure S7: Treacle-dependent TOPBP1 condensation selectively recruits DNA repair factors 53BP1 and BRCA1 to nucleolar condensates and rDNA, depending on DNA damage context. **(A)** HeLa cells were transfected with TOPBP1-FLAF-FKBP12F36V (TOPBP1-FK). For induction of TOPBP1 oligomerization, transfected cells were incubated with 100 nM AP20187 for 24 h. The cells were fixed 24 hours after transfection, co-immunostained for FLAG antibodies (green) and either Rad51, KU80 or XRCC1 (magenta) and analyzed by laser scanning confocal

microscopy. Co-localization analysis was performed on the merged images. Graphs illustrate quantification in arbitrary units of fluorescence distribution along the lines shown in the figures. **(B)** HeLa wt cells or HeLa H2AX kn cells were transfected with TOPBP1-FLAF-FKBP12F36V (TOPBP1-FK). For induction of TOPBP1 oligomerization, transfected cells were incubated with 100 nM AP20187 for 24 h. The cells were fixed 24 hours after transfection, co-immunostained for FLAG antibodies (green) and 53BP1 (magenta) and analyzed by laser scanning confocal microscopy. **(C)** HeLa wt cells or HeLa H2AX kn cells were transfected with TOPBP1-FLAF-FKBP12F36V (TOPBP1-FK). For induction of TOPBP1 oligomerization, transfected cells were incubated with 100 nM AP20187 for 24 h. The cells were fixed 24 hours after transfection, co-immunostained for FLAG antibodies (green) and BRCA1 (magenta) and analyzed by laser scanning confocal microscopy. **(D)** HeLa wt cells were transfected with TOPBP1-FK ΔBRCT7/8. For induction of TOPBP1 oligomerization, transfected cells were incubated with 100 nM AP20187 for 24 h. The cells were fixed 24 hours after transfection, co-immunostained for FLAG antibodies (green) and BRCA1 (magenta) and analyzed by laser scanning confocal microscopy. **(E)** HeLa wt cells with *FANCJ* kd were transfected with TOPBP1-FK ΔBRCT7/8. For induction of TOPBP1 oligomerization, transfected cells were incubated with 100 nM AP20187 for 24 h. The cells were fixed 24 hours after transfection, co-immunostained for FLAG antibodies (green) and BRCA1 (magenta) and analyzed by laser scanning confocal microscopy. **(F)** HeLa H2AX kn cells with *FANCJ* kd were transfected with TOPBP1-FK ΔBRCT7/8. For induction of TOPBP1 oligomerization, transfected cells were incubated with 100 nM AP20187 for 24 h. The cells were fixed 24 hours after transfection, co-immunostained for FLAG antibodies (green) and BRCA1 (magenta) and analyzed by laser scanning confocal microscopy.

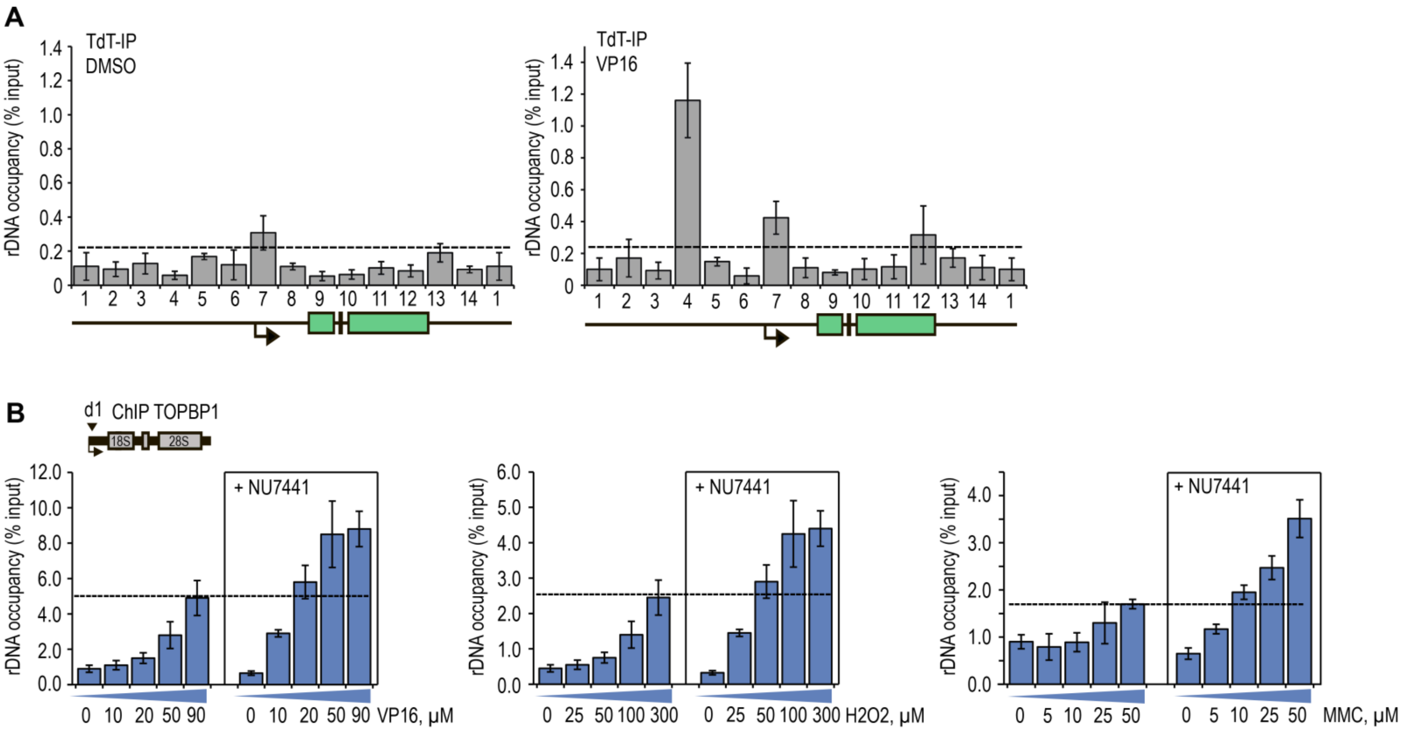

Figure S8: DNA-PK activity restrains TOPBP1 recruitment to rDNA in response to genotoxic stress. (A) HeLa cells were treated with 90 µM etoposide (VP16) for 30 min. TdT-IP was followed by qPCR using the rDNA amplicons positioned as indicated on the scheme below the graphs. Data are represented relative to the input. Values are mean ± SD from at least three independent experiments. **(B)** Control HeLa cells or cells pretreated for 3 h with the DNA-PK inhibitor NU7441 (10 µM) were exposed to increasing concentrations of etoposide (10–90 µM for 30 min), hydrogen peroxide (H_2O_2; 25–300 µM for 30 min), or mitomycin C (MMC; 5–50 µM for 60 min). ChIP experiments were performed with antibodies against TOPBP1. ChIP was followed by qPCR using the d1 primers to the promoter of the rRNA gene (positioned as indicated on the scheme). Data are represented relative to the input. Values are means ±SD from at least three independent replicates. **, $p < 0.01$, n.s., not significant by unpaired t test.

Supplementary Table 1. Primers used for cloning and generation of expression constructs.			
	forward (5'-3')	reverse (5'-3')	
Set#1 cloning	TATAAGATCTGTATGGCCGA GGCCAGGAA	TATAGGATCCTCATACAGT CTGCTCTGCTGTCTTC	Added restrictive sites are highlighted in red
Set#2 cloning	gccgccgaggatgatgtggtggcg	ctctgctgcggtctcctcca	
Set#3 cloning	gcccagtctctcctctcaggtt	tggcgccaccacatcatcctc	
Set#4 cloning	tataccgcggAAATGTCCAGAAAT GACAAAGAACC	tataccgcggTTAGTGTACTCTA GGTCGTTTGATTTTAT	
Set#5 cloning	gattacaaagacgatgacgacaagatgtccaga aatgacaaagaaccgttttttgtgaag	ggtggcgaccggtagcg	Added FLAG fragments are highlighted in red
Set#6 cloning	aaaggatccaccggatctagataactgatcataa tc	aaaccgcggtgtactctaggtcgtttgatttt attttttctgt	Added restrictive sites are highlighted in red
Set#7 cloning	aaaccgcggggagtgcaggtggaaaccatctc	aaaggatccttattccagttttagaagctcca catcgaaga	Added restrictive sites are highlighted in red. Added stop codon is highlighted in green.
Set#8 cloning	tgtcaggatgaatccatatacaagacaga	acacttgaaatcttccatgtttatatcag	
Set#9 cloning	agccattttctgattgaaaattcaac	agtcattcctgtcattactggaactg	
Set#10 cloning	tcacctcctcatgtagaaaattactgtc	ttcttcatgagtctcctctatcg	
Set#11 cloning	gaaattgctaaacaggctgtctgtg	ggcacaatctaaaagccagtgctc	

Supplementary Table 2. Primers used for cloning guide RNAs for the CRISPR–Cas9 system.			
	sence (5'-3')	anti-sence (5'-3')	
sgRNA1 TOPBP1	CACCGAAACTGGATGTTCGG CTCTT	AAACAAGAGCCGAACATC CAGTTTC	Added restrictive sites are highlighted in red
sgRNA2 TOPBP1	CACCGATATATCTTTGCGGT TTTAG	AAACCTAAAACCGCAAAG ATATATC	
sgRNA1 Treacle	CACCGAAGTAGCTCCCGCCG CTTCC	AAACGGAAGCGGCGGGAG CTACTTC	
sgRNA2 Treacle	CACCGGGGGTCGCGGGTATG GCCG	AAACCGGCCATACCCGCGA CCCCC	
sgRNA1 H2AX	CACCATGGCCAGCTGCAGGT GGCG	AAACCGCCACCTGCAGCTG GCCAT	
sgRNA2 H2AX	CACCCGAGGAGCTCAACAA GCTGC	AAACGCAGCTTGTTGAGCT CCTCG	

Supplementary Table 3. Antibodies used in this study.			
Antibody	Source	Cat. #	Applications
anti-TopBP1, mouse	Santa Cruz Biotechnology	sc-271043	ICC, ChIP, ChIP-seq, WB, PLA
anti-TopBP1, rabbit	Abcam	ab105109	ICC
anti-Treacle/TCOF1, rabbit	Sigma-Aldrich	HPA038237	ICC, ChIP, ChIP-seq, WB, PLA

anti-FLAG, mouse	Sigma-Aldrich	F1804	ICC, ChIP
anti-Katushka2S, rabbit	Eurogene	AB233	ChIP
anti-γH2AX (Ser139), mouse	Millipore	05-636	ICC, ChIP
anti-pATR (Thr1989), rabbit	Cell Signaling	58014	ICC, ChIP
anti-pATM (Ser1981), mouse	Cell Signaling	4526	ICC, ChIP
anti-pDNAPK (Ser2056), rabbit	Abcam	ab124918	ChIP
anti-BRCA1, mouse	Santa Cruz Biotechnology	sc-6954	ICC, ChIP
anti-53BP1, rabbit	Santa Cruz Biotechnology	sc-22760	ICC, ChIP
anti-Rad21, rabbit	Abcam	ab992	ChIP-seq
Polyclonal Goat anti-mouse Alexa Fluor Plus 488	Invitrogene	A32723	ICC
Polyclonal Goat anti-mouse Alexa Fluor Plus 594	Invitrogene	A32742	ICC
Polyclonal Goat anti-rabbit CF488A	Biotium	20012	ICC
Polyclonal Goat anti-rabbit CF594	Biotium	20112	ICC

**Supplementary Table 4. Primers used for ChIP–qPCR at rDNA and ALU repeat regions.**		
	forward (5'-3')	reverse (5'-3')
ChIP-qPCR d1	GGTATATCTTTCGCTCCGAGTC	ACAGGTCGCCAGAGGACAG
ChIP-qPCR d3	CAGCGTGTGCCTACCCTAC	TCCCTCGTTCATGGGGAATAA
ChIP-qPCR d5	TCCCTCCGAAGTTTCCCTCA	CGGCCCCAAGACCTCTAATC
ChIP-qPCR d7	CGCCTGGTCTTCTGTCTCTG	CGTAAGCTGGAGTGGAAGTGT
ChIP-qPCR ALU	ACCATCCCGGCTAAAACGGTGA	GCGATCTCGGCTCACTG

**Supplementary Table 5. Primers used for qPCR in the TdT–IP assay at rDNA.**		
	forward (5'-3')	reverse (5'-3')
1	TGGCCCTTACGCTCAGAATG	GAGTTTGGCTCTTGCTGCC
2	GCTTCTCCCTCGACTGTCTC	GGCTCGGTCACAGATCACTT
3	AGTGAAGACAACTCACGCCC	CTGCAAACGGGAACACGA
4	AATGGCCTTAGCCCTGGTG	GTCCAGAGACGAGAGACCGA
5	GGCCCGATTGTTCTTCTCCT	CTGCCATCTGTCAAACCCGA
6	GACAACGTGTATCTCTGCATT	ACACAGACACCTACATCTATCA
7	GGTATATCTTTCGCTCCGAGTC	ACAGGTCGCCAGAGGACAG
8	GCCTTCTCTAGCGATCTGAGAG	CCATAACGGAGGCAGAGACA
9	CAGCGTGTGCCTACCCTAC	TCCCTCGTTCATGGGGAATAA
10	GGTTGCTTGGGAATGCAG	CTTGTTGACTATCGGTCTCGTG

11	TCCCTCCGAAGTTTCCCTCA	CGGCCCCAAGACCTCTAATC
12	TGGCGCTAAACCATTCGTAG	GTCGAGGGCTGACTTTCAATA G
13	CGCCTGGTCTTCTGTCTCTG	CGTAAGCTGGAGTGGAAGTGT
14	TTGACGTACAGGGTGGACTG	GGCATCCTAGGTGACATTTCC

**Supplementary Table 6. Primers used for qPCR in the TdT–IP assay at rDNA CTCF sites.**		
	forward (5'-3')	reverse (5'-3')
rCTCF1	TCCCTCCCTGCTTCCGAG	ACACAAACGGTGCCCAAAAC
rCTCF2	AGCCTCCATCCTTCCCCC	TGGAGAAGACACAGTTGCCC
rCTCF3	TCCCTCCCTGCTTCCGAG	ACACAAACGGTGCCCAAAAC
